# Macrocyclic Chelators for Aqueous Lanthanide Separations via Precipitation: Toward Sustainable Recycling of Rare-Earths from NdFeB Magnets

**DOI:** 10.1021/jacs.5c04150

**Published:** 2025-06-19

**Authors:** Kelsea G. Jones, Tomáš David, Martin Loula, Stanislava Matějková, Jan Blahut, Anatolij Filimoněnko, Miroslava Litecká, Jan Rohlíček, Jiří Böserle, Miloslav Polasek

**Affiliations:** † Institute of Organic Chemistry and Biochemistry, 89220 Czech Academy of Sciences, Flemingovo náměstí 542/2, 160 00 Prague 6, Czech Republic; ‡ Department of Inorganic Chemistry, Faculty of Science, Charles University, Hlavova 2030, 128 40 Prague 2, Czech Republic; § Institute of Inorganic Chemistry, 112895 Czech Academy of Sciences, Husinec-Řež 1001, 250 68 Husinec-Řež, Czech Republic; ∥ Institute of Physics, Czech Academy of Sciences, Na Slovance 1999/2, 182 00 Prague 8, Czech Republic; ⊥ Department of Applied Electronics, Faculty of Electrical Engineering and Computer Science, VSB—Technical University of Ostrava, 17. listopadu 2172/15, 708 00 Ostrava, Czech Republic

## Abstract

Rare-earth elements (REEs) are critical materials in modern industry, but their production has a significant environmental footprint. Environmentally friendly separation methods would enable efficient, sustainable recycling of REEs. This work introduces a class of cyclen-based macrocyclic chelators that induce significant differences in solubility for REE chelates, enabling their selective precipitation from pH-neutral aqueous solution. The process was refined using simple coordinating additives (e.g., acetate) to form ternary coordination compounds to fine-tune these chelate solubilities. Conditions were optimized for the REEs found in NdFeB magnets, allowing separations of even adjacent lanthanides by repeated precipitations. Separation factors comparable to those of industrial solvent extraction methods were achieved without organic solvents. Analysis of NdFeB magnets from current electric car motors revealed an unexpected presence of holmium as a supplement and/or replacement for terbium and dysprosium, suggesting shifting industrial trends with implications for future recycling efforts. In a case study, one such automotive magnet was processed to obtain a 99.7% pure neodymium product. Scalable, tunable, and entirely aqueous, this approach advances the sustainable use of REEs toward a circular economy.

## Graphical abstract

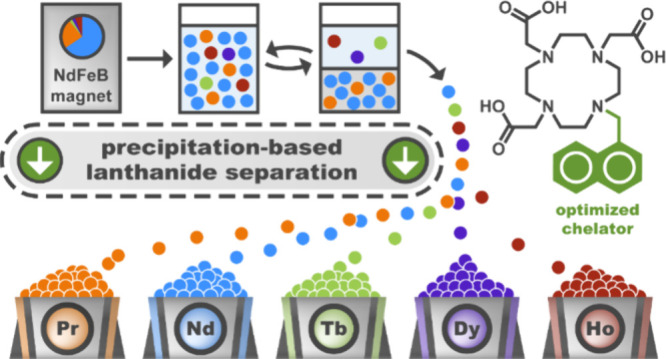

## Introduction

It is a paradox of modern industry that the very materials critical for a sustainable future come at great environmental cost. The rare-earth elements (REEs)—a group comprised of Sc, Y, and the lanthanides (Lns)—are a prime example of this. These elements are essential for applications ranging from medicine to electronics to renewable energy.^1–5^ Global demand for REEs is primarily driven by neodymium-based NdFeB magnets, which enable the efficient interconversion of kinetic and electric energy, and so are key components of wind turbines and electric vehicles.^4–7^ As these green technologies expand, REE demand will only continue to rise. Unfortunately, the global REE supply chain relies on virgin mining, which comes with significant environmental harm and a high carbon footprint.^8^ A more-sustainable alternative is urban mining: REEs can be recovered from NdFeB magnets in end-of-life products,^5–7,9–11^ and electric vehicles, in particular, represent a major reservoir of REEs that will need to be efficiently reclaimed.^6,8^

NdFeB magnets, despite their name, nearly always contain some combination of lanthanides, typically in the order Nd > Pr > Dy ≈ Tb.^5–7,9^ The exact composition varies by application; the rarer and costlier Dy and Tb are required for high-performance applications, such as electric car motors. Due to this variable composition, any recycling methods must include some degree of Ln separation. And while lanthanides as a group can be readily isolated from the other elements present in NdFeB magnets, separating them from one another remains notoriously challenging.^1,12,13^

The primary industrial method of Ln separations—solvent extraction (SX)—is based on simple lipophilic ligands which weakly coordinate the trivalent lanthanides.^14,15^ The binding affinities vary slightly across the Ln^3+^ series due to the lanthanide contraction (i.e., the decreasing ionic radius across the series),^16^ and affect the distribution of Lns between acidic water and an organic solvent. These differences are small, however, and necessitate numerous repetitions for complete separation,^1,14^ in turn requiring extensive infrastructures and large quantities of acids and organic solvents.^1,12,13,15^ Despite this, SX remains the industry standard, because it enables robust, scalable separations of all lanthanides, and efforts are ongoing to develop more selective extractant ligands.^17–20^ The only industrial alternative to SX is ion-exchange chromatography, which achieves efficient separations under more-benign aqueous conditions, albeit with limited throughput. It is therefore suited only to smaller-scale, high-purity applications.^13,21^ In order to facilitate the recycling of Lns, innovative separation strategies must strike a balance between sustainability and scalability.

The first lanthanide separations, historically, were based on differences in solubility.^1^ Such methods are now seeing a resurgence, though they employ much more advanced chemical systems. Improved selectivity is achieved today by compounds ranging from peptides^22^ and inorganic borates^23^ to small ligands^24–27^ and organic chelators,^28–36^ with the latter gaining a particular traction. Multidentate chelators can be tuned to amplify small differences in Ln^3+^ size, triggering structural changes upon coordination to yield distinct differences in solubility for the Ln chelates.^28–36^ Reported examples include the tripodal TriNOx,^28–30^ tren-1,2,3-HOPO,^31^ Schiff base,^32^ Trensal^R^,^33^ and amido-arene^34,35^ systems, as well as the macrocyclic G-macropa.^36^ These have all enabled impressive single-step separations of light and heavy lanthanides, often demonstrated with Nd and Dy. However, significant drawbacks remain: these systems require organic or strongly acidic solvents, a high excess of chelator, and/or complicated syntheses. Additionally, the fixed selectivity of these tailored molecules limits their adaptability for separating other Lns from NdFeB magnets, including the adjacent Pr/Nd and Tb/Dy pairs.

This work reports a family of macrocyclic DO3A-type chelators (Figure 1) with unexpected solubility trends across the series of Ln^3+^ chelates in pH-neutral aqueous solutions. By optimizing the chelator structure, coordinating additive, and reaction conditions, all Ln pairs relevant to NdFeB magnets may be separated by selective precipitation from water (Figure 2). This system shares key advantages with SX, such as being scalable and adaptable for adjacent lanthanide pairs, while removing the reliance on organic solvents. As a case study, a complete processing scheme was demonstrated on an NdFeB magnet from the motor of an electric car, culminating in a 99.7% Nd product.

**Figure 1:**
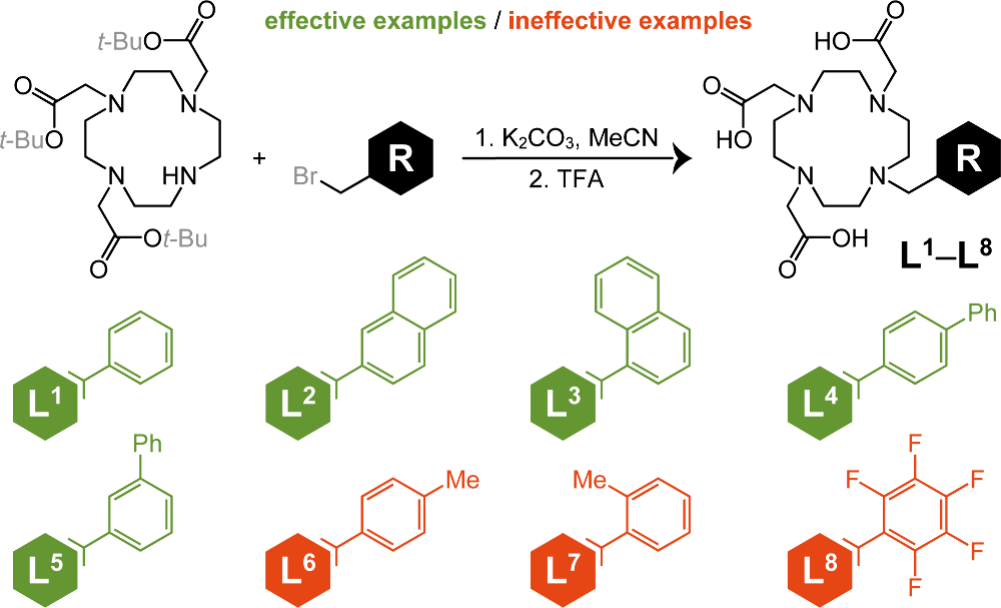
Chelators investigated in this work. A general synthetic procedure yielded eight chelators bearing various aromatic arms. Green: L^1^–L^5^; effective examples with confirmed selective solubility trends across the Ln^3+^ chelate series. Red: L^6^–L^8^; ineffective examples yielding no observed precipitation.

**Figure 2:**
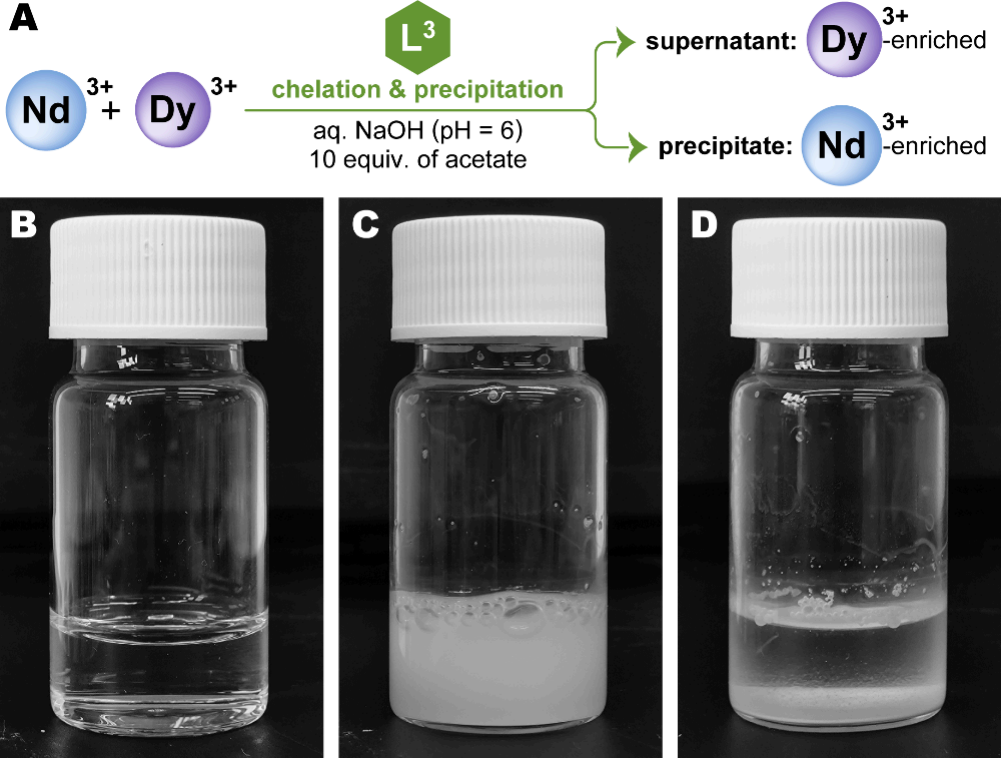
Nd/Dy separation by precipitation from water. (A) A general scheme of the separation. (B) A mildly acidic aqueous solution of chelator L^3^, Nd^3+^, Dy^3+^, and acetate additive was prepared. (C) Titration with NaOH to pH = 6 afforded [Ln­(L^3^)] chelates that promptly partially precipitated. (D) The mixture separated into Nd-enriched precipitate and Dy-enriched supernatant.

## Results and Discussion

All chelators were prepared in good yield via the general *t*-Bu_3_DO3A alkylation scheme, followed by deprotection and preparative HPLC (high-performance liquid chromatography) purification (Figure 1; see Figures S1–S9 for detailed procedures).

### Chelator Screening and Selection

During the exploratory phase of an unrelated project, a simple DO3A-benzyl chelator (L^1^, Figure 1) presented surprising solubility trend across the series of Ln^3+^ chelates. (Henceforth, ‘lanthanide’ and ‘Ln’ will include Sc and Y.) Instead of the gradual changes expected due to the lanthanide contraction, the L^1^ chelates of Gd^3+^, Tb^3+^, Dy^3+^, and Y^3+^ (comparable in size to Dy^3+^) precipitated heavily from aqueous solution, while the other Ln^3+^ chelates remained fully soluble at 5 mM (Figure S10).

Chelators of the DO3A/DOTA family have thus far not been considered for solubility-based Ln separation methods^37^—likely due to their primarily biomedical applications, which require well-soluble chelates.^2^ Previously, one study of [Gd­(L^1^)] mentioned its poor solubility as an unfavorable property.^38^ Other structurally related chelators, such as DO3A derivatives with 4-carboxy-benzyl^39^ or naphthyl^40^ arms, a methylated biphenyl-armed DO3A analogue with one phosphonate,^41^ and systems of two DO3A units connected through aromatic linkers^42,43^ were reported to form lanthanide chelates which aggregated or precipitated, but any potential utility for Ln separations was not explored. The primary structural feature setting these examples apart from other known (and highly soluble) DO3A derivatives is the presence of a noncoordinating lipophilic, aromatic arm (e.g., benzyl). It seemed likely that this moiety was responsible for the unusual solubility behavior of [Ln­(L^1^)], so a library of chelators containing variations of this group was investigated (L^1^–L^8^, Figure 1).

For each chelator, the series of 16 Ln^3+^ chelates was prepared as 5 mM aqueous solutions, stirred overnight to induce precipitation, and analyzed via HPLC to quantify the soluble fraction (Figure 3). These results confirmed that the Ln-series solubility profile strongly depends on the structure of the chelator and its aromatic arm.

**Figure 3:**
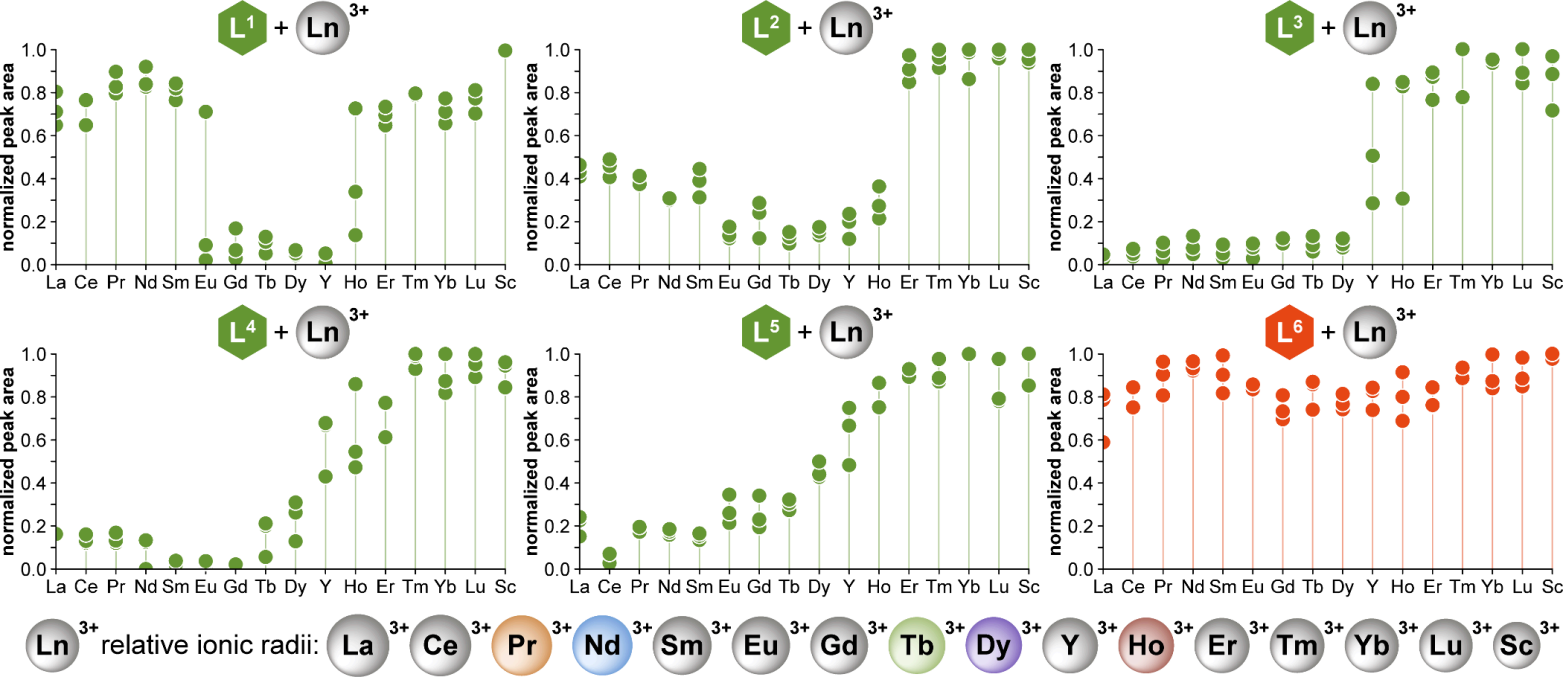
Solubility profiles of Ln^3+^ chelates formed with chelators L^1^–L^6^ across the lanthanide series. Plots show the relative quantity of Ln chelate remaining in solution after precipitation, as determined by HPLC analysis; UV peak areas were normalized to the largest value for a fully soluble chelate within a given series. Generally, values <0.6 corresponded with the presence of visible precipitate. Chelators L^1^–L^5^ yielded distinct solubility profiles, with clear precipitation of some Ln chelates. No precipitation was observed for L^6^ (and likewise L^7^, L^8^; see Figure S11); fluctuations in peak areas therefore represent the experimental error. Each series was prepared in triplicate. *Conditions and analysis:* 100-μL reactions were prepared in 96-well plates (5 mM Ln^3+^, 5.5 mM chelator, and 500 mM MOPS pH 7 buffer) and were stirred for 16 h, then centrifuged. The supernatants were analyzed by HPLC. Further experimental details, as well as data for L^7^ and L^8^, are given in Figure S11. Below the plots, the relative size of Ln^3+^ ions is shown for reference, ordered by decreasing size, with Y^3+^ and Sc^3+^ positioned accordingly;^44^ radioactive Pm^3+^ was excluded. Those elements most relevant to NdFeB magnets and studied extensively in this work are highlighted in color.

Generally, chelates of the late/heavy lanthanides (Er–Lu; Sc) remained soluble in all cases, while the solubilities of the others varied. The most striking profile was that of L^1^, which precipitated only the middle Lns, though this proved difficult to control (see further). For the naphthyl (L^2^, L^3^) and biphenyl (L^4^, L^5^) derivatives, chelates of both the early/light and middle Lns precipitated. Their solubility profiles differed, however, even between isomer pairs (compare L^2^/L^3^, and L^4^/L^5^). Notably, the naphthyl derivatives exhibited sharp solubility transitions between the middle and heavy lanthanides, making them more favorable for separations than the biphenyl analogues. In these regions of steep solubility change, repeated tests sometimes varied between full solubility and precipitation, suggesting supersaturated solutions awaiting nucleation events. This inconsistency was most pronounced with L^1^, where interexperiment reproducibility was poor for chelates of Pr–Eu, rendering this chelator impractical. Moreover, the methyl- and fluoro-derivatives of L^1^ (i.e., L^6^–L^8^) showed that even small modifications to the benzyl arm interfered with and thwarted precipitation (Figures 3 and S11). By contrast, the more-lipophilic naphthyl- and biphenyl-armed chelators demonstrated robust performance.

The chelator structure affected, too, the kinetics of precipitation. Chelates of L^1^ were the slowest to precipitate, while the least-soluble chelates of the naphthyl and biphenyl derivatives precipitated almost immediately upon complexation. Given the focus on NdFeB magnets, the behavior of the Nd chelates was of primary interest. The differences in kinetics are apparent from the comparison of [Nd­(L^1^)] and [Nd­(L^3^)] (Figure S12). The former required hours to even begin to precipitate, while the latter achieved 90% precipitation within 15 min. Due to its favorable solubility profile, consistent behavior, and rapid precipitation, L^3^ was selected for further study.

### Solid-State Studies and Probable Mechanism

Precipitation must be initiated by binding forces between individual chelate units. Based on the hypothesis that intermolecular interactions were responsible for the selective precipitation of the [Ln­(L^3^)] chelates, their structures were investigated using a range of techniques. However, obtaining detailed structural information from the most relevant material—the [Ln­(L^3^)] precipitate itself—proved challenging. Electron microscopy revealed nanometer-sized crystals aggregated into micrometer-scale polycrystalline clumps (Figure S13). The small crystal domain size caused substantial broadening of the diffraction lines in powder X-ray diffraction of the [Nd­(L^3^)] precipitate, preventing detailed structural analysis (Figure S14). Solid-state NMR experiments designed to detect functional groups potentially involved in Ln bridging, such as the chelator acetate arms or coordinated H_2_O/OH^–^, were inconclusive due to broad signals (Figures S15–S17).

After considerable effort, a specimen of [Dy­(L^3^)] suitable for single-crystal X-ray analysis provided key insights (Figure 4, S18, S19). Structural data revealed that the chelate units are connected via coordination bridging, in which the acetate arm of one unit coordinates both its own Ln center and that of a neighboring unit. This results in a symmetrical triangular arrangement of three interlinked chelates. The structure is further stabilized by hydrogen bonding between the Ln-coordinated water molecule of one unit and the bridging acetate oxygen of an adjacent unit. The single-crystal [Dy­(L^3^)] structure was remarkably consistent with powder X-ray data for [Nd­(L^3^)], suggesting that this trimeric structure applies to the precipitate across a relevant range of the lanthanide series (Figure S20). Interestingly, the aromatic arms did not engage in any significant π-π stacking interactions that might indicate that they play a directing role in the crystal packing.

**Figure 4:**
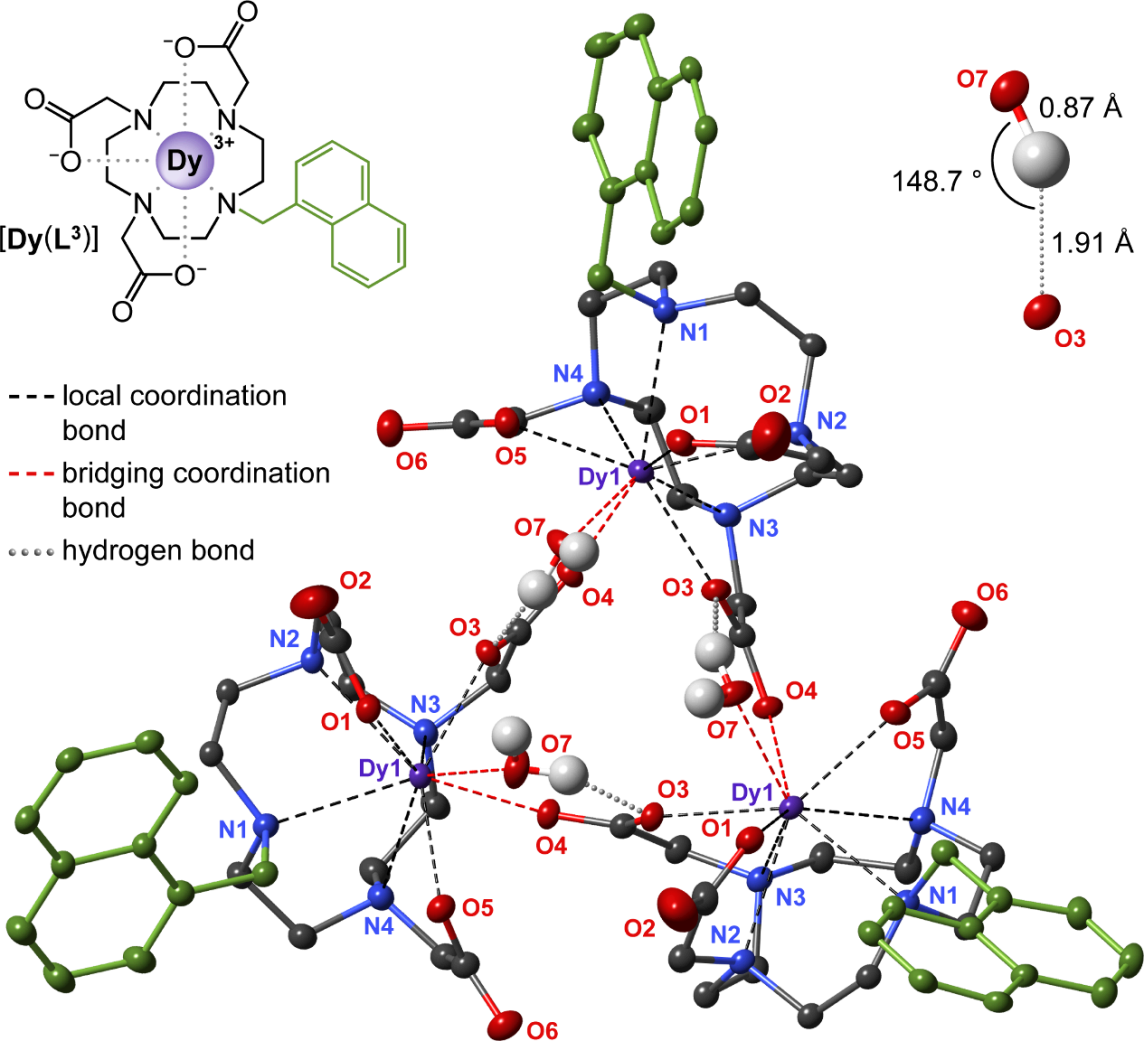
Symmetric trimer {[Dy­(L^3^)­(H_2_O)]}_3_ found in the single-crystal X-ray structure of {[Dy­(L^3^)­(H_2_O)]}_3_·16.2H_2_O. The trimeric arrangement is stabilized by the coordination of carboxylate moieties from the neighboring unit and bridging H_2_O molecules involving an intermolecular hydrogen bond (detailed in top right inset). The naphthalene pendant arms point away from the trimer center. The trimeric units are interconnected by a rich network of hydrogen bonds via H_2_O molecules (not shown, both for clarity and due to their disorder). The crystal packing features no obvious π–π stacking of the naphthalene moieties. Carbon-bound hydrogen atoms and labels of carbon atoms were omitted for clarity. Thermal ellipsoids were set at 25% probability for better view. The fully labeled structure and selected crystallographic parameters are given in Figure S19.

The structure of the {[Dy­(L^3^)­(H_2_O)]}_3_ trimer may be compared to related, previously studied systems. The poorly soluble [Tb­(L^1^)] chelate showed similar acetate-arm bridging, forming cyclic tetramers (Regueiro-Figueroa et al.).^38^ A para-carboxylated derivative of L^1^ gave soluble dimers via intermolecular coordination of that carboxyl group to another Ln center (Faulkner and Burton-Pye).^39^ Other studies reported self-aggregation of dinuclear Ln chelates containing aromatic linkers between two DO3A units and attributed this phenomenon to intermolecular π-π interactions (Regueiro-Figueroa et al. and Costa et al.).^42,43^ However, in light of both current and previous findings, coordination bridging appears to be a more plausible explanation. Taken altogether, these examples implicate coordination bridging as a key factor in the aggregation and reduced solubility of aromatic-armed DO3A lanthanide chelates.

Several lines of indirect evidence support the hypothesis that coordination bridging is responsible for the observed selective solubility of the [Ln­(L^3^)] chelates. First, the lanthanide contraction is known to reduce the coordination number in DOTA/DO3A chelates across the series from 9 to 8.^45,46^ In the {[Dy­(L^3^)­(H_2_O)]}_3_ structure found here, Dy^3+^ is 9-coordinate, with 7 donor atoms from the chelator, one coordinated water, and one bridging acetate arm. Under aqueous conditions, the smaller, heavier Ln^3+^ ions are expected to maintain the water ligand but lose the bulkier bridging acetate. This correlates with the high solubility of heavy Ln^3+^ chelates (Figure 3). Second, coordinating additives increase chelate solubility, likely by disrupting that bridging (see further). Third, the trimeric structure (Figure 4) reveals how bridging may reduce solubility: trimerization buries the hydrophilic sites while exposing the lipophilic naphthalene arms, likely reducing solvation. Whether bridging precedes or accompanies crystallization remains unclear.

Beyond hydration effects, the aromatic arms likely play a subtler role in precipitation. Even without strong π-π interactions, the size, shape, and orientation of the arm influence crystal packing. In the simple benzyl derivative L^1^, these factors are only borderline sufficient to induce precipitation, as evidenced by its slow kinetics and inconsistent behavior. In derivatives L^6^–L^8^, additional substituents on the benzyl arm likely interfere with packing, which explains why none of their chelates precipitated.

### Coordinating Additives Improving Separations

The separation of lanthanides by selective precipitation poses a fundamental challenge: while Ln chelates in solution behave as discrete, independent entities, the growth of a microcrystalline precipitate involves interactions between chelate units. Errors in crystal lattice assembly may incorporate significant amounts of the soluble Ln chelate into the matrix of the insoluble chelate. The behavior of a mixed-Ln system, therefore, cannot be inferred from the behavior of a single-Ln system. For considerations of NdFeB magnet recycling, it was important to evaluate the effect of an Nd-dominated chelate matrix on the full series of Ln chelates; chelator L^3^ was therefore tested with binary Nd/Ln mixtures. Significant coprecipitation was observed for those Lns which gave soluble chelates in isolation, but were near the break point of the solubility profile for L^3^—namely Y, Ho, and Er (Figure 5A-B).

**Figure 5:**
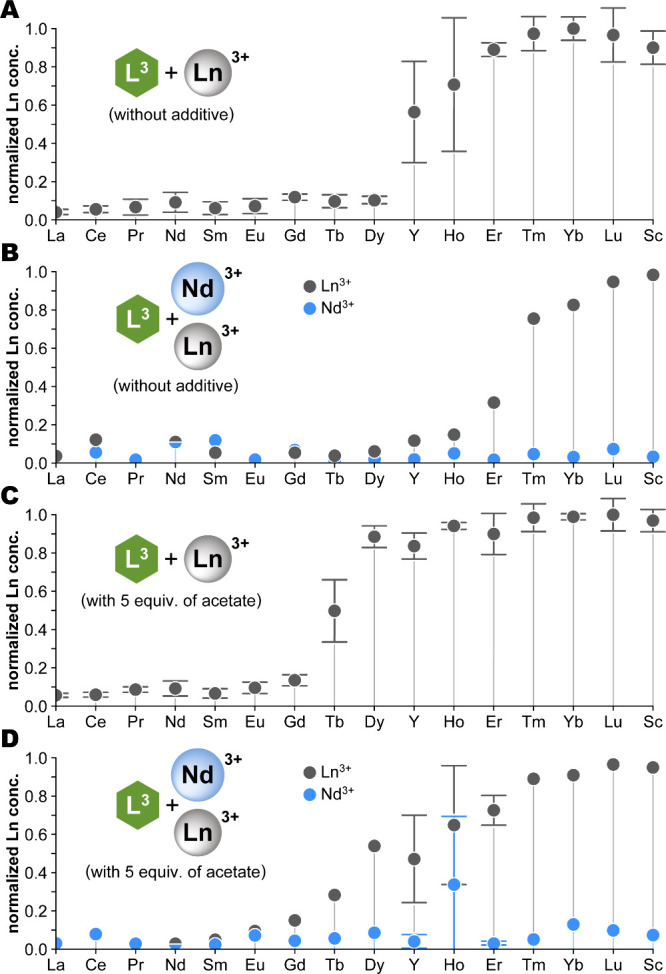
Solubility profiles and Ln/Nd separations with L^3^, and the effect of the acetate additive. (A) L^3^ solubility profile (no additive) given for reference, as the averaged data of Figure 3; (B) binary L^3^-Nd/Ln separations (no additive), showing that the precipitating [Nd­(L^3^)] causes significant coprecipitation of the Y, Ho, and Er chelates; (C) L^3^ solubility profile (5 equiv of acetate added), showing increased solubility for Tb–Ho; (D) binary L^3^-Nd/Ln separations (5 equiv of acetate added) maintain the increased solubility of Tb–Ho, even in the presence of the precipitating [Nd­(L^3^)] chelate. In (D), the Y, Ho, and Er samples were replicated due to the outlier behavior of Ho; see Figure S21. Data points with error bars represent the averages and standard deviations of triplicates. *Conditions and analysis:* Single-Ln solubility profiles were prepared and analyzed by HPLC as detailed in Figure 3, with 0 or 25 mM acetate, respectively. Binary Nd/Ln series were prepared analogously (5 mM Nd^3+^, 5 mM Ln^3+^, 11 mM L^3^, 500 mM MOPS pH 7 buffer, and either 0 or 50 mM acetate), and the supernatants were analyzed by ICP-OES for Ln quantitation.

Although this coprecipitation initially discouraged any prospect of achieving Ln separations with this system, these results in fact underscored the critical role of interchelate interactions in the process, indicating a possible path forward: perhaps, by modulating the coordination sphere of the Ln centers, the interchelate interactions could be selectively hindered, and the precipitation controlled.

*O-*Donor ligands (e.g., carbonate, lactate, or simple carboxylic acids) are well-known to form ternary coordination compounds with DO3A-type Ln chelates by occupying coordination sites not saturated by the chelator.^47–49^ The simple, readily available acetate was therefore chosen for a preliminary testing of this hypothesis. The solubility profiles of L^3^ for both the series of individual Ln chelates and the binary Nd/Ln series were reassessed with the addition of 5 equiv of acetate. This simple modification efficiently increased the solubilities of the middle-Ln chelates (Tb, Dy, Y, and Ho), and countered the coprecipitation caused by the presence of Nd (Figure 5C–D). This ternary-coordination-compound strategy thus enabled meaningful separations of those Lns relevant to NdFeB magnets, and encouraged further investigation.

A range of structurally diverse and potentially coordinating additives were tested for their effect on Nd/Dy separations with the L^3^ system, at various additive-to-chelate ratios. The Nd/Dy separation factor (SF, Figure 6) served as a benchmark; SF values of ca. 40 were achieved with several additives, though very different quantities of each were required. Bidentate ligands forming five-membered chelate rings (e.g., α-HIBA, glycolate, lactate) proved highly effective at nearly stoichiometric ratios due to their strong binding affinities, while the simpler carboxylic acids (e.g., formate, acetate, benzoate) were required in larger excesses for comparable results. Noncoordinating anions (e.g., thiocyanate, chloride, sulfate) had negligible effects even at 100 equiv. Data for three representative additives—acetate, α-HIBA, and chloride—are shown in Figure 6; Table S1 gives the full data set. Acetate emerged as the best choice, due to its high efficacy, small size, low cost, and eco-friendliness. For Nd/Dy separations, acetate was most effective at 10 equiv; higher amounts progressively solubilized the less-soluble [Nd­(L^3^)] until no precipitation (and hence, no separation) was observed (Figure 6). This sensitivity to the quantity of additive suggested that the system could be adapted to other Ln pairs.

**Figure 6:**
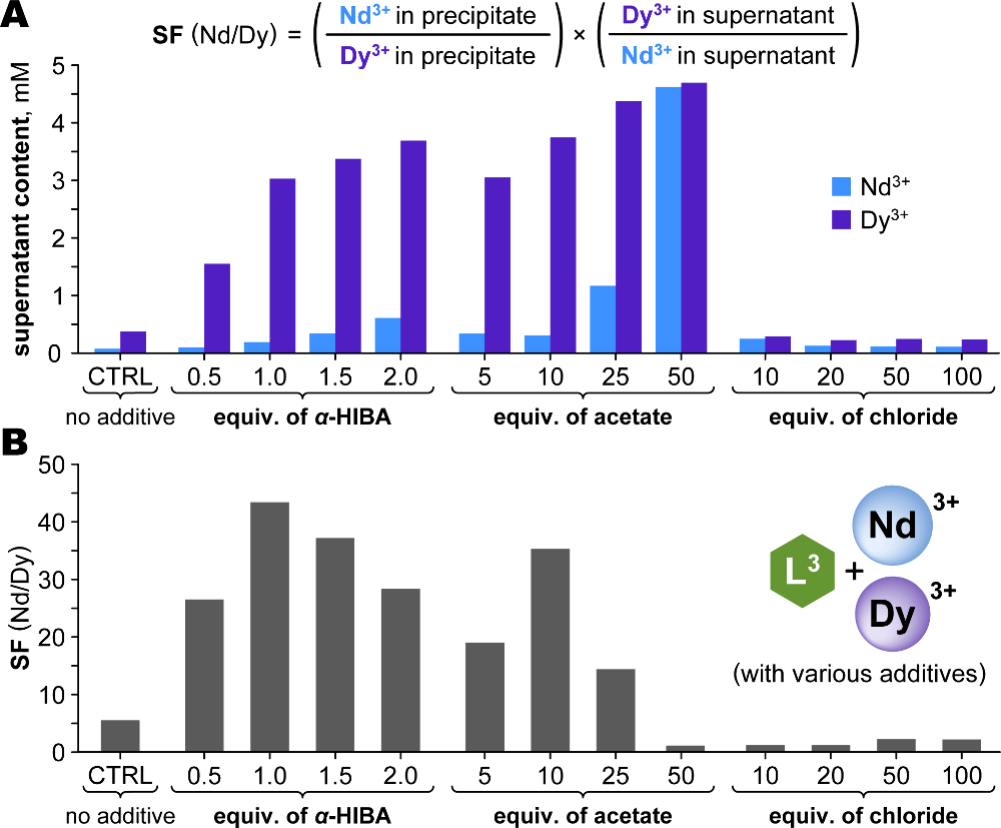
Selected results of additive screenings for Nd/Dy separations with L^3^. (A) The concentrations of Nd and Dy remaining in solution after precipitation show the effect of both the choice and quantity of additive; α-HIBA and acetate were considered highly effective, while chloride had no noticeable effect. Above, definition of the separation factor (SF) calculated from these concentrations is provided. (B) SF values show that optimal performance is achieved with specific amounts of additive, beyond which the separation worsens. *Conditions and analysis:* Aqueous reaction solutions (5 mM Nd^3+^, 5 mM Dy^3+^, 11 mM L^3^, 500 mM MOPS pH 7 buffer; for the additives, 1 equiv. = 10 mM) were stirred overnight at ambient conditions, then centrifuged; the Ln contents in both the precipitate and supernatant were determined by ICP-OES. The full data set and experimental details are given in Table S1.

The reaction duration also proved to be an important factor to consider. Monitoring the progress of Nd/Dy separation in the L^3^-Ln-acetate system revealed that acetate slowed the precipitation overall, but that [Nd­(L^3^)] precipitated much faster than [Dy­(L^3^)] (Figure 7). Excellent separations were observed within 3 h (SF = 42); thereafter, improvements were marginal. Effective separations can thus be achieved well before thermodynamic equilibrium, emphasizing the importance of timing for balancing purity, yield, and throughput.

**Figure 7:**
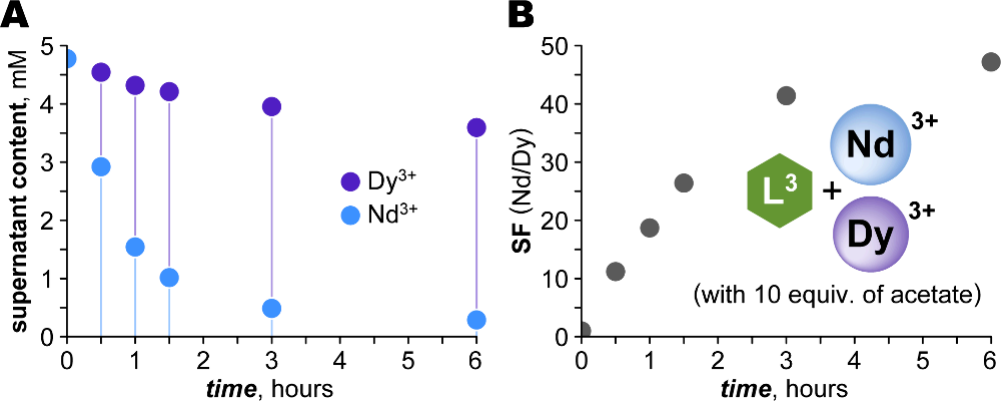
Kinetics of Nd/Dy separation with the L^3^-Ln-acetate system. (A) The concentrations of Nd and Dy remaining in solution over time. (B) The Nd/Dy separation factor improved dramatically over the first 3 h, and showed minimal change thereafter. *Conditions and analysis*: The aqueous solution (5 mM Nd^3+^, 5 mM Dy^3+^, 11 mM L^3^, 10 equiv. = 100 mM acetate) was titrated to pH 6 by NaOH and stirred. At intervals, samples of the supernatant were isolated by filtration, and Ln content was determined by ICP-OES.

### Current Magnet Compositions for Electric Cars

Existing literature on Ln separations for NdFeB magnet recycling consistently highlights the Nd/Dy pair as the most relevant for separation.^5,6,9,10,36^ However, the composition of modern NdFeB magnets is complex, and is continually evolving. These changes are not readily disclosed by manufacturers, nor have they been considered in scientific literature concerned with such Ln separations. Therefore, motors from two contemporary electric cars (one Chinese and one European, anonymized as A and B), were disassembled, and samples of the NdFeB magnets found in the rotor parts were taken for ICP-OES analysis.

Four magnets were collected from different positions (i.e., layers) within each of the two rotors. Lanthanides accounted for the expected ca. 30% mass in all cases, but the exact composition varied between both the car models and the rotor layers (Figure 8; Table S2). Magnets from model A contained no Pr, and only samples from one layer in the rotor contained any Dy. Magnets from model B contained both Nd and Pr, but lacked Dy altogether. All eight samples contained small amounts of Tb and—notably—significant quantities of Ho, which seemed to serve as a supplement or substitute for Dy and possibly Tb. There has been some recent academic interest in Ho-substitution in NdFeB magnets,^50,51^ but to our knowledge, this is the first time that Ho adoption is reported for a large industrial sector with such significance for recycling. In light of these findings, the oft-cited Pr/Nd/Tb/Dy quartet should be updated to include Ho for considerations of future NdFeB magnet recycling.

**Figure 8:**
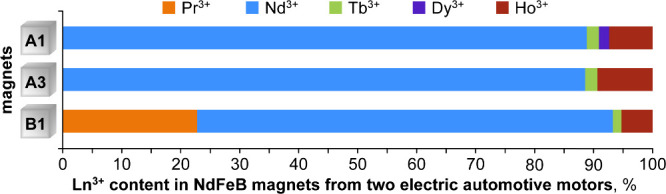
Lanthanides in electric car motor magnets. Representative results from ICP-OES analysis are shown as the % mass of the total Ln content. Samples A1 and A3 come from different layers of the same rotor; B1 is from a different car. Additional details are given in Table S2.

### Tuning Separations for Selected Ln Pairs

Taking into account the above results, the L^3^-Ln-acetate system was adapted for the relevant Ln pairs. In addition to the standard Nd/Dy pair, the Pr/Nd, Tb/Dy, and Tb/Ho pairs also needed to be considered. For each pair, the [Ln­(L^3^)] concentration, equivalents of acetate, and reaction time were tuned. As expected, the efficiency of the separations reflected the distances between the lanthanides—the greatest was seen for the distanced Nd/Dy pair, and the least for the adjacent Pr/Nd and Tb/Dy pairs (Figure 9). Separation factors from single-round precipitations were comparable to industrial SX methods, without any organic solvents (Tables S3–S6).^52,53^ Fuller optimization of this multiparametric system may offer further improvements, but was beyond the scope of this work.

**Figure 9:**
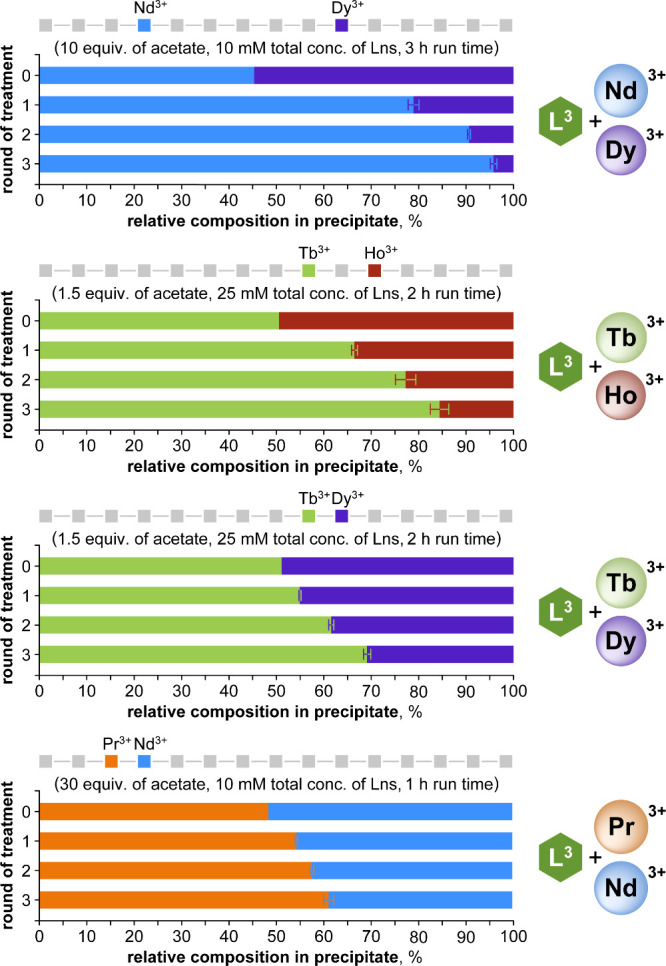
Repeated precipitations offer improved separations for selected Ln pairs with the L^3^-Ln-acetate system. Above each plot are indicated the positions of the Lns in the series, as well as their specific precipitation conditions. Plotted values represent the molar % of total Lns in the precipitate over three rounds of precipitation, starting with a ca. 1:1 ratio. Data are presented as averages and standard deviations of triplicates. Average single-step SF values were: 85.7 (Nd/Dy); 3.3 (Tb/Ho); 2.9 (Tb/Dy); 1.8 (Pr/Nd). Reaction pH was controlled by NaOH titration, rather than buffer; this change had no negative impact on the separations (Figure S22). Ln contents were determined by ICP-OES. The full data set and further details are given in Tables S3–S6.

Separations of adjacent lanthanides are often overlooked in the reports of new precipitation-based methods, which primarily emphasize separations of light and heavy lanthanides (e.g., Nd/Dy) by a single round of precipitation. For pairs of adjacent Lns (here, Pr/Nd and Tb/Dy), a single round of treatment yields only minor enrichment. Industrial SX methods remedy this by repeating the process until the desired purity of each phase is attained, but analogous procedures have typically not been considered for precipitation-based separations, where both the precipitate and the supernatant require reprocessing.

For the L^3^-Ln-acetate system, the precipitate phase was easily dissolved in 1 M HCl, and the separation was repeated by restoring the desired precipitation conditions (i.e., concentration, equivalents of acetate, and pH adjustment with NaOH). Consecutive repetitions increasingly enriched the precipitate in the less-soluble Ln, demonstrating that even the challenging adjacent Ln pairs could be separated with sufficient repetitions (Figure 9).

The Tb/Ho pair was found in a 1:5 ratio in the automotive magnets, and this presented a particular challenge for precipitation, as the less-soluble Tb was the minor component. However, very minor adjustments to the reaction conditions used for the equimolar Tb/Ho separations above enabled effective separations for this 1:5 ratio. Over three rounds of repeated separations, the precipitate was enriched to 29% Tb (from an initial 17%), while the supernatants were consistently rich in Ho (>95%) (Figure 10).

**Figure 10:**
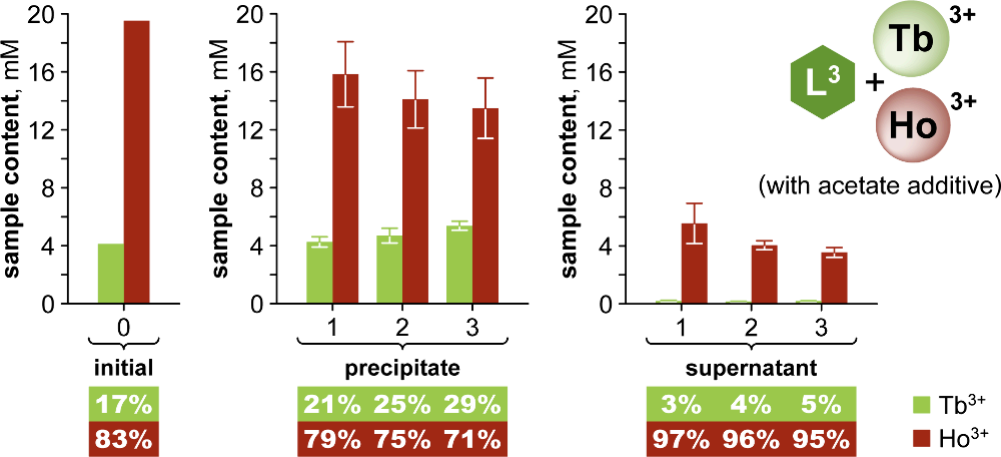
Repeated precipitations for Tb/Ho with the L^3^-Ln-acetate system, starting with a ca. 1:5 ratio. The reaction conditions for Tb/Ho separations from Figure 9 were here adjusted to 1 equiv of acetate and 5-h run times. The absolute Ln content expressed in mM relates to the same initial reaction volume for both the precipitate and supernatant. Relative % values are given below the plots. Lns were quantified by ICP-OES. Data are presented as averages and standard deviations of triplicates. The full data set and further details are given in Table S7.

During these reprecipitations, L^3^ maintains a stoichiometric ratio with Ln^3+^, and so requires no replenishment. It should also be noted that, for recycling applications, complete separation of the Lns may not be necessary (and may indeed be wasteful). In the interest of economy and efficiency, it would suffice to simply shift the ratio of Lns to what is required for the next application—especially when the intention is to make new magnets from the old.

While the repeated processing of the precipitate was relatively straightforward, achieving the same for the supernatant was considerably more complex. Further precipitation from solution was prevented by the presence of the solubilizing acetate; this acetate, therefore, needed to be removed. Due to significant differences in molecular size, the acetate (60 Da) could be separated from the chelates (∼650 Da) by ultrafiltration (UF) through a ∼ 250 Da cutoff membrane. However, as the acetate was removed, the chelates would precipitate and clog the membrane, hindering further progress. This was resolved by acidification of the solution to pH 3.5, at which the [Ln­(L^3^)] partially dissociates into solvated Ln^3+^ and free L^3^. Both species were still retained by the membrane, and that undesired, premature precipitation was thereby prevented (Figures S23 and S25). Then, after removing sufficient acetate and concentrating the solution, reaction conditions could be restored for another round of precipitation.

The final considerations in the full separation scheme were the isolation of the purified lanthanides, and the recovery of L^3^ for reuse. After the acid-assisted dissociation of [Ln­(L^3^)], the Lns were readily precipitated as their oxalates—a common approach in Ln hydrometallurgy.^10,54^ To recover pure L^3^ from the resulting solution, the excess oxalic acid was removed by the same UF method described above for the removal of acetate (Figures S24 and S26).

### Case Study: Magnet Processing

With all key steps established, the technology was tested by processing an automotive magnet sample (A3, Figure 8), as a case study (Figure 11). In short, the magnet was digested in 65% nitric acid, and the Lns were isolated as their oxalates by the addition of ammonium oxalate; the transition metals (i.e., Fe, Co, Ni) formed soluble coordination compounds under these conditions. The mixed-Ln oxalates were converted to their chlorides (Figure 11, **procedure 1**); this feedstock was then treated for separation by the L^3^-Ln-acetate system (**procedure 2**).

**Figure 11:**
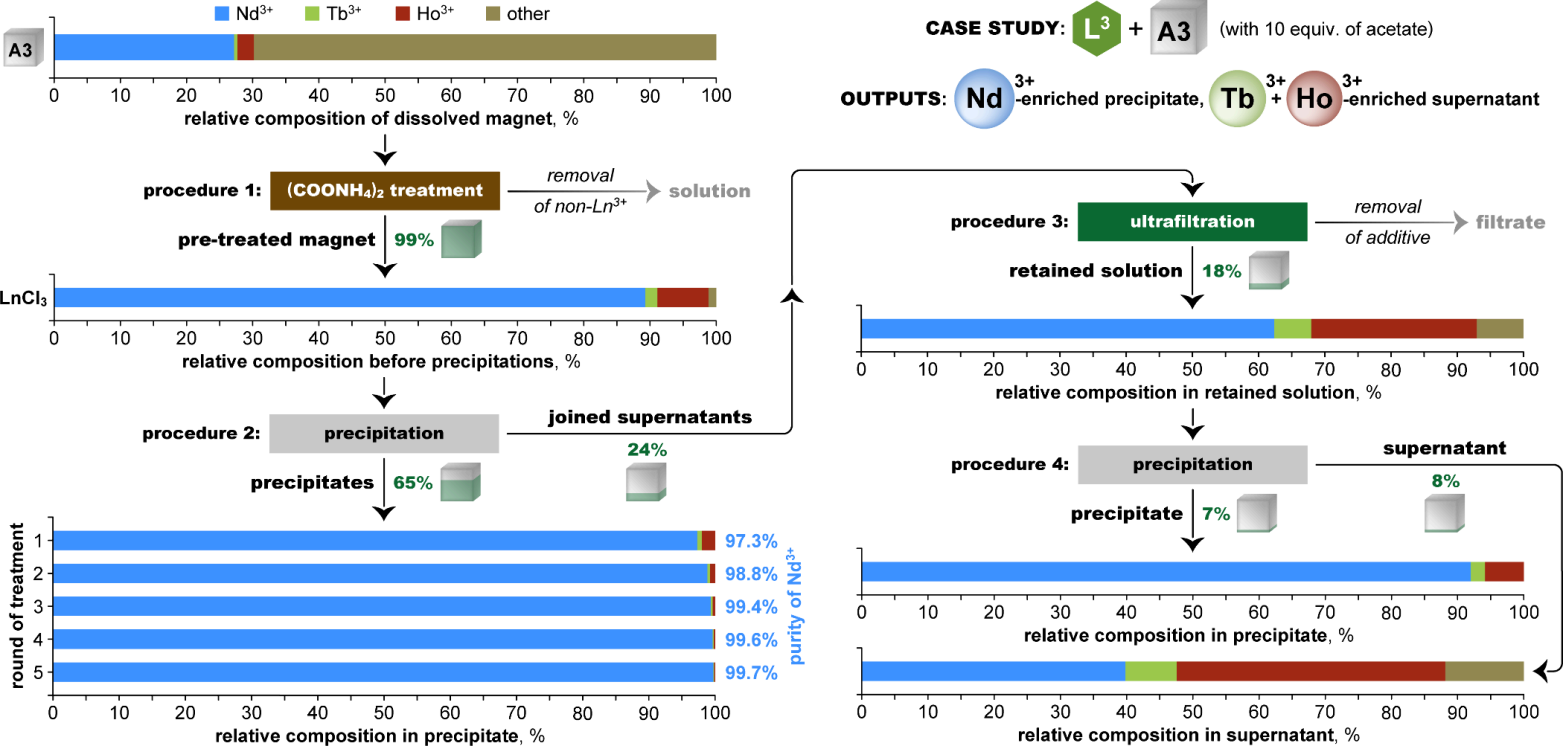
A case study processing of an NdFeB magnet from an electric car. Plots show the relative (mass %) compositions at each stage, as determined by ICP-OES, starting with a sample of the original magnet after digestion in HNO_3_ (top left, magnet A3 from Figure 8). The Nd, Tb, and Ho content is emphasized; all non-Ln elements (primarily Fe) are grouped as ‘other’. The partially filled green cubes represent the Ln recovery (molar %) at each stage relative to the total Nd+Tb+Ho content in the original magnet sample. **Procedure 1**: Lns were isolated from the other elements by treatment with ammonium oxalate to precipitate the Ln oxalates, then were converted to Ln chlorides. **Procedure 2**: The resulting mixed Lns were treated with the L^3^-Ln-acetate system (10 mM Ln^3+^, 10 equiv of acetate, 3-h run times). Repeated processing of the precipitate (dissolution in 1 M HCl followed by restoration of precipitation conditions) continuously improved the Nd enrichment of the precipitate, up to 99.7% after five rounds. **Procedure 3**: The joined supernatants were treated by UF to both remove the solubilizing acetate and concentrate the solution. **Procedure 4**: A final round of precipitation was performed on the UF-treated solution, further enriching the supernatant to 46% Ho and 8.8% Tb. Detailed procedures are given in the Supporting Information (page 5).

From the initial 90.4% Nd, the precipitate was enriched to 97.3% Nd content after one round of precipitation, and to 99.7% after five rounds, with 67% recovery of Nd. The supernatants were jointly processed by UF for acetate removal (**procedure 3**), and the retained, concentrated solution was then treated for one more round of precipitation (**procedure 4**). Although limitations of the UF setup resulted in a lower-than-ideal reaction concentration (and, likewise, a lesser-than-ideal degree of Nd precipitation), the supernatant was enriched to 46% Ho and 8.8% Tb, from the initial 7.8% and 1.9%, respectively.

The minor material losses incurred throughout the process are attributed to the small scale of the experiment: the samples taken for analysis, as well as any losses during transfers between reaction vessels, are quite noticeable here, though they would be negligible on a larger scale. Some further losses during UF treatment remain a point for future optimization, but are beyond the scope of this report.

## Conclusions

A new chemical system has been developed for lanthanide separations, with a special interest in NdFeB magnet recycling efforts. The system utilizes macrocyclic DO3A chelators with noncoordinating aromatic pendant arms—a chelator type not previously considered for Ln separations. For this class of chelators, selective precipitation of the light-Ln chelates from aqueous solution is likely driven by interchelate coordination bridging. Chelates of the smaller, heavier lanthanides cannot accommodate this bridging and remain soluble, thus enabling precipitation-based separations on the basis of Ln^3+^ size.

The selectivity for precipitation of specific lanthanides was tuned by the addition of coordinating additives which form ternary coordination compounds with the Ln chelates, thereby blocking the interchelate bridging and solubilizing the chelates. The cheap, environmentally benign acetate was identified as a highly effective additive for this system. By tuning the reaction concentrations, equivalents of acetate, and reaction durations, the same chemical constituents proved useful for separations of all Ln pairs relevant for the recycling of NdFeB magnets, from the benchmark Nd/Dy to the challenging Pr/Nd and Tb/Dy. Although a single round of precipitation may not achieve sufficient separations for the latter pairs, procedures were established to efficiently reprocess both the precipitate and supernatant phases, enabling the separations to be repeated as desired. The scheme was completed with a simple procedure for efficient recovery and reuse of the chelator.

The practical applicability of this system was demonstrated by processing an NdFeB magnet obtained from a contemporary electric car motor. The process was conducted under wholly aqueous conditions, avoiding the organic solvents common to current industrial practices. Furthermore, analysis of the magnets revealed a new trend in the automotive sector, where Ho is increasingly replacing Dy in NdFeB magnets. These findings altogether represent a significant step toward environmentally friendly and industrially scalable methods for the urban mining of lanthanides from neodymium magnets.

## Supplementary Material



## Data Availability

The raw data related to the solid-state NMR is freely accessible at 10.5281/zenodo.14988141.

## Notes

Materials and methods, synthetic procedures and characterization, NMR spectra of synthesized compounds, experimental details, crystallographic data, solid-state NMR data, TEM data, and extended data sets (PDF)
