# Supplementary material for: Macrocyclic Chelators for Aqueous Lanthanide Separations via Precipitation: Toward Sustainable Recycling of Rare-Earths from NdFeB Magnets
Source: J Am Chem Soc. 2025 Jun 19;147(26):22666–76. doi: 10.1021/jacs.5c04150 (PMC12232301; doi:10.1021/jacs.5c04150)
Supplement: Supplementary file 1 [file ja5c04150_si_001.pdf]

Supporting Information for

Macrocyclic Chelators for Aqueous Lanthanide Separations via Precipitation: Towards Sustainable Recycling of Rare-Earths from NdFeB Magnets

Kelsea G. Jones,[1,2] Tomáš David,[1] Martin Loula,[1] Stanislava Matějková,[1] Jan Blahut,[1] Anatolij Filimoněnko,[1] Miroslava Litecká,[3] Jan Rohlíček,[4] Jiří Böserle,[1] and Miloslav Polasek[1,5*]

[1] Institute of Organic Chemistry and Biochemistry, Czech Academy of Sciences, Flemingovo náměstí 542/2, 160 00, Prague 6, Czech Republic.

[2] Department of Inorganic Chemistry, Faculty of Science, Charles University, Hlavova 2030, 128 40, Prague 2, Czech Republic.

[3] Institute of Inorganic Chemistry, Czech Academy of Sciences, Husinec-Řež 1001, 250 68, Husinec-Řež, Czech Republic.

[4] Institut of Physics, Czech Academy of Sciences, Na Slovance 1999/2, 182 00, Prague 8, Czech Republic.

[5] Department of Applied Electronics, Faculty of Electrical Engineering and Computer Science, VSB—Technical University of Ostrava, 17. listopadu 2172/15, 708 00, Ostrava, Czech Republic.

[*] E-mail: **miloslav.polasek@uochb.cas.cz**

Table of Contents

Materials and Methods 3

Synthesis of chelators:

Figure S1. Synthesis and characterization of chelator **L**[1] 6
Figure S2. Synthesis and characterization of chelator **L**[2] 7
Figure S3. Synthesis and characterization of chelator **L**[3] 8
Figure S4. Large-scale synthesis of chelator **L**[3] 9
Figure S5. Synthesis and characterization of chelator **L**[4] 10
Figure S6. Synthesis and characterization of chelator **L**[5] 11
Figure S7. Synthesis and characterization of chelator **L**[6] 12
Figure S8. Synthesis and characterization of chelator **L**[7] 13
Figure S9. Synthesis and characterization of chelator **L**[8] 14

Initial observations and screenings:

Figure S10. Unexpected solubility pattern of [**Ln**(**L**^{1})] chelates 15
Figure S11. Chelators ineffective for Ln^{3+} separation 16
Figure S12. Kinetics of precipitation 17

Solid-state investigations:

Figure S13. TEM images of [**Nd**(**L**^{3})] and [**Dy**(**L**^{3})] precipitates 18
Figure S14. X-ray powder diffraction profile of the [**Nd**(**L**^{3})] chelate precipitate 19
Figure S15. $^{13}C^1\{H\}$ CP-MAS spectrum of [**Y**(**L**^{3})] chelate from precipitate 20
Figure S16. Refutation of the OH^- bridge hypothesis 21
Figure S17. Attempt to confirm carboxylate bridges using $^{13}C^1\{H\}$ CP-MAS 22
Figure S18. Synthesis and characterization of [**Dy**(**L**^{3})] chelate 23
Figure S19. Solid-state structure of [**Dy**(**L**^{3})] chelate 24
Figure S20. Isostructurality of [**Ln**(**L**^{3})] chelates 25

Precipitation and separation experiments:

Figure S21. Reproducibility of precipitation at solubility profile break in [**Ln**(**L**^{3})] series 26
Table S1. Effect of various additives on Nd^{3+}/Dy^{3+} separation in the [**Ln**(**L**^{3})] system 27
Table S2. Elemental analyses of real magnets 28
Figure S22. Buffer vs. titration pH control 29
Table S3. Optimized, repeated separations of Pr^{3+}/Nd^{3+} pair (1:1 initial ratio) 30
Table S4. Optimized, repeated separations of Nd^{3+}/Dy^{3+} pair (1:1 initial ratio) 31
Table S5. Optimized, repeated separations of Tb^{3+}/Dy^{3+} pair (1:1 initial ratio) 32
Table S6. Optimized, repeated separations of Tb^{3+}/Ho^{3+} pair (1:1 initial ratio) 33
Table S7. Optimized, repeated separations of Tb^{3+}/Ho^{3+} pair (1:5 initial ratio) 34
Figure S23. Removal of acetate additive by ultrafiltration 35
Figure S24. Removal of oxalic acid by ultrafiltration 36
Figure S25. Quantification of acetate additive removal by ultrafiltration 37
Figure S26. Quantification of of oxalic acid removal by ultrafiltration 38

References 39

Materials and Methods

General: Unless noted otherwise, reagents were purchased from commercial suppliers and were used directly. The macrocyclic starting material *1*,*4*,*7*-tris(tert-butoxycarbonylmethyl)-*1*,*4*,*7*,*10*-tetraazacyclododecane hydrobromide (tBuDO3A·HBr) was prepared by the alkylation of cyclen, according to published methods.[1] Throughout the text, H_2O means Milli-Q water (18.2 mΩ·cm). Ultrafiltration membranes were purchased from *Synder Filtration* (California, USA). Concentrations of all stock solutions of $LnCl_3$ (including YCl_3 and $ScCl_3$) were determined by ICP-OES.

Liquid chromatography: Analytical HPLC experiments were performed on 1260 Infinity II with UV (DAD, part number G7115A, 190 – 400 nm) and MS (single quadrupole, G6125B) detectors from *Agilent* (referred to as LC-MS or HPLC) equipped with a Luna Omega Polar C18 column (5 µm, 100 Å, 150 × 4.6 mm) using H_2O–MeCN gradients (1 mL min^{-1} flow rate) either with additives or without additives (specified where relevant). The UV-absorption profiles given for the characterization of the chelators and/or chelates were obtained from these chromatograms. Preparative HPLC experiments were performed on 1260 Infinity II (*Agilent*) equipped with YMC-Actus Triart C18 column (5 µm, 100 Å, 250 × 20.0 mm) using H_2O–MeCN gradients (20 mL min^{-1} flow rate) with either TFA (0.1%) or no additional additive.

High-resolution mass spectra: HRMS (with ESI ionization) were recorded on an *Agilent* 5975C MSD Quadrupole, Q-Tof micro from *Waters* or LTQ Orbitrap XL from *Thermo Fisher Scientific*.

Elemental analysis: CHN elemental analysis was performed on PE 2400 Series II CHN Analyzer from *Perkin Elmer*. Fluorine elemental analysis was performed by combustion of the sample in quartz vessel, followed by adsorption of HF in H_2O and determining its concentration by potentiometry using F^--selective electrode. Lanthanide content was determined by ICP-OES (SPECTRO Arcos Multiview from *SPECTRO Analytical Instruments*). All EA data are presented as: calcd. (found).

NMR spectroscopy: ^{1}H and ^{13}C NMR spectra were recorded on a Bruker Avance III™ HD 400 MHz spectrometer (401.0 MHz for ^{1}H, 100.6 MHz for ^{13}C) equipped with a broad-band Prodigy cryo-probe with ATM module (5 mm CPBBO BB-^{1}H/^{19}F/D Z-GRD) or on an Avance II™ 500 MHz (*Bruker*, 499.9 MHz for ^{1}H, 125.7 MHz for ^{13}C) spectrometer equipped with a 5 mm TBO probehead. The measurement temperature is indicated for the respective chelator. Chemical shifts are in ppm and coupling constants in Hz. Spectra were referenced using the residual DMSO-d_6 solvent signal (2.50 ppm in ^{1}H; 39.52 ppm in ^{13}C), or to the signal of *t*-BuOH external standard (1.25 ppm in ^{1}H; 32.43 ppm in ^{13}C) for spectra measured in D_2O. Integrals in showcased NMR spectra were rounded to integers for clarity; obscured signals that cannot be exactly integrated due to overlap with peak from solvent are coloured grey. Signals of the cyclen macrocycle are abbreviated *mc*.

X-ray powder diffraction: Data were acquired using the Debye-Scherrer transmission configuration on the powder diffractometer Smartlab (*Rigaku*) equipped with a Cu X-ray source (Cu/K_α radiation; λ = 1.5418 Å), focusing mirror (CBO-E), capillary holder and D/tex ultra 250 detector. The sample was ground, placed in a 0.5 mm borosilicate-glass capillary, and measured at 298±5 K over 18 h from 3° to 65° 2θ with 0.01° step size and with variable counting time.

Materials and Methods

Solid-state NMR spectroscopy: Solid-state NMR experiments were performed on a JEOL 600 MHz spectrometer at a 14.1 T field corresponding to a 600 MHz ^{1}H Larmor frequency, equipped with a 1.0 mm HX probe. The sample was packed in a 1.0 mm zirconia rotor and spun at 70 kHz under magic angle. The $^{13}C\{^{1}H\}$ spectra were acquired using 0.5 ms (from D_2O) and 2 ms (from H_2O) cross polarization from a ^{1}H spin-pool with pre-acquisition delay 1.5×T1(^{1}H) (obtained from saturation recovery experiment). The 35000 and 45000 scans were collected for [**Y**(**L**^{3})] precipitated from D_2O and H_2O, respectively. The ^{13}C spectrum of the Gd^{3+}-doped sample was acquired using direct ^{13}C excitation with spin-echo detection without ^{1}H decoupling and short pre-acquisition delay (1 s) and 120000 scans as recommended for paramagnetic systems.[2] The MAS ^{1}H of solid samples were acquired using rotor synchronized spin-echo detection. The spectrum of ^{2}H was acquired under static conditions using single-pulse experiment with direct FID detection. All raw data are accesible on https://doi.org/10.5281/zenodo.14988141.

X-Ray diffraction: Single-crystal data of all structures were collected on an XtaLAB Synergy S diffractometer (*Rigaku*) using Cu/K_α radiation (λ = 1.54184 Å, micro-focus X-ray source) and Hybrid Pixel Array Detector (HyPix-6000HE). A Cryostream 800 (*Oxford Cryosystems*) cooling device was used for data collection at 100 K. CrysAlis Pro software was used for data collection and cell refinement, data reduction and absorption correction (version 1.0.43, *Oxford Diffraction/ Agilent Technologies UK Ltd*, Yarnton, England, 2020). Data were corrected for absorption effects using empirical absorption correction (spherical harmonics), implemented in SCALE3 ABSPACK scaling algorithm and numerical absorption correction based on gaussian integration over a multifaceted crystal model. The structure was solved with the SHELXS structure solution program[3] using Direct Methods and refined with the SHELXL refinement package[4] using Least Squares minimisation implemented in Olex2[5]. Anisotropic displacement parameters were refined for all non-H atoms. Hydrogen atoms were placed in idealized positions, and refined using riding models and rotating model for coodinated water molecule. Selected crystallographic parameters are given in Figure S19. Crystallographic data for structural analysis have been deposited with the Cambridge Crystallographic Data Centre (CCDC no. 2419472). Copies of this information may be obtained free of charge from http://www.ccdc.cam.ac.uk.

TEM: The TEM grids with [**Ln**(**L**^{3})] precipitates were loaded into a 200 kV Jeol 2100 Plus electron microscope equipped with a TVIPS TemCam-XF416 CMOS camera. EM data were acquired using Serial EM software[6] at a nominal magnification of ×15000 (7.701 Å pixel size; −30 µm defocus) or ×60000 (1.939 Å pixel size; −0.5 µm defocus).

Materials and Methods

Processing of a sample NdFeB magnet: Automotive magnet sample A3, previously digested by HNO_3 and analyzed by ICP-OES (Table S2) was here treated to demonstrate separations of the Lns.

1: Isolation of Lns. To isolate the Lns from the numerous other elements present in the sample, the Lns were precipitated as their oxalates. 830 µL of the dissolved solution of A3 (ca. 2.0 M total dissolved metal content) was added to an aqueous solution of $(COONH_4)_2$ (10 mL of 0.5 M; ca. 3.0 equiv. for the total dissolved metal content); this was stirred at RT for 1 h to precipitate the Ln oxalates as an off-white powder. The precipitate and supernatant were separated by centrifugation, the bright green solution was pipetted away, and the precipitate was rinsed with H_2O three times. The Ln oxalate precipitate was dissolved by acidification with concentrated nitric acid (1.5 mL). This nitric acid solution was slowly added to a solution of 2.0 M aq. NaOH (15 mL) to precipitate the mixed $Ln(OH)_3$. Again, the precipitate was isolated by centrifugation and the extraneous solution was removed by pipette. The $Ln(OH)_3$ readily dissolved in 1.0 M aq. HCl, providing an $LnCl_3$ stock solution for further treatment.

2: [Ln(L^3)]-acetate separations & precipitate reprocessing. A-10 mL reaction was prepared in a 20-mL scintillation vial, containing 10 mM Ln^{3+}, 11 mM $\mathbf{L^3}$, and 100 mM AcOH. The reaction was titrated to pH 6.1 by addition of aq. NaOH, and then was stirred at RT for 3 h before being worked up. An aliquot of the suspension was taken for analysis; the aliquot was centrifuged to separate the supernatant and precipitate, and both phases were treated with dilute aq. HCl. The remaining bulk reaction suspension was centrifuged to isolate the precipitate. The bulk supernatant was removed, acidified by the addition of 1 mL of 1.0 M aq. HCl, and reserved. The bulk precipitate was reprocessed in the same manner described for the repeated separations of selected Ln pairs (i.e., by dissolving the precipitate in dilute HCl and re-initiating the precipitation process by the addition of 10 equiv. of AcOH and NaOH titration; Tables S4–S7). This precipitation process was repeated for a total of five rounds of separation, and the supernatants from each round were reserved.

3: Supernatant reprocessing. After the fifth round of precipitation, the reserved supernatants were joined and treated by ultrafiltration to remove the acetate additive and facilitate the reprocessing of this portion of the material. The ca. 40 mL of joined solutions were first filtered through a 0.45 µm RC syringe microfilter. The pH of the solution was lowered to 3.1 by the addition of 1.0 M aq. HCl. This solution was then treated by ultrafiltration through an NFS membrane using ca. 4 bar of N_2 to pressurize the system. When the volume of the solution had been reduced to about 3 mL, an additional 6 mL of H_2O were added, and ultrafiltration treatment was continued. This was repeated thrice more, for a total of 4 additions of 6 mL of H_2O. The volume of the solution was finally reduced to 2.5 mL, and ultrafiltration was stopped. An aliquot of the ultrafiltration filtrate (0.1 mL) was reserved for analysis, and the 2.4 mL of the solution containing Ln^{3+} and $\mathbf{L^3}$ was treated for a sixth round of precipitation. The concentration of [**Ln(L^3)**] remaining in this solution was approximated by HPLC-DAD analysis, in order to determine the quantity of AcOH to be added to the solution. After NaOH titration to pH 6, the reaction was stirred at RT for 3 h. The reaction suspension was then centrifuged, and the supernatant and precipitate were both treated with dilute HCl and analyzed by ICP-OES for Ln content determination.

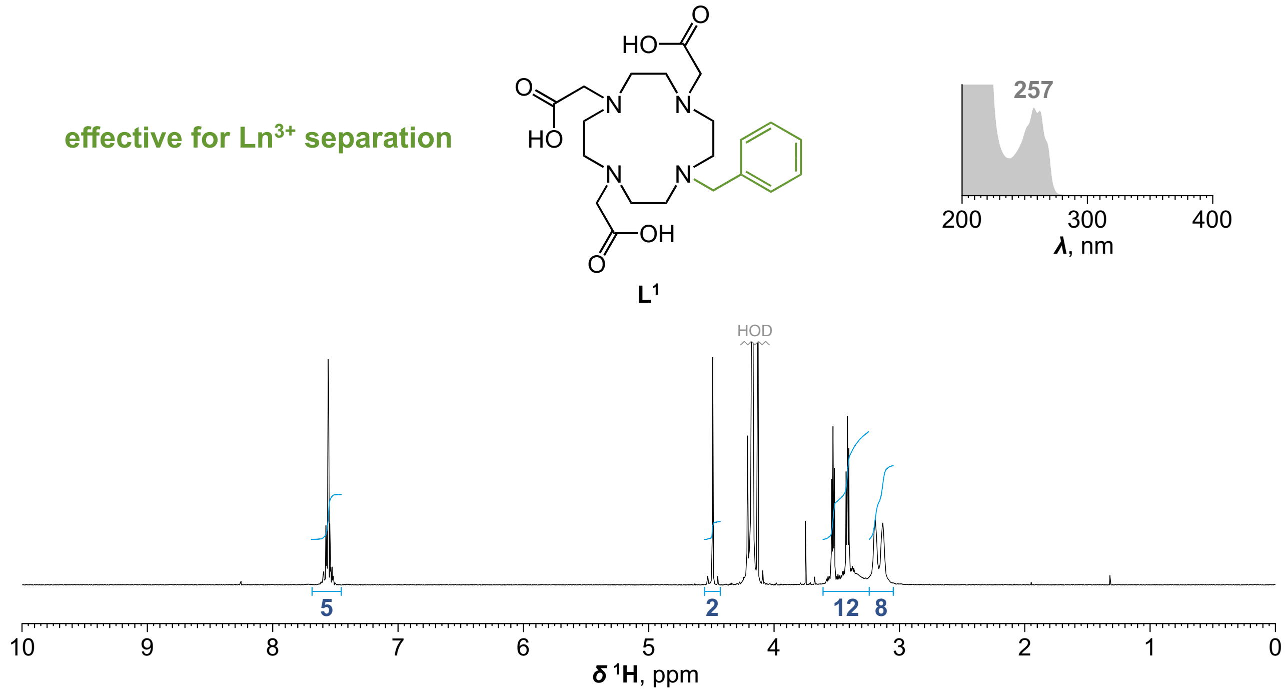

Figure S1. Synthesis and characterization of chelator L[1]. To a solution of tBuDO3A·HBr (1.0 g; 1.68 mmol; 1.0 equiv.) in MeCN (50 mL) was added dried K_2CO_3 (1.16 g; 8.39 mmol; 5.0 equiv.) and neat benzyl bromide (302 mg; 1.76 mmol; 1.1 equiv.); the reaction was stirred at RT for 3 d. Solids were filtered off and the filtrate was concentrated by rotary evaporation. The crude product was purified by reversed-phase preparative HPLC (MeCN/H_2O gradient with 0.1% TFA). Fractions were collected and rotary evaporated to dryness to yield the alkylated intermediate as a colorless oil. This intermediate was dissolved in TFA (10 mL) and stirred at RT for 16 h. The acid was removed by rotary evaporation and the crude product was purified by reversed-phase preparative HPLC (MeCN/H_2O gradient with 0.1% TFA). Fractions were collected and concentrated by rotary evaporation. The product was lyophilized to yield **L[1]** as a fluffy white solid. **Yield:** 304 mg (26%; 2 steps; based on tBuDO3A·HBr). **NMR** (D_2O, T = 368 K): **^{1}H** (499.9 MHz) δ_H 3.07–3.24 (*mc*, bm, 8H); 3.25–3.61 (*mc*, C*H*$_2$–COOH, bm, 12H); 4.13 (C*H*$_2$–COOH, s, 2H); 4.49 (C*H*$_2$–arom., s, 2H); 7.43–7.69 (*arom.*, m, 5H). **^{13}C{^{1}H}** (125.7 MHz) δ_C 49.2 (*mc*, s); 49.3 (*mc*, s); 50.8 (*mc*, s); 52.5 (*mc*, s); 54.1 (*C*H$_2$–COOH, s); 55.6 (*C*H$_2$–COOH, s); 59.1 (*C*H$_2$–arom., s); 130.0 (*arom.*, s); 130.5 (*arom.*, s); 131.2 (*arom.*, s); 131.6 (*arom.*, s); 169.3 (*C*O, s); 173.7 (*C*O, s). **ESI-HRMS:** 437.2392 $[M+H]^+$ (theor. $[C_{21}H_{33}N_4O_6]^+$ = 437.2395). **UV absorption:** λ_{max} = 257 nm. **EA** ($C_{21}H_{32}N_4O_6$·2.1TFA·1.1H_2O, M_R = 695.7): C 43.5 (43.6); H 5.3 (5.0); N 8.1 (7.8); F 17.2 (17.3).

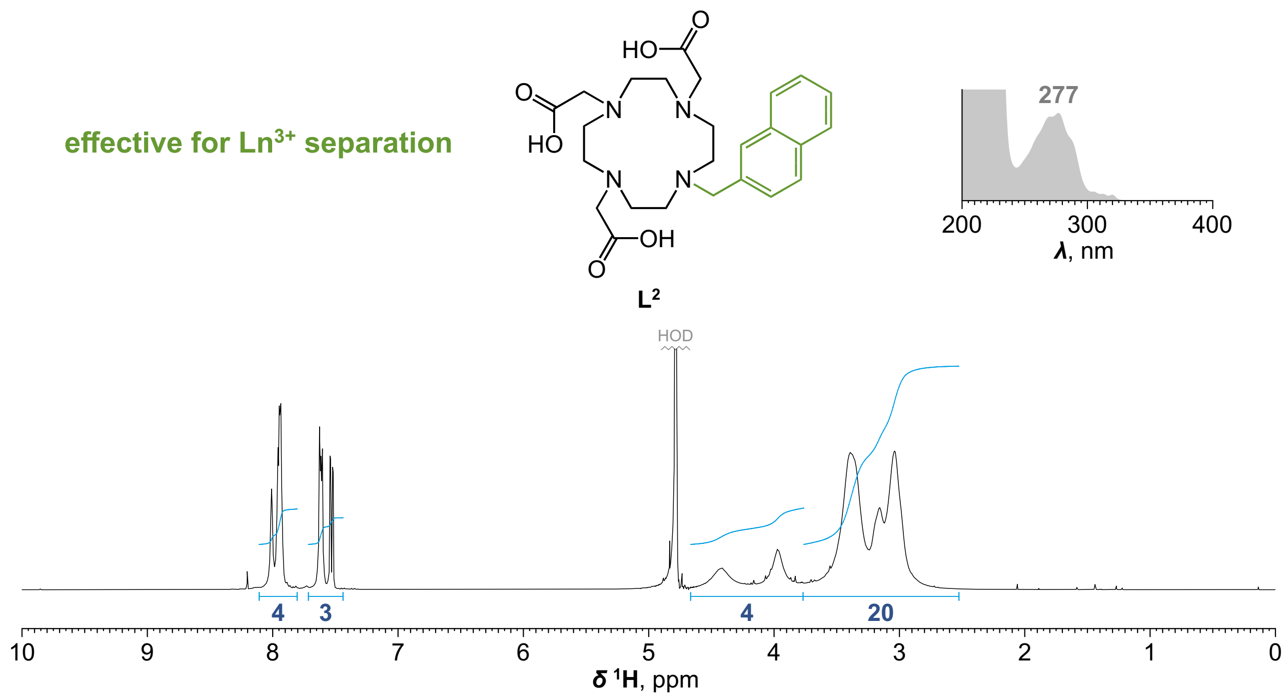

Figure S2. Synthesis and characterization of chelator $\mathbf{L^2}$. To a solution of tBuDO3A·HBr (250 mg; 0.42 mmol; 1.0 equiv.) in MeCN (13 mL) was added dried K_2CO_3 (290 mg; 2.10 mmol; 5.0 equiv.) and a solution of *2*-(bromomethyl)naphthalene (97.4 mg; 0.44 mmol; 1.0 equiv.) in MeCN (2 mL); the reaction was stirred at RT for 16 h. Solids were filtered off and the filtrate was concentrated by rotary evaporation. The crude product was purified by reversed-phase preparative HPLC (MeCN/H_2O gradient with 0.1% TFA). Fractions were collected and rotary evaporated to dryness to yield the alkylated intermediate as a colorless oil. This intermediate was dissolved in TFA (5 mL) and stirred at RT for 16 h. The acid was removed by rotary evaporation and the crude product was purified by reversed-phase preparative HPLC (MeCN/H_2O gradient with 0.1% TFA). Fractions were collected and concentrated by rotary evaporation. The product was lyophilized to yield $\mathbf{L^2}$ as a fluffy white solid. **Yield:** 181 mg (61%; 2 steps; based on tBuDO3A·HBr). **NMR** (D_2O, T = 298 K): $\mathbf{^1H}$ (401.0 MHz) δ_H 2.50–3.75 (*mc*, C*H*₂–CO, bm, 16+4H); 3.97 (C*H*₂–CO, bs, 2H); 4.42 (C*H*₂–arom., bs, 2H); 7.53 (*arom.*, dd, 1H, $^3J_{HH}$ = 9, $^4J_{HH}$ = 2); 7.57–7.68 (*arom.*, m, 2H); 7.86–8.10 (*arom.*, m, 4H). $\mathbf{^{13}C\{^1H\}}$ (100.6 MHz) δ_C 45.1–53.6 (4×*mc*, C*H*₂–CO, bm); 54.9 (*C*H₂–CO, s); 57.6 (*C*H₂–arom., s); 127.0 (*arom.*, s); 127.2 (*arom.*, s); 127.6 (*arom.*, s); 127.7 (*arom.*, s); 128.3 (*arom.*, s); 129.7 (*arom.*, s); 130.9 (*arom.*, bs); 132.8 (*arom.*, s); 133.3 (*arom.*, s); 133.4 (*arom.*, s); 169.0 (*C*O, s); 173.2 (*C*O, s). **ESI-HRMS:** 487.2549 $[M+H]^+$ (theor. $[C_{25}H_{35}N_4O_6]^+$ = 487.2551). **UV absorption:** λ_{max} = 277 nm. **EA** ($C_{25}H_{34}N_4O_6$·1.8TFA·0.7H_2O, M_R = 704.4): C 49.7 (50.0); H 5.5 (5.1); N 7.6 (7.3); F 13.9 (13.5).

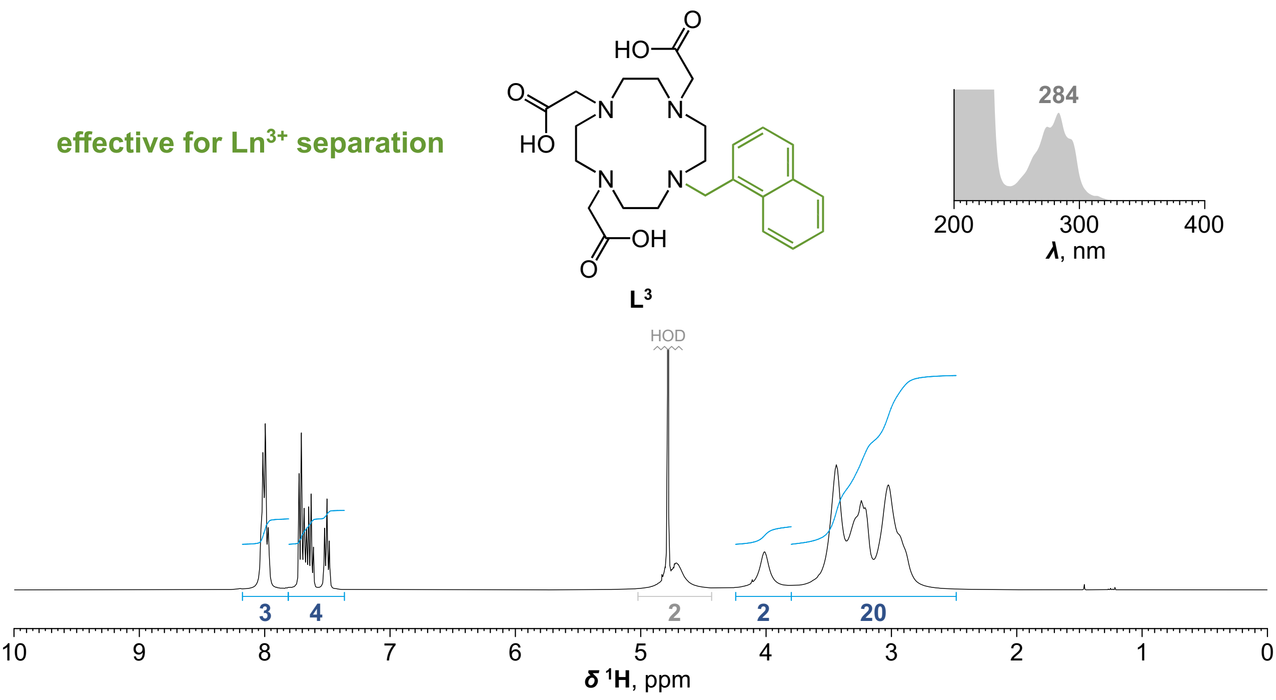

Figure S3. Synthesis and characterization of chelator L³. To a solution of tBuDO3A·HBr (250 mg; 0.42 mmol; 1.0 equiv.) in MeCN (13 mL) was added dried K_2CO_3 (290 mg; 2.10 mmol; 5.0 equiv.) and a solution of *1*-(bromomethyl)naphthalene (97.4 mg; 0.44 mmol; 1.0 equiv.) in MeCN (2 mL); the reaction was stirred at RT for 16 h. Solids were filtered off and the filtrate was concentrated by rotary evaporation. The crude product was purified by reversed-phase preparative HPLC (MeCN/H_2O gradient with 0.1% TFA). Fractions were collected and rotary evaporated to dryness to yield the alkylated intermediate as a colorless oil. This intermediate was dissolved in TFA (5 mL) and stirred at RT for 16 h. The acid was removed by rotary evaporation and the crude product was purified by reversed-phase preparative HPLC (MeCN/H_2O gradient with 0.1% TFA). Fractions were collected and concentrated by rotary evaporation. The product was lyophilized to yield **L³** as a fluffy white solid. **Yield:** 202 mg (68%; 2 steps; based on tBuDO3A·HBr). **NMR** (D_2O, T = 298 K): **^{1}H** (401.0 MHz) δ_H 2.48–3.77 (*mc*, C*H*$_2$–CO, bm, 16+4H); 4.01 (C*H*$_2$–CO, bs, 2H); 4.78 (C*H*$_2$–arom., bs, 2H); 7.51 (*arom.*, t, 1H, $^3J_{HH}$ = 8); 7.57–7.77 (*arom.*, m, 3H); 7.91–8.10 (*arom.*, m, 3H). **^{13}C{^{1}H}** (100.6 MHz) δ_C 46.7–53.1 (4×*mc*, *C*H$_2$–CO, bm); 53.4 (*C*H$_2$–CO, s); 54.8 (*C*H$_2$–arom., s); 122.0 (*arom.*, s); 125.2 (*arom.*, bs); 126.0 (*arom.*, s); 127.0 (*arom.*, s); 128.2 (*arom.*, s); 129.4 (*arom.*, s); 130.2 (*arom.*, s); 131.1 (*arom.*, bs); 131.6 (*arom.*, s); 133.6 (*arom.*, s); 168.7 (*C*O, s); 173.0 (*C*O, s). **ESI-HRMS:** 487.2553 $[M+H]^+$ (theor. $[C_{25}H_{35}N_4O_6]^+$ = 487.2551). **UV absorption:** λ_{max} = 284 nm. **EA** ($C_{25}H_{34}N_4O_6$·1.8TFA·1.3H_2O, M_R = 715.2): C 48.3 (48.2); H 5.5 (5.2); N 7.9 (8.0); F 14.1 (14.0).

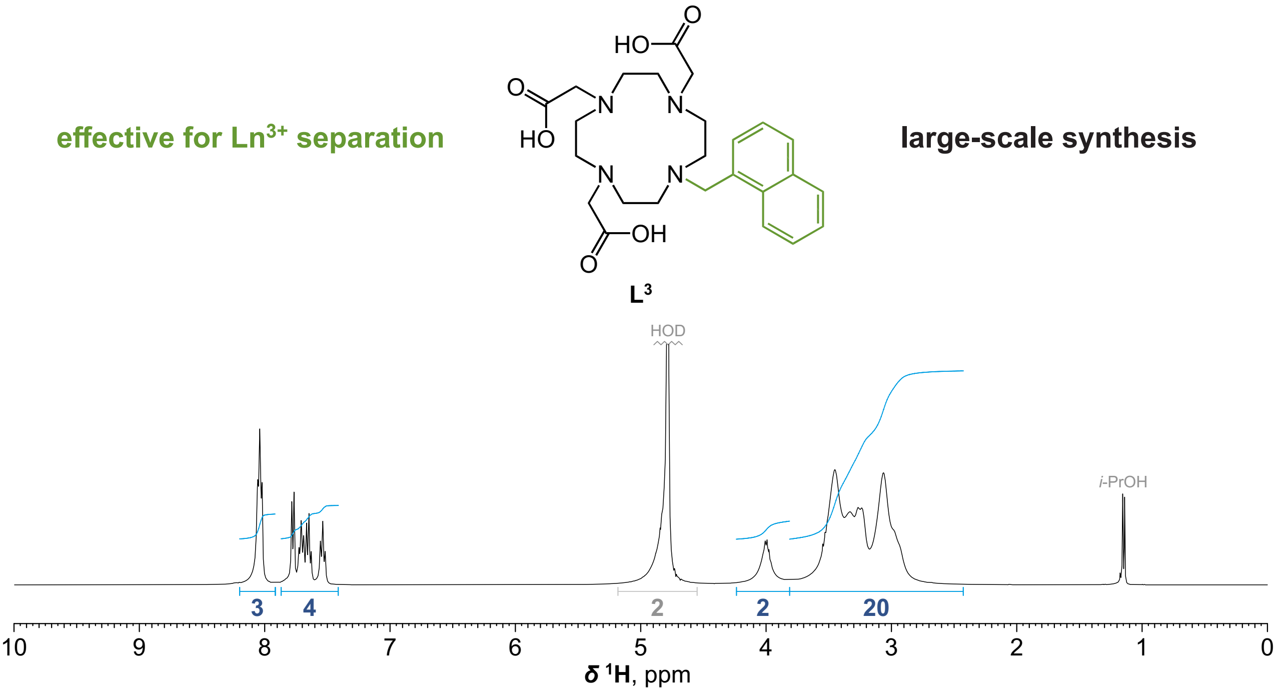

Figure S4. Large-scale synthesis of chelator L3. In a round-bottom glass flask (500 mL), tBuDO3A·HBr (13.00 g; 21.83 mmol; 1.00 equiv.) was dissolved in MeCN (HPLC grade; 175 mL) followed by addition of K_2CO_3 (not dried; 12.08 g; 87.41 mmol; 4.00 equiv.) and a solution of *1*-(bromomethyl)naphthalene (4.925 g; 22.28 mmol; 1.02 equiv.) in MeCN (HPLC grade; 75 mL). The resulting suspension was stirred for 16 h at RT. Solids were filtered off using glass frit (S3; solid was further washed with MeCN) and the filtrate was evaporated to dryness. The residue was dissolved in a mixture of DCM (250 mL) and dil. aq. $NaHCO_3$ (250 mL) and transferred to a separatory funnel. After brief shaking, the organic layer was separated and the aqueous layer further extracted by DCM (3 × 100 mL). Combined organic layers were dried using anh. Na_2SO_4 and filtered. The filtrate was evaporated to dryness and the residual oil briefly dried under high vacuum. The residue (14.9 g) was carefully dissolved in neat TFA (100 mL; 1.31 mol; ≥60 equiv.) and the resulting clear solution was stirred at RT for 12 h. After that, another portion of TFA (50 mL; 654 mmol; ≥30 equiv.) was added and the mixture was further stirred at RT for 1 h. The reaction mixture was then evaporated to dryness. Residual orange oil was dissolved in 3.0 M HCl (72 mL; 216 mmol; ≥10 equiv.) with the help of heating followed by addition of *i*-PrOH (200 mL) and EtOH (96%; 600 mL). The resulting precipitate was collected on glass frit (S2), air dried and further dried under high vacuum overnight. The precipitate (11.48 g; 21.76 mmol – if assuming M·2HCl; 1.0 equiv.) was re-dissolved in 1.0 M HCl (88 mL; 88 mmol; ~4 equiv.) followed by addition of *i*-PrOH (200 mL) and Et_2O (800 mL). The resulting precipitate was collected on glass frit (S2), briefly washed with Et_2O, air dried and further briefly dried under high vacuum. The resulting solid was then mechanically crushed and dried under high vacuum overnight to yield the hydrochloride salt of **L3** as a white solid. **Yield:** 10.98 g (86%; 2 steps; based on tBuDO3A·HBr). **NMR** (D_2O, T = 298 K, contains 10 molar% of residual *i*-PrOH): **^{1}H** (401.0 MHz) δ_H 2.42–3.81 (*mc*, CH_2–CO, bm, 16+4H); 4.01 (CH_2–CO, bs, 2H); ~4.8 (CH_2–arom., bs, 2H, obscured by HOD signal); 7.54 (*arom.*, t, 1H, $^3J_{HH}$ = 8); 7.60–7.82 (*arom.*, m, 3H); 7.92–8.19 (*arom.*, m, 3H). **EA** ($C_{25}H_{34}N_4O_4$·2.0HCl·3.0H_2O·0.1*i*-PrOH, M_R = 587.5): C 51.7 (51.8); H 7.3 (6.5); N 9.5 (9.2); Cl 12.1 (11.8).

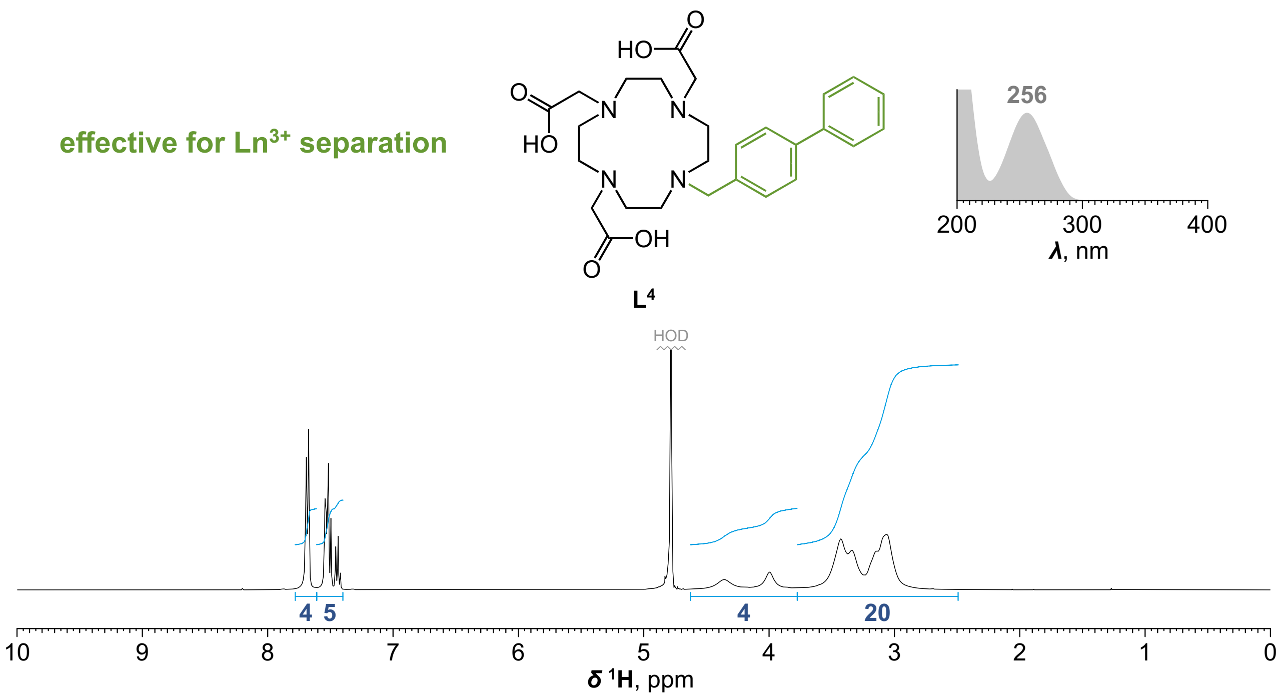

Figure S5. Synthesis and characterization of chelator L^4. To a solution of tBuDO3A·HBr (300 mg; 0.50 mmol; 1.0 equiv.) in MeCN (15 mL) was added dried K_2CO_3 (348 mg; 2.52 mmol; 5.0 equiv.) and neat *1*-(bromomethyl)-*4*-phenylbenzene (131 mg; 0.53 mmol; 1.1 equiv.); the reaction was stirred at RT for 16 h. Solids were filtered off and the filtrate was concentrated by rotary evaporation. The crude product was purified by reversed-phase preparative HPLC (MeCN/H_2O gradient with 0.1% TFA). Fractions were collected and rotary evaporated to dryness to yield the alkylated intermediate as a colourless oil. This intermediate was dissolved in TFA (5 mL) and stirred at RT for 16 h. The acid was removed by rotary evaporation and the crude product was purified by reversed-phase preparative HPLC (MeCN/H_2O gradient with 0.1% TFA). Fractions were collected and concentrated by rotary evaporation. The product was lyophilized to yield **L^4** as a fluffy white solid. **Yield:** 285 mg (76%; 2 steps; based on tBuDO3A·HBr). **NMR** (D_2O, T = 298 K): **^{1}H** (401.0 MHz) δ_H 2.46–3.77 (*mc*, C*H*$_2$–CO, bm, 16+4H); 4.00 (C*H*$_2$–CO, bs, 2H); 4.36 (C*H*$_2$–arom., bs, 2H); 7.38–7.61 (*arom.*, m, 5H); 7.61–7.79 (*arom.*, m, 4H). **^{13}C{^{1}H}** (100.8 MHz) δ_C 46.6–53.7 (4×*mc*, C*H*$_2$–CO, bm); 55.0 (*C*H$_2$–CO, s); 57.3 (*C*H$_2$–arom., s); 126.9 (*arom.*, s); 127.9 (*arom.*, s); 128.2 (*arom.*, s); 129.2 (*arom.*, s); 131.1 (*arom.*, s); 131.2 (*arom.*, s); 139.3 (*arom.*, s); 141.9 (*arom.*, bs); 169.0 (*C*O, s); 173.2 (*C*O, s). **ESI-HRMS**: 513.2710 $[M+H]^+$ (theor. $[C_{27}H_{37}N_4O_6]^+$ = 513.2708). **UV absorption:** λ_{max} = 256 nm. **EA** ($C_{27}H_{36}N_4O_6$·1.8TFA·1.3H_2O, M_R = 741.2): C 49.7 (50.0); H 5.5 (5.1); N 7.6 (7.3); F 13.9 (13.5).

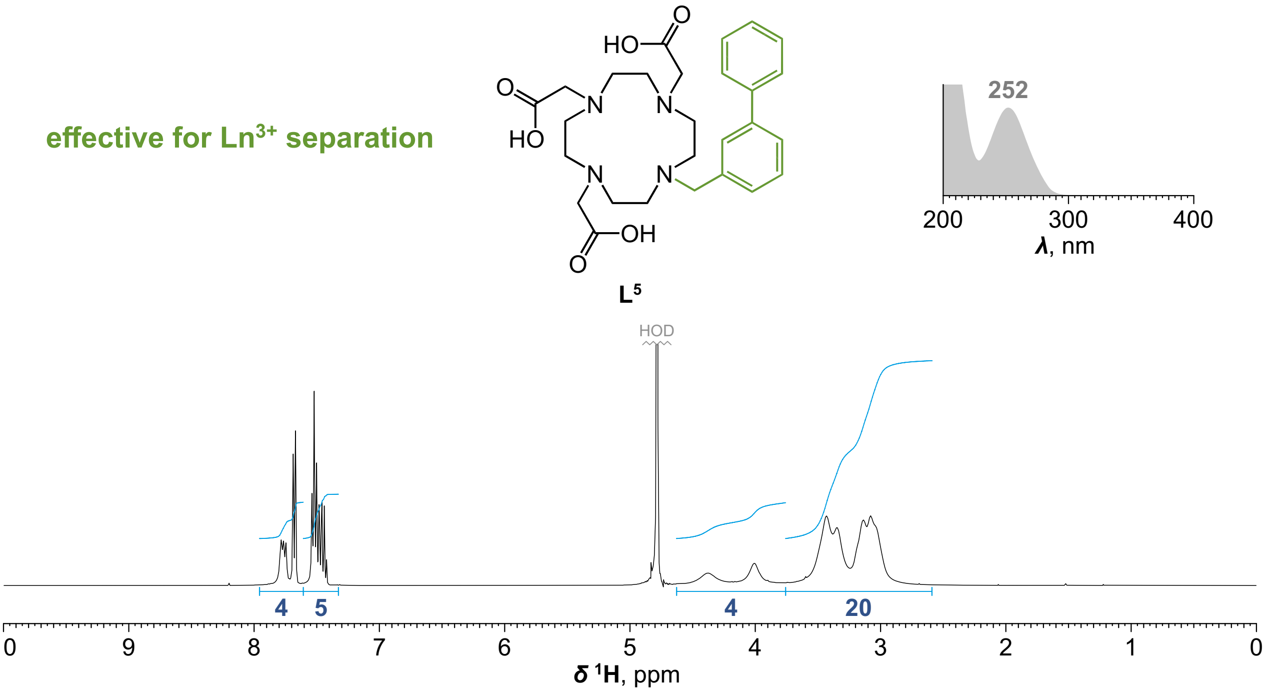

Figure S6. Synthesis and characterization of chelator L^5. To a solution of tBuDO3A·HBr (250 mg; 0.42 mmol; 1.0 equiv.) in MeCN (15 mL) was added dried K_2CO_3 (290 mg; 2.10 mmol; 5.0 equiv.) and neat *1*-(bromomethyl)-*3*-phenylbenzene (109 mg; 0.44 mmol; 1.0 equiv.); the reaction was stirred at RT for 16 h. Solids were filtered off and the filtrate was concentrated by rotary evaporation. The crude product was purified by reversed-phase preparative HPLC (MeCN/H_2O gradient with 0.1% TFA). Fractions were collected and rotary evaporated to dryness to yield the alkylated intermediate as a white powder. This intermediate was dissolved in TFA (5 mL) and stirred at RT for 16 h. The acid was removed by rotary evaporation and the crude product was purified by reversed-phase preparative HPLC (MeCN/H_2O gradient with 0.1% TFA). Fractions were collected and concentrated by rotary evaporation. The product was lyophilized to yield **L^5** as a fluffy white solid. **Yield:** 227 mg (73%; 2 steps; based on tBuDO3A·HBr). **NMR** (D_2O, T = 298 K): **^{1}H** (401.0 MHz) δ_H 2.64–3.75 (*mc*, C*H*$_2$–CO, bm, 16+4H); 4.01 (C*H*$_2$–CO, bs, 2H); 4.38 (C*H*$_2$–arom., bs, 2H); 7.38–7.58 (*arom.*, m, 5H); 7.63–7.83 (*arom.*, m, 4H). **^{13}C{^{1}H}** (100.8 MHz) δ_C 46.9–53.2 (4×*mc*, C*H*$_2$–CO, bm); 54.8 (*C*H$_2$–CO, s); 57.8 (*C*H$_2$–arom., s); 126.9 (*arom.*, s); 128.2 (*arom.*, s); 129.1 (*arom.*, s); 129.2 (*arom.*, s); 129.7 (*arom.*, s); 130.4 (*arom.*, s); 139.2 (*arom.*, s); 141.5 (*arom.*, s); 169.0 (*C*O, s); 173.3 (*C*O, s). **ESI-HRMS**: 513.2711 $[M+H]^+$ (theor. $[C_{27}H_{37}N_4O_6]^+$ = 513.2708). **UV absorption:** λ_{max} = 252 nm. **EA** ($C_{27}H_{36}N_4O_6$·1.8TFA·1.8H_2O, M_R = 750.2): C 49.2 (49.3); H 5.6 (5.2); N 7.5 (7.3); F 13.4 (13.8).

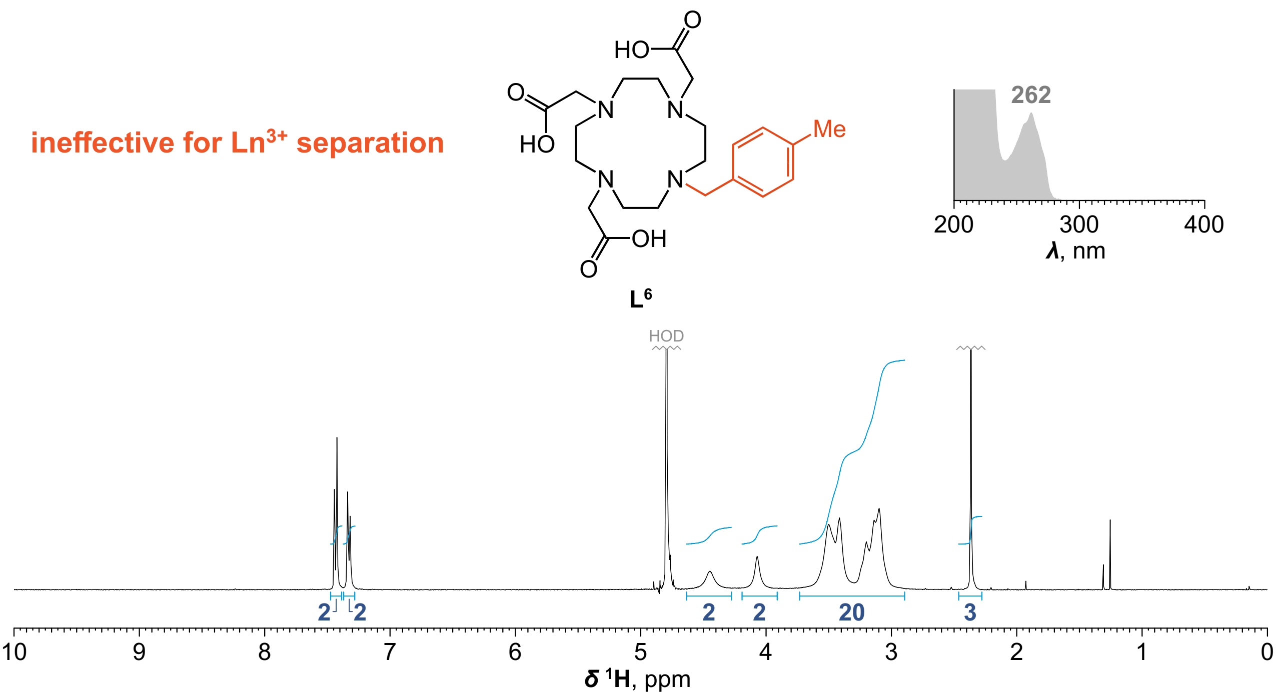

Figure S7. Synthesis and characterization of chelator L^6. To a solution of tBuDO3A·HBr (400 mg; 0.67 mmol; 1.0 equiv.) in MeCN (20 mL) was added dried K_2CO_3 (464 mg; 3.36 mmol; 5.0 equiv.) and a solution of *1*-(chloromethyl)-*4*-methylbenzene (99 mg; 0.71 mmol; 1.1 equiv.) in MeCN (4 mL); the reaction was stirred at RT for 16 h. Solids were filtered off and the filtrate was concentrated by rotary evaporation. The crude product was purified by reversed-phase preparative HPLC (MeCN/H_2O gradient with 0.1% TFA). Fractions were collected and rotary evaporated to dryness to yield the alkylated intermediate as a colorless oil. This intermediate was dissolved in TFA (5 mL) and stirred at RT for 16 h. The acid was removed by rotary evaporation and the crude product was purified by reversed-phase preparative HPLC (MeCN/H_2O gradient with 0.1% TFA). Fractions were collected and concentrated by rotary evaporation. The product was lyophilized to yield $\mathbf{L^6}$ as a fluffy white solid. **Yield:** 257 mg (54%; 2 steps; based on tBuDO3A·HBr). **NMR** (D_2O, T = 298 K): ^{1}**H** (401.0 MHz) δ_H 2.37 (CH_3, s, 3H); 2.89–3.73 (*mc*, CH_2–COOH, m, 16+4H); 4.07 (CH_2–COOH, bs, 4H); 4.45 (CH_2–arom, bs, 2H); 7.33 (*arom.*, d, 2H, $^3J_{HH}$ = 8); 7.44 (*arom.*, d, 2H, $^3J_{HH}$ = 8). ^{13}**C{**^{1}**H}** (100.6 MHz) δ_C 20.3 (CH_3, s); 48.0 (2×*mc*, bm); 49.5 (*mc*, s); 51.3 (*mc*, s); 53.0 (CH_2–COOH, s); 55.1 (CH_2–COOH, s); 57.4 (CH_2–arom, s); 130.4 (*arom.*, s); 130.7 (*arom.*, s); 141.0 (2×*arom.*, s); 173.1 (*C*O, s); 173.3 (*C*O, s). **ESI-HRMS**: 449.2400 $[M-H]^+$ (theor. $[C_{22}H_{33}N_4O_6]^-$ = 449.2406). **UV absorption:** λ_{max} = 262 nm. **EA** ($C_{22}H_{34}N_4O_6$·2.1TFA·0.9H_2O, M_R = 706.1): C 44.6 (44.9); H 5.4 (5.3); N 7.9 (8.1); F 16.9 (16.9).

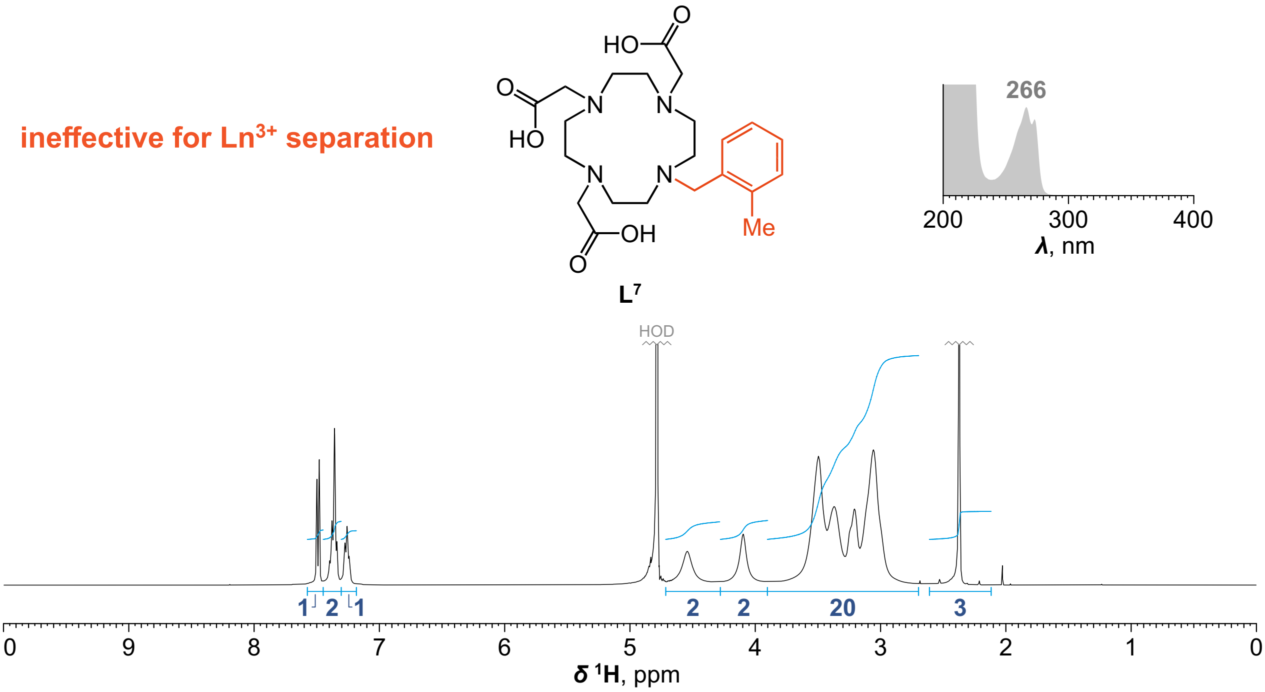

Figure S8. Synthesis and characterization of chelator L^7. To a solution of tBuDO3A·HBr (400 mg; 0.67 mmol; 1.0 equiv.) in MeCN (20 mL) was added dried K_2CO_3 (371 mg; 2.69 mmol; 4.0 equiv.) and a solution of *1*-(bromomethyl)-*2*-methylbenzene (140 mg; 0.76 mmol; 1.1 equiv.) in MeCN (2 mL); the reaction was stirred at RT for 16 h. Solids were filtered off and the filtrate was concentrated by rotary evaporation. The crude product was purified by reversed-phase preparative HPLC (MeCN/H_2O gradient with 0.1% TFA). Fractions were collected and rotary evaporated to dryness to yield the alkylated intermediate as a colorless oil. This intermediate was dissolved in TFA (5 mL) and stirred at RT for 16 h. The acid was removed by rotary evaporation and the crude product was purified by reversed-phase preparative HPLC (MeCN/H_2O gradient with 0.1% TFA). Fractions were collected and concentrated by rotary evaporation. The product was lyophilized to yield **L^7** as a fluffy white solid. **Yield:** 312 mg (69%; 2 steps; based on tBuDO3A·HBr). **NMR** (D_2O, T = 298 K): **^{1}H** (401.0 MHz) δ_H 2.37 (C*H*$_3$, s, 3H); 2.68–3.90 (*mc*, C*H*$_2$–COOH, m, 16+4H); 4.10 (C*H*$_2$–COOH, bs, 4H); 4.54 (C*H*$_2$–arom, bs, 2H); 7.18–7.30 (*arom.*, m, 1H); 7.30–7.45 (*arom.*, m, 1H); 7.49 (*arom.*, dd, 2H, $^3J_{HH}$ = 8, $^4J_{HH}$ = 1). **^{13}C{^{1}H}** (100.6 MHz) δ_C 19.4 (*C*H$_3$, s); 48.9 (*mc*, s); 49.2 (*mc*, s); 50.9 (*mc*, s); 52.4 (*mc*, s); 54.1 (*C*H$_2$–COOH, s); 56.0 (*C*H$_2$–arom, s); 56.3 (*C*H$_2$–COOH, s); 128.3 (*arom.*, s); 128.9 (*arom.*, s); 131.2 (*arom.*, s); 131.3 (*arom.*, s); 132.5 (*arom.*, s); 139.6 (*arom.*, s); 169.7 (*C*O, s); 173.6 (*C*O, s). **ESI-HRMS**: 451.2551 $[M+H]^+$ (theor. $[C_{22}H_{35}N_4O_6]^+$ = 451.2551). **UV absorption:** λ_{max} = 266 nm. **EA** ($C_{22}H_{34}N_4O_6$·1.6TFA·2.0H_2O, M_R = 657.6): C 45.0 (45.2); H 5.7 (6.0); N 8.3 (8.4); F 13.5 (13.6).

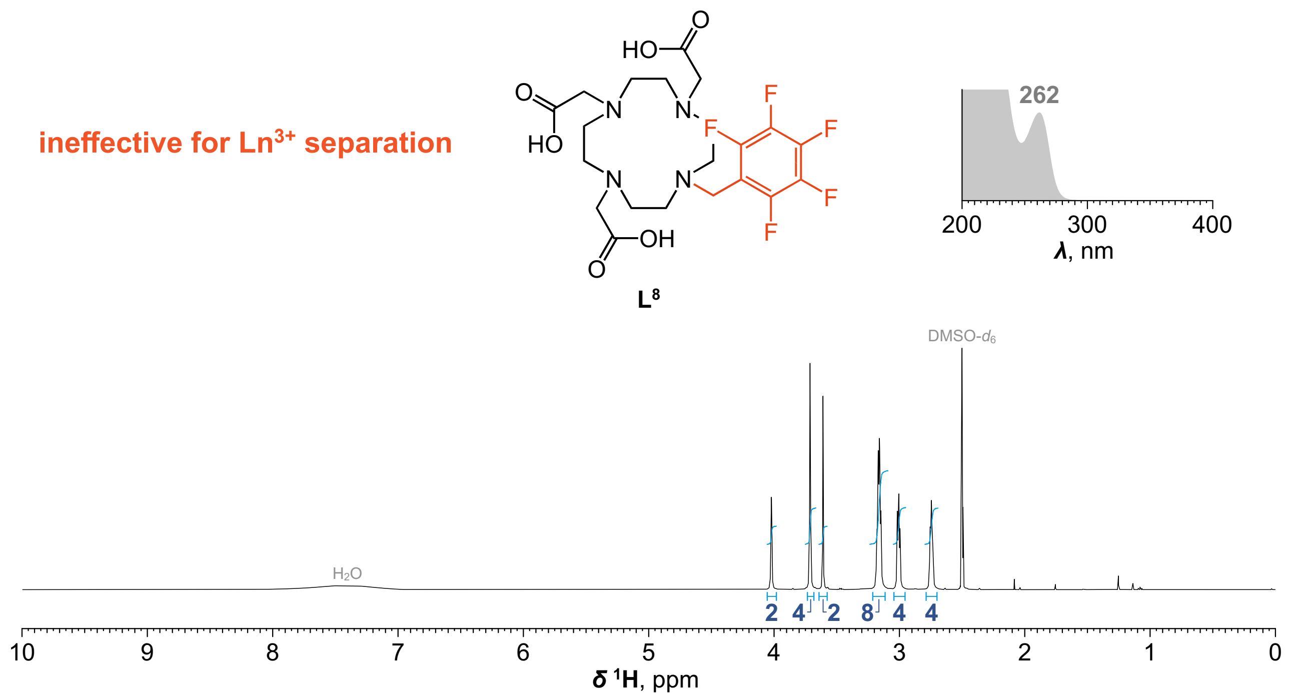

Figure S9. Synthesis and characterization of chelator L^8^. To a solution of tBuDO3A·HBr (404 mg; 0.68 mmol; 1.0 equiv.) in MeCN (25 mL) was added dried K_2CO_3 (396 mg; 2.87 mmol; 4.2 equiv.) and a solution of *1*-(bromomethyl)-*2*,*3*,*4*,*5*,*6*-pentafluorobenzene (193 mg; 0.74 mmol; 1.1 equiv.) in MeCN (2 mL); the reaction was stirred at RT for 16 h. Solids were filtered off and the filtrate was concentrated by rotary evaporation. The crude product was purified by reversed-phase preparative HPLC (MeCN/H_2O gradient with 0.1% TFA). Fractions were collected and rotary evaporated to dryness to yield the alkylated intermediate as a colorless oil. This intermediate was dissolved in TFA (3 mL) and stirred at RT for 16 h. The acid was removed by rotary evaporation and the crude product was re-dissolved in H_2O (1 mL) and loaded onto a solid-phase extraction column (C18, ~0.5 g). The product eluted with distilled H_2O (10 mL). The eluate was lyophilized, giving product $\mathbf{L^8}$ as a fluffy white solid. **Yield:** 345 mg (68%; 2 steps; based on tBuDO3A·HBr). **NMR** (DMSO-*d*~6~, T = 368 K): **^{1}H** (499.9 MHz) δ_H 2.69–2.80 (*mc*, m, 4H); 2.95–3.05 (*mc*, m, 4H); 3.09–3.24 (*mc*, m, 8H); 3.62 (C*H*~2~–COOH, s, 2H); 3.72 (C*H*~2~–COOH, s, 4H); 4.03 (C*H*~2~–arom., s, 2H). **^{13}C{^{1}H}** (125.7 MHz) δ_C 44.9 (*C*H~2~–arom., s); 48.4 (*mc*, s); 49.3 (*mc*, s); 51.7 (*mc*, s); 51.8 (*mc*, s); 53.7 (*C*H~2~–COOH, s); 54.3 (*C*H~2~–COOH, s); 109.7 (*arom.*, t, $^2J_{CF}$ = 20); 137.0 (*arom.*, dm, $^1J_{CF}$ = 249); 140.1 (*arom.*, dm, $^1J_{CF}$ = 251); 145.2 (*arom.*, dm, $^1J_{CF}$ = 245); 169.7 (*C*O, s); 171.0 (*C*O, s). **ESI-HRMS**: 527.1927 $[M+H]^+$ (theor. $[C_{21}H_{28}N_4O_6F_5]^+$ = 527.1924). **UV absorption:** λ_{max} = 262 nm. **EA** ($C_{21}H_{27}N_4O_6F_5$·1.8TFA·1.6H_2O, M_R = 760.4): C 38.9 (39.2); H 4.2 (3.9); N 7.4 (7.0); F 26.0 (26.0).

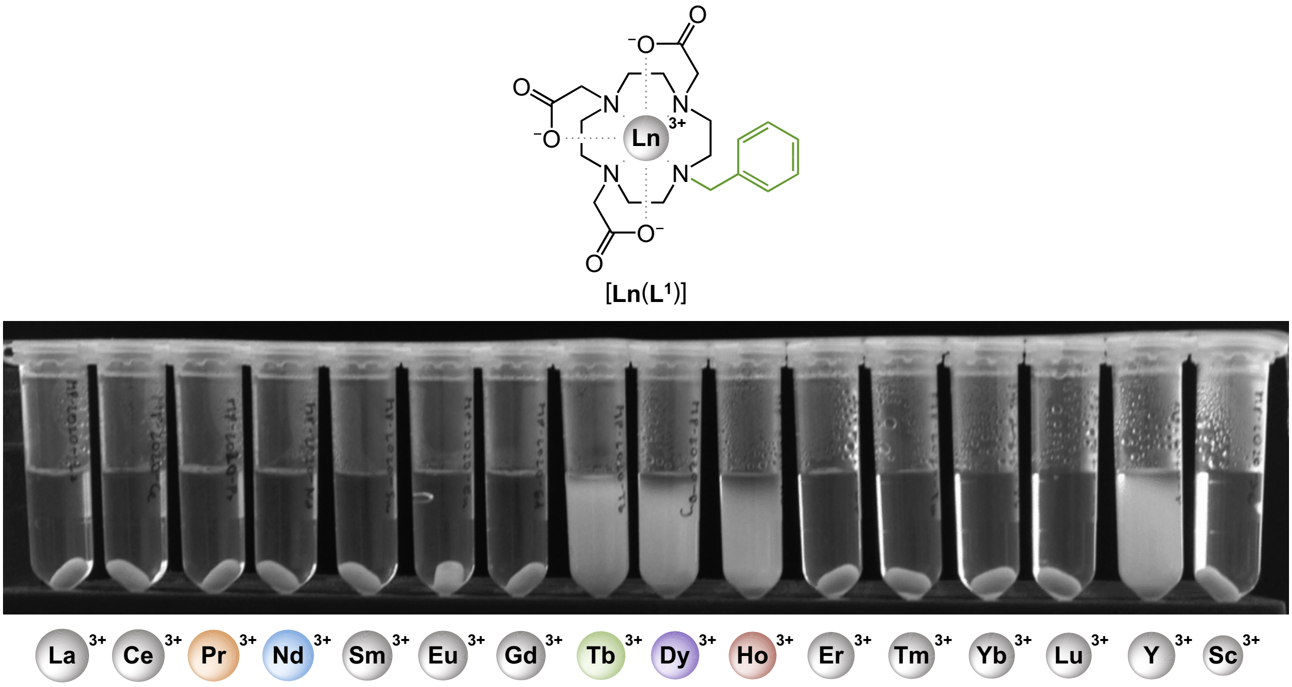

Figure S10. Unexpected solubility pattern of [Ln(L¹)] chelates. Photograph of a series of 5 mM [**Ln**(**L¹**)] solutions in H_2O shows a fine white precipitate formed with Tb^{3+}, Dy^{3+} and Ho^{3+} chelates. The chelate of Y^{3+} (second from the right), which has an ionic radius similar to Dy^{3+}, also precipitated. The chelates from Pr^{3+} to Gd^{3+} could unpredictably precipitate, though they did not in this case (see also Figure 3 in the main text). The photograph was taken a few minutes after stirring was stopped. ***Conditions:*** Eppendorf vials (2 mL) were equipped with magnetic stir bars and charged with 50 µL of 0.1 M stock solution of **L¹**, 50 µL of 0.1 M stock solution of $LnCl_3$, 650 µL of H_2O and 250 µL of 0.1 M stock solution of NaOH (total volume 1.0 mL) while stirring. The reaction mixtures were stirred at RT for 16 h.

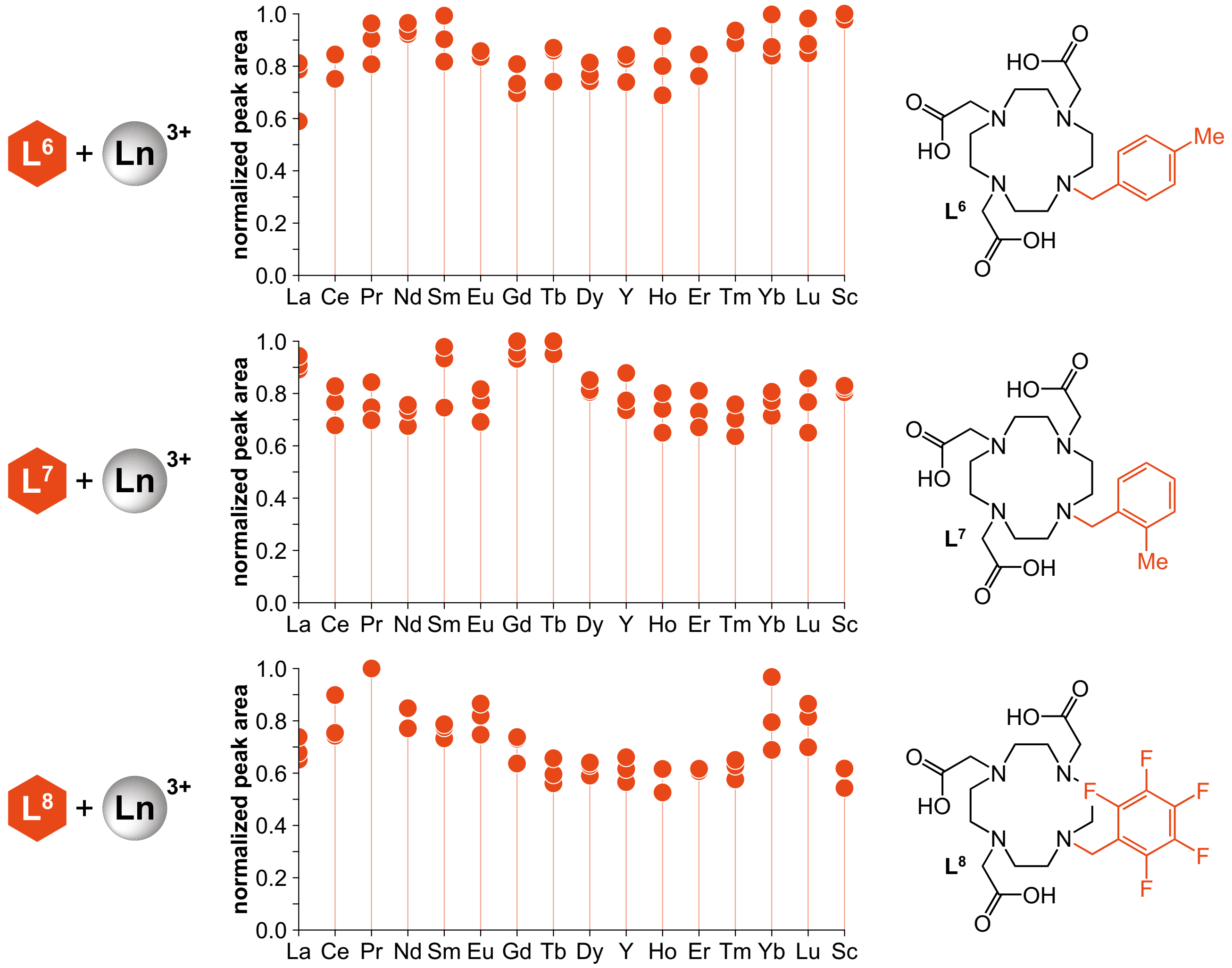

Figure S11. Chelators ineffective for Ln^{3+} separations. Relative solubility profiles of **L^{6}**, **L^{7}** and **L^{8}** chelators within Ln^{3+} series screened by HPLC; extension of Figure 3. In these tests, no precipitation was observed for any Ln^{3+} chelates; these chelators, therefore, were not suitable for Ln^{3+} separations. This behavior differs markedly from that of the parent chelator **L^{1}**, which, with its unsubstituted benzyl arm, demonstrated precipitation for certain Ln chelates in the middle of the series. Chelator **L^{1}** proved quite sensitive to minor changes in reaction conditions (Figure 3 and Figure S10), and these substitutions to the benzyl arm are also detrimental to precipitation of the Ln^{3+} chelates. ***Conditions:*** The solubility profiles for chelators **L^{1}–L^{8}** were obtained by preparing aqueous solutions of the Ln chelates in 96-well plates, with one Ln per well. For each chelator, the series of Ln chelates were prepared in triplicate by mixing aqueous stock solutions of each component for 100 µL of a solution containing 250 mM MOPS/NaOH pH 7 buffer, 5.0 mM $LnCl_3$ and 5.5 mM of the chelator of interest. Magnetic stir bars were used to mix the reactions; the wells were sealed with tape and stirred at RT overnight. After 16 hours, the stir bars were removed and the plates centrifuged; 10-µL aliquots of the supernatants were diluted by H_2O (990 µL) prior to analysis. ***Analysis:*** The supernatants were analyzed by isocratic reversed-phase HPLC using an MeCN/H_2O mobile phase to quantify the chelate remaining in solution by integration of the UV chromatogram at a suitable wavelength; the specific conditions of analysis varied for each chelator: **L^{1}** (20 % MeCN, 200 nm); **L^{2}** and **L^{3}** (30 % MeCN, 290 nm); **L^{4}** (30 % MeCN, 256 nm); **L^{5}** (25 % MeCN, 256 nm); **L^{6}** and **L^{7}** (20 % MeCN; 220 nm); **L^{8}** (25 % MeCN, 220 nm).

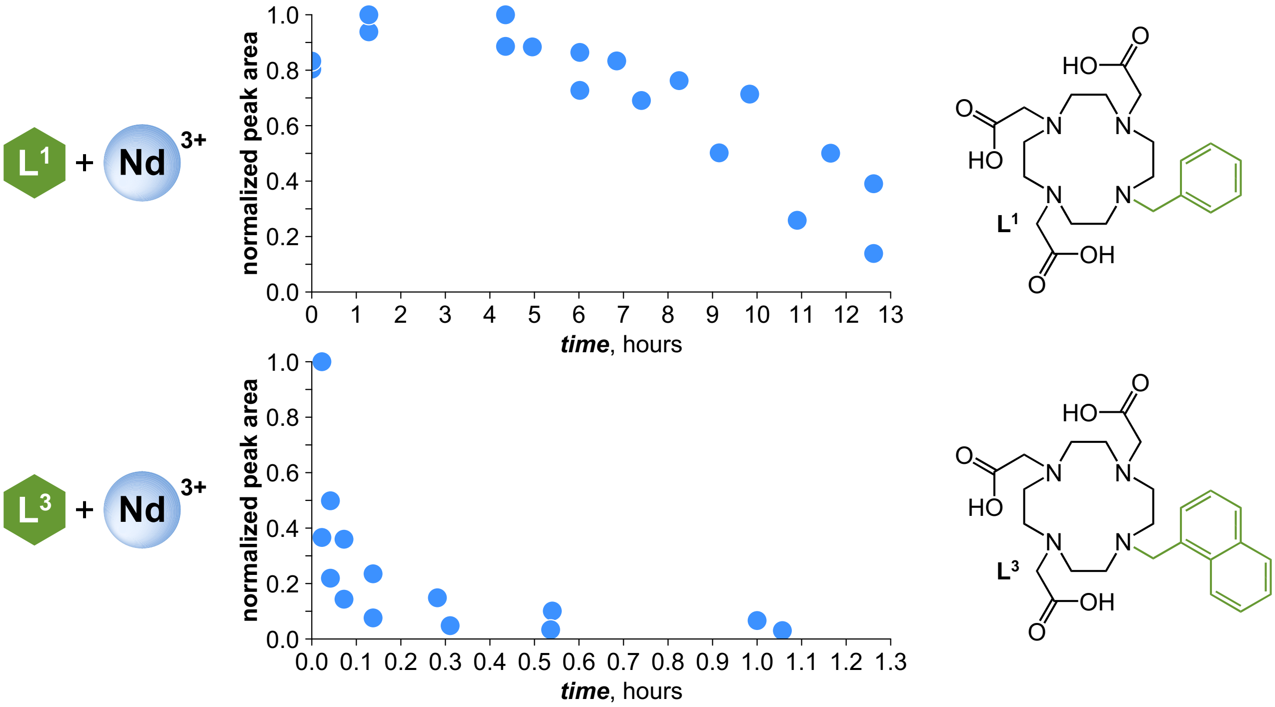

Figure S12. Kinetics of precipitation. The difference in precipitation kinetics as effected by the chelator structure, illustrated by comparing the precipitation of the Nd^{3+} chelates of $\mathbf{L^1}$ (top) and of $\mathbf{L^3}$ (bottom). The time point $t = 0$ h corresponds to the moment of reagent mixing, which marks the onset of both chelate formation and precipitation. ***Conditions:*** Reaction mixtures were prepared in duplicate with 5.0 mM $NdCl_3$, 5.25 mM $\mathbf{L^1}$ or $\mathbf{L^3}$, and 250 mM MOPS/NaOH pH 7 buffer, with a total reaction volume of 1.0 mL. The reactions were stirred at ambient conditions and 30 µL aliquots were sampled at intervals through a 0.45 µm nylon syringe microfilter to remove any precipitate. Each plot shows an overlay of the duplicates. ***Analysis:*** The filtrate was analyzed by reversed-phase HPLC, and the chelate remaining in solution at each time point was quantified by UV peak integration. For [$\mathbf{Nd(L^1)}$], samples were run using an isocratic 20 % MeCN/H_2O (0.1 % FA) mobile phase, and chelate peaks were integrated at 200 nm. For [$\mathbf{Nd(L^3)}$], samples were run using an isocratic 30 % MeCN/H_2O (0.1 % FA) mobile phase, and chelate peaks were integrated at 270 nm.

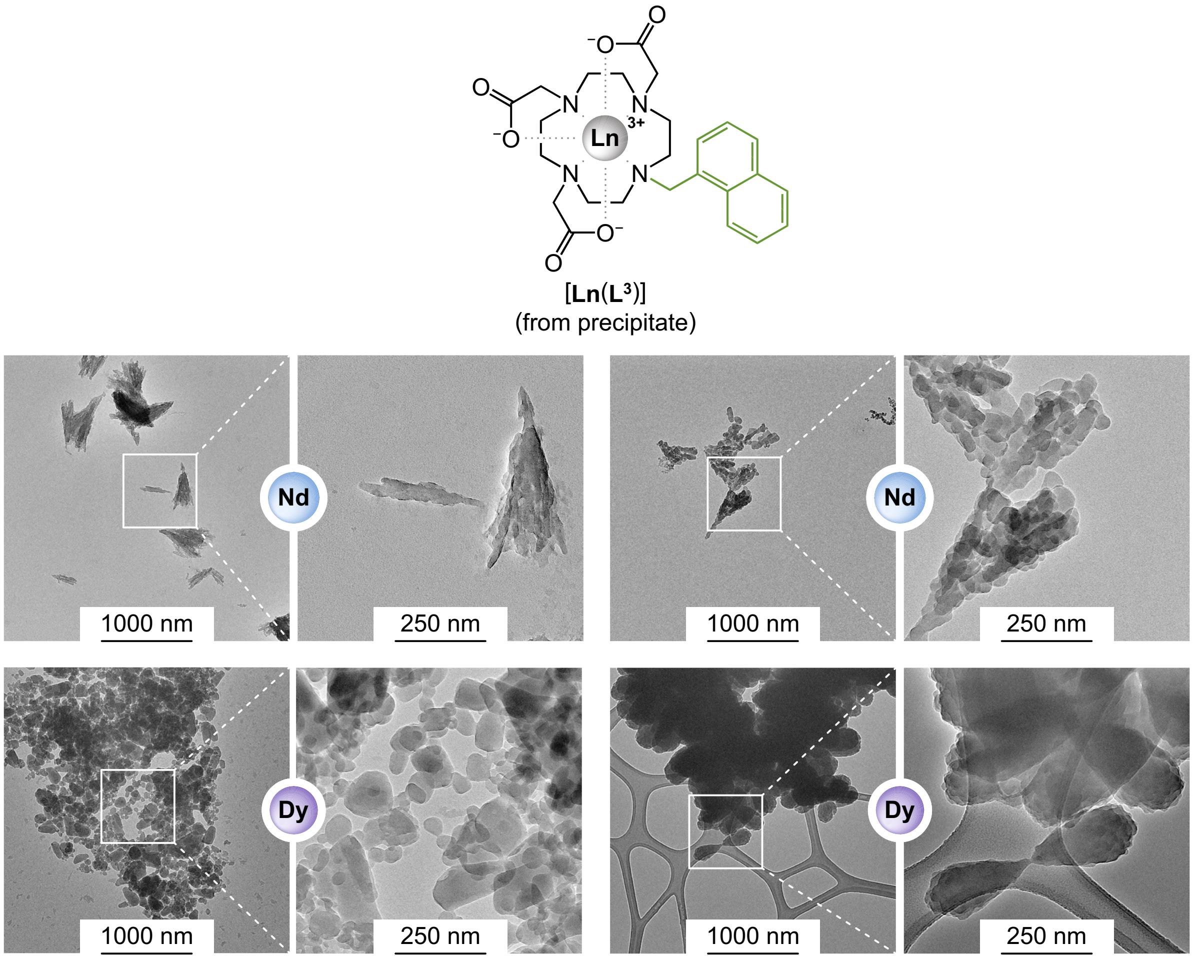

Figure S13. TEM images of [Nd(L^3)] and [Dy(L^3)] precipitates. Duplicated samples of the precipitates were imaged by TEM to examine the particle size and morphology. The Dy precipitate showed higher variability between replicated experiments. In general, it consisted of < 250 nm particles that were of round, irregular boulder-like or pine-cone-like shape. These aggregated into clusters of over micrometer size. The Nd precipitate yielded rather angular, arrowhead-like or needle-like structures about 250 × 500 nm, that themselves consisted of smaller crystal domains on the order of low tens of nanometers. Overall, the TEM analysis did not provide deeper insight, except for confirmation of the existence of very small crystal domains that aligns with the powder X-ray diffraction analysis in Figure S14. ***Conditions:*** Samples of [**Ln(L^3)**] precipitate were obtained for electron microscopy by preparing reaction solutions of ca. 0.5 mL (10 mM Nd^{3+} or Dy^{3+}, 10.5 mM **L^3**, NaOH titration to neutral pH) in 4-mL glass vials; these were mixed by magnetic stirring overnight. The reaction suspensions were then treated for microscopy. For each sample, the suspended precipitate in the reaction solution was mixed by vortexing for ca. 3 s; 5 µl of this suspension were then immediately placed on the carbon side of either a glow-discharged lacey or a continuous carbon TEM grid. The grids were incubated at room temperature for 1 min, washed with distilled water thrice, and air-dried.

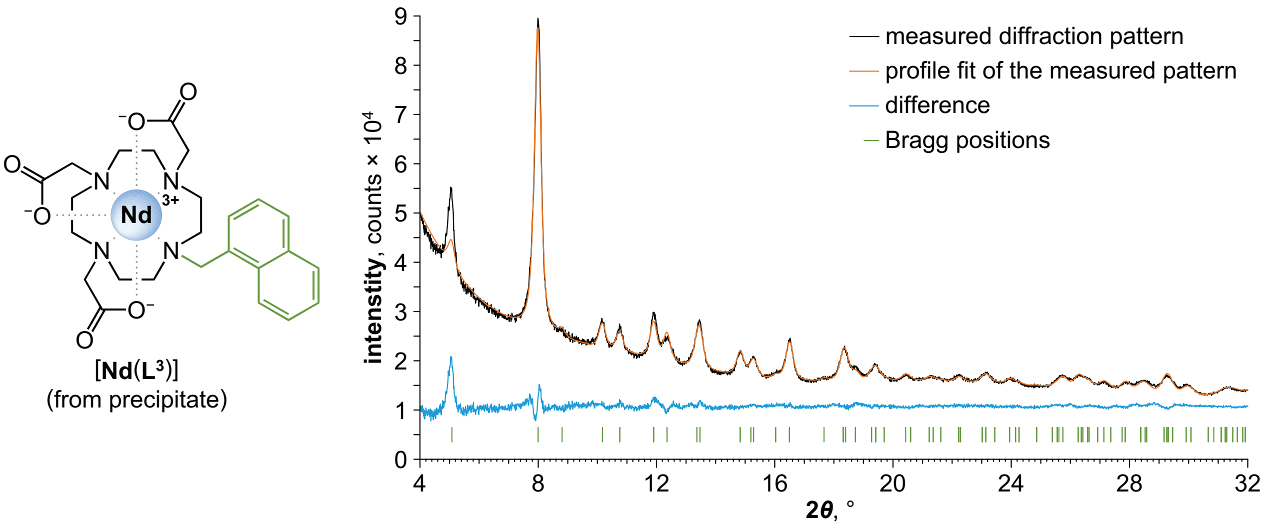

Figure S14. X-ray powder diffraction profile fitting of the [Nd(L^3)] chelate precipitate. The sample was ground and placed in a 0.5 mm borosilicate-glass capillary and measured at 298±5 K over 18 h from 3° to 65° 2*θ* with 0.01° step size and with variable counting time. The collected diffraction pattern indicates poor overall diffraction ability of the sample, and broadened peaks disallowed structural study of this compound. However, the diffraction pattern fits well to the structural model of [**Dy**(**L**^{3})] shown in Figures 4 and Figure S19, suggesting that this [**Nd**(**L**^{3})] precipitate is isostructural with the single-crystal [**Dy**(**L**^{3})]. The whole pattern profile fitting was made in Jana2020[7] with fixed structural model of [**Dy**(**L**^{3})] where the Dy atomic type was changed to Nd. The final fit was made with refined profile, scale and unit cell parameters. Unit cell parameters were refined to a = 20.0640(10) Å, c = 14.3146(15) Å, and V = 4990.5(6) Å^3. The black curve represents the measured pattern, the orange line corresponds to the calculated profile, the cyan curve is the difference between the measured and calculated, and green bars are Bragg positions.

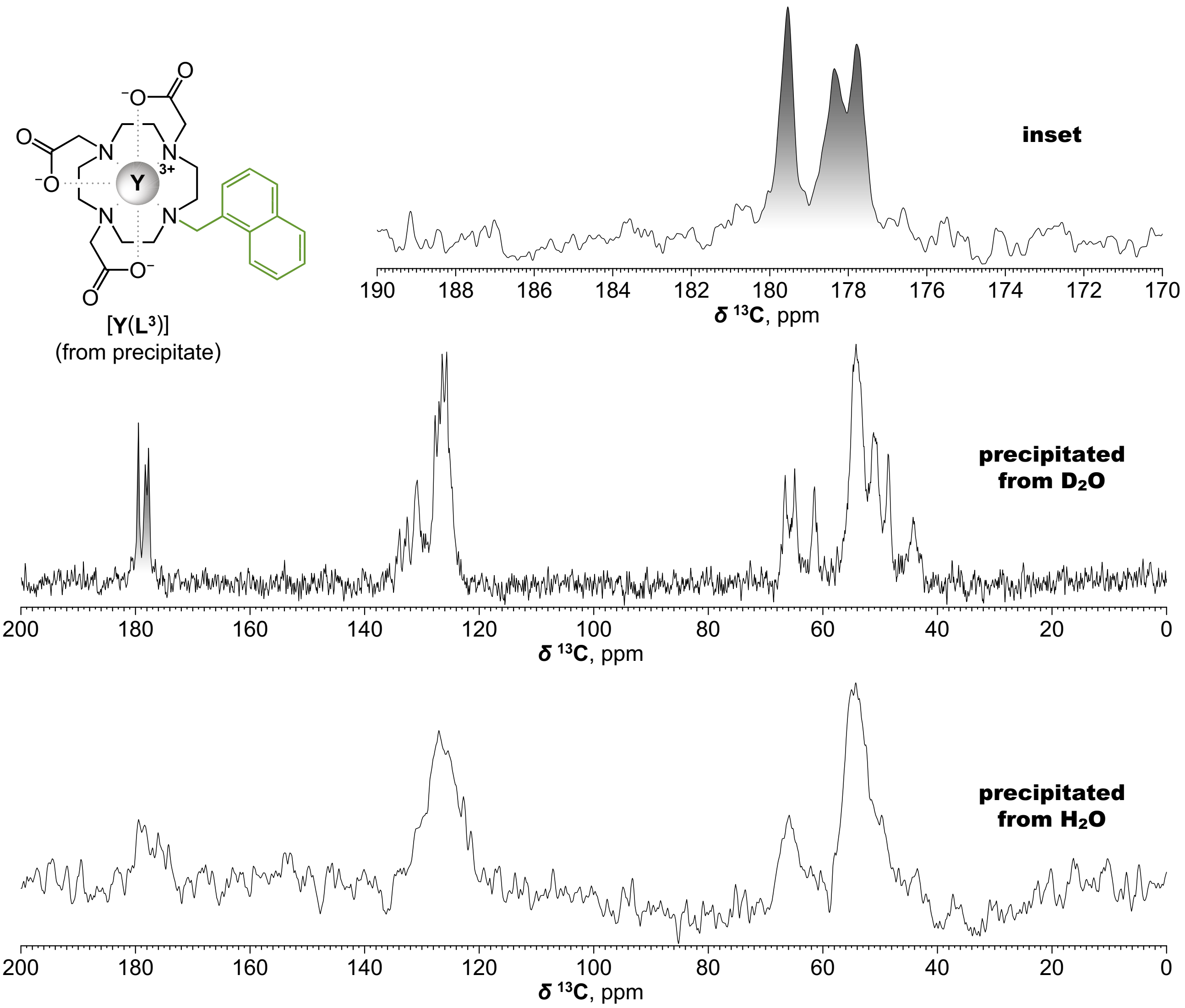

Figure S15. $^{13}C\{^{1}H\}$ CP-MAS spectrum of [Y(L^3)] from precipitate. Attempts were made to obtain structural information for the [**Y**(**L**^{3})] precipitate via solid-state NMR. Precipitate obtained from D_2O yielded a higher-resolution spectrum than that obtained from H_2O, probably due to the elimination of dipolar couplings with structurally disordered crystalline H_2O. The $^{13}C\{^{1}H\}$ CP-MAS spectrum of precipitate from D_2O clearly shows the set of cyclen and pendant CH_2 groups (40–70 ppm) and the naphthyl group (120–140 ppm), as well as three signals corresponding to COO^- groups (shaded; inset). These three well-resolved COO^- signals indicate that [**Y**(**L**^{3})] is present as a pure phase with only a single diastereomer within the solid form. Unfortunately, the heavy overlap of aliphatic signals prevented further estimation of the crystal structure from these spectra. ***Conditions:*** For the H_2O sample, a reaction solution was prepared by joining aqueous stock solutions of **L**^{3} (157 µL of 300 mM stock) and YCl_3 (450 µL of 100 mM stock); this was diluted with 400 µL of H_2O, and titrated by addition of 2.0 M aq. NaOH to pH 5.7, and was then stirred for several hours to precipitate. For the D_2O sample, aqueous stock solutions of **L**^{3} (157 µL of 300 mM stock) and YCl_3 (450 µL of 100 mM stock) were joined, then dried by rotary evaporation; the residue was reconstituted in 500 µL D_2O, dried by rotary evaporation, and once more reconstituted in 500 µL D_2O. This solution was then titrated to neutral pH by a solution of KOH prepared by dissolution of a KOH pellet in D_2O; the reaction was stirred for several hours to precipitate. ***Analysis:*** Samples of obtained precipitates were recorded at fast MAS rate (70 kHz).

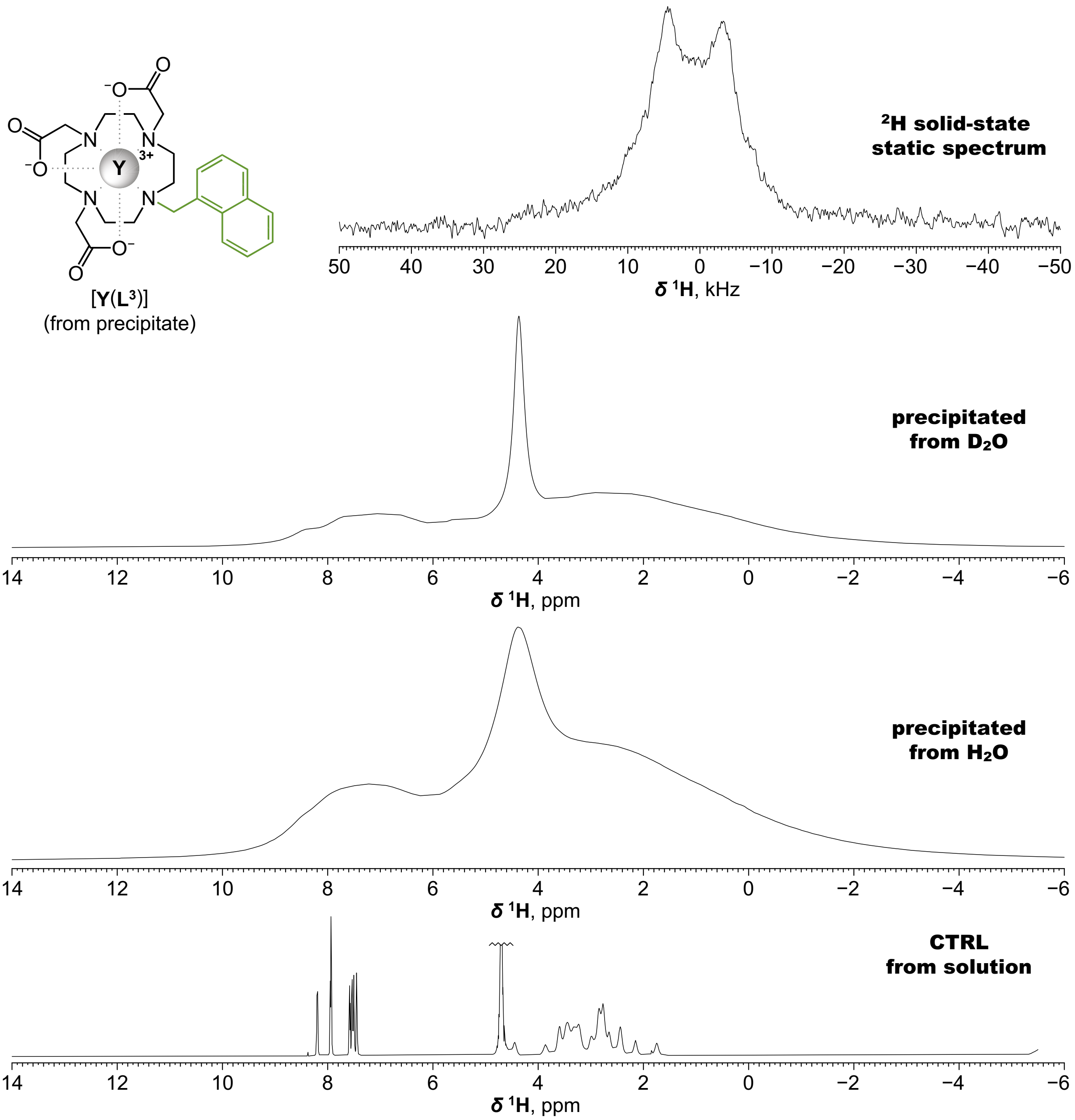

Figure S16. Refutation of OH^{-} bridge hypothesis. To test a hypothesis that the low solubility of [**Ln**(**L**^{3})] could be attributed to intermolecular bonding via bridging OH^{-} groups, spectra were compared for samples of [**Y**(**L**^{3})] precipitated from either H_2O or D_2O. In the case of OH^{-} bridging, the D_2O-precipitated sample would have been expected to yield either a decrease in the ^{1}H MAS signal at 70 kHz MAS, or provide a ^{2}H signal directly corresponding to the presence of bridging OD^{-} groups; this was not the case. The only observed ^{2}H signal was extremely narrow (12 kHz, top spectrum), and likely corresponds to some mobile crystalline water; this signal disappeared after a long period of sample storage, during which the precipitate itself remained intact, and thus further contraindicated the presence of any OD^{-} bridging in the sample. Extremely broad signals in ^{1}H spectra (two in the middle) precluded any detailed analysis. ***Conditions:*** The batch of [**Y**(**L**^{3})] precipitates described in Figure S15 were reused here. ***Analysis:*** ^{1}H solid-state spectra of precipitates from D_2O and H_2O were recorded at a fast MAS rate (70 kHz), while the ^{2}H spectrum was recorded statically (92.1 MHz; without MAS). A solution-state ^{1}H spectrum of [**Y**(**L**^{3})] is given as a control; though the chelate is poorly soluble in water, a sufficient amount of the precipitate could be dissolved in D_2O.

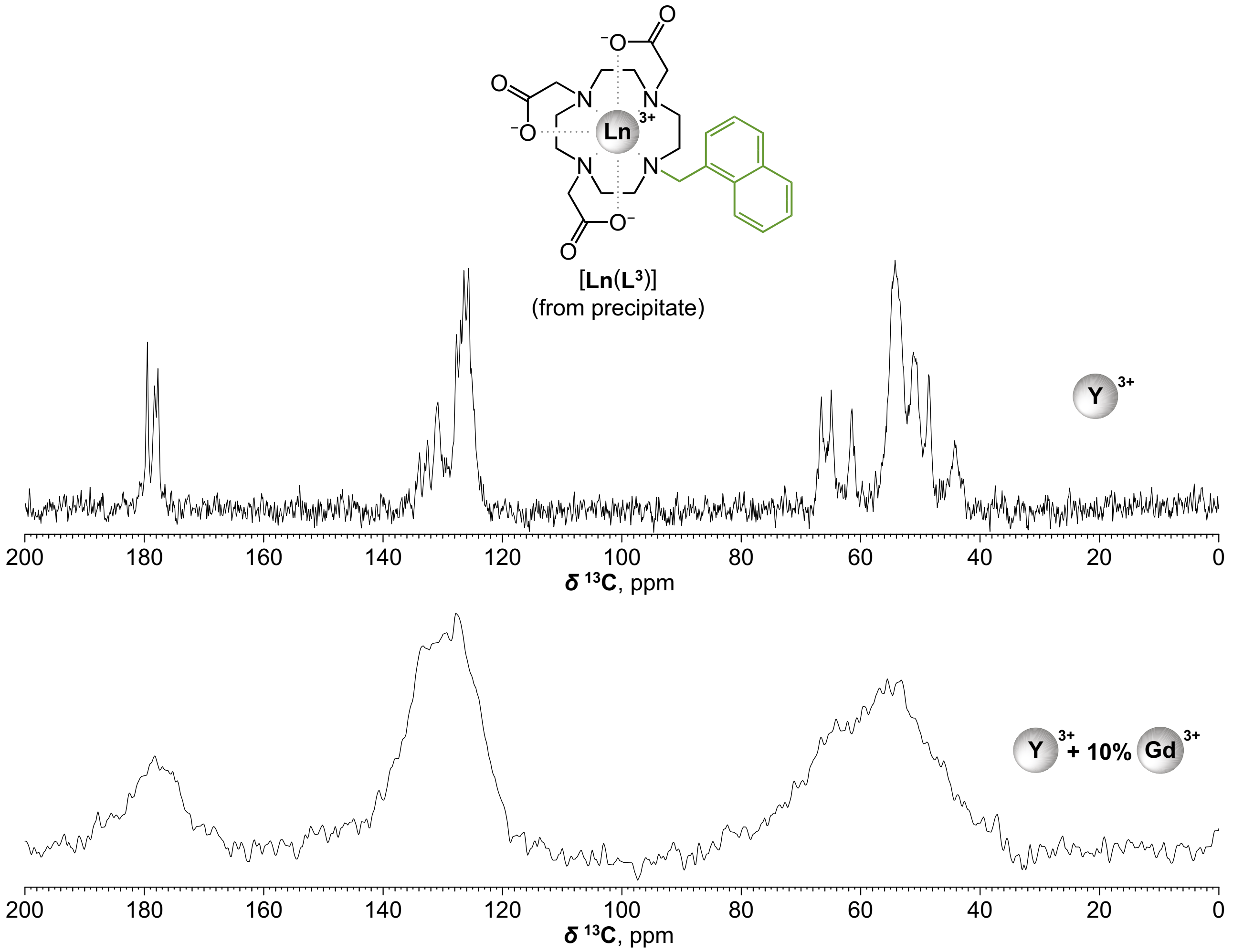

Figure S17. Attempt to confirm carboxylate bridges using $^{13}C\{^{1}H\}$ CP-MAS. Prior to obtaining any single-crystal structural details, an experiment was performed with the goal of proving the presence of intermolecular carboxylate bridges by solid-state NMR. A precipitated sample of [**Y**(**L**^{3})] doped with paramagnetic [**Gd**(**L**^{3})] (9:1 Y:Gd) was expected to demonstrate a decrease in intensity for the ^{13}C signal of the bridging carboxylates between the Y^{3+} and Gd^{3+} chelates. Unfortunately, the presence of paramagnetic Gd^{3+} caused severe broadening of all signals, and signals of individual carboxylic groups could not be resolved (bottom). The $^{13}C\{^{1}H\}$ CP-MAS spectrum of [**Y**(**L**^{3})] precipitate (previously shown in Figure S15) is given for comparison. ***Conditions:*** The reaction solution was prepared by joining **L**^{3} (155 µL of 250 mM stock) and a mixture of YCl_3 and $GdCl_3$ (337 µL of 100 mM YCl_3 + 37 µL of 100 mM $GdCl_3$); the reaction solution was diluted by the addition of 400 µL H_2O, followed by aq. NaOH titration to pH 6.0. The reaction was stirred overnight at RT, then dried (first by rotary evaporation, and then under high vacuum for two days). ***Analysis:*** The dried sample of mixed Y^{3+}/Gd^{3+} precipitate was analysed with a fast MAS rate (70 kHz) in a direct-excitation ^{13}C experiment.

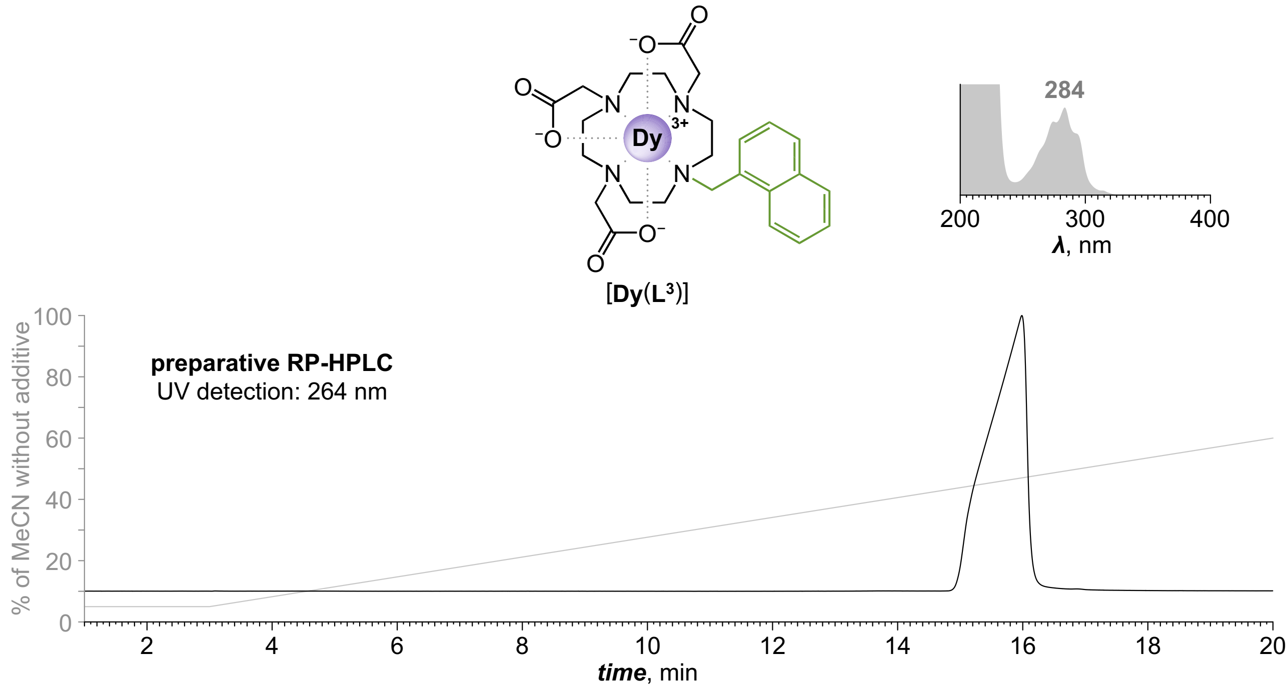

Figure S18. Synthesis and characterization of [Dy(L³)] chelate. In a glass vial (4 mL), **L³**·2.0HCl·3.0H_2O·0.1*i*-PrOH (29.5 mg; 50.2 µmol; 1.0 equiv.) was dissolved in DMSO* (1.6 mL), followed by the addition of aq. MOPS/NaOH pH 7 buffer (3.0 M; 1.0 mL; 3.0 mmol; 60 equiv.) and aq. $DyCl_3$ (100 mM; 600 µL; 60.0 µmol; 1.2 equiv.); the mixture was stirred at RT for 15 min. This mixture was then filtered through syringe microfilter (RC) and the filtrate was directly purified by preparative HPLC (C18; H_2O–MeCN gradient without additive)#. Fractions with product were joined and directly lyophilized to give product as white fluffy solid. **Yield:** 33.5 mg (90%; 1 step; based on **L³**·2.0HCl·3.0H_2O·0.1*i*-PrOH). **ESI-HRMS:** 648.1613 $[M+H]^+$ (theor. $[C_{25}H_{32}N_4O_6Dy_1]^+$ = 648.1608). **UV absorption:** λ_{max} = 284 nm. **EA** ($[C_{25}H_{31}N_4O_6Dy_1]\cdot5.7H_2O$, M_R = 748.7): C 40.1 (40.4); H 5.7 (5.4); N 7.5 (7.2).

* Note: The use of DMSO as a co-solvent was neccessary to prevent premature/undesired precipitation of the [**Dy**(**L³**)] chelate before reversed-phase HPLC purification; MeOH and *i*-PrOH would serve the same purpose.

\# Note: Due to the low kinetic inertness of [**Dy**(**L³**)] chelate towards acid-assisted dechelation, the use of FA/TFA additive for either analytical or preparative reversed-phase HPLC is inadvisable. In such a case, on-column dechelation occurs, and peaks of both the intact [**Dy**(**L³**)] and free **L³** would be observed. However, since [**Dy**(**L³**)] is electroneutral, chromatography is feasible even using pure solvents without any ion-pairing additives, and the pure chelate can be easily isolated.

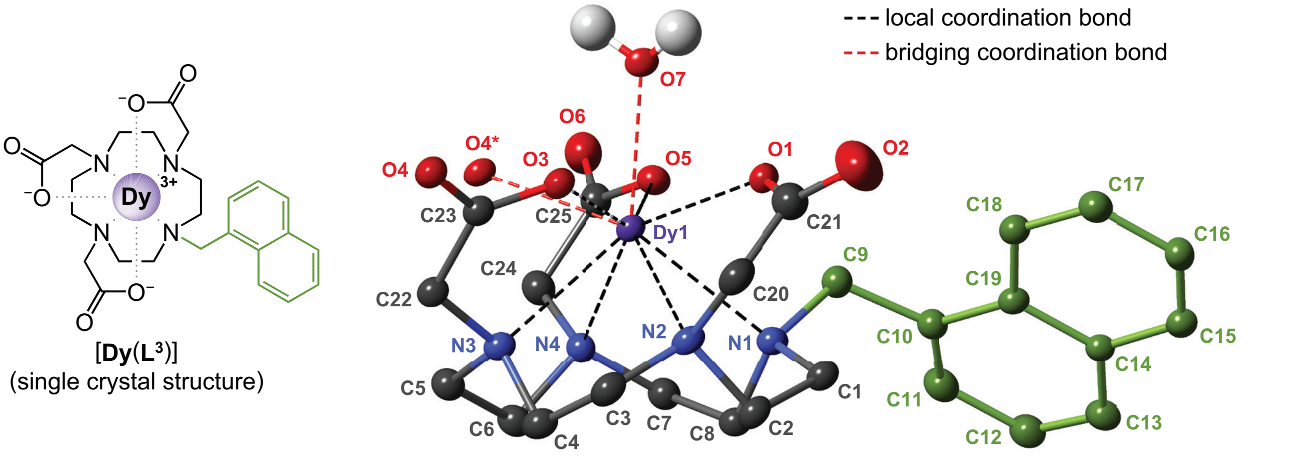

formula	M_W	system	space group	a, Å	b, Å	c, Å	α, °	β, °	γ, °	radiation
$C_{75}H_{131.4}N_{12}O_{37.2}Dy_3$	2284.01	hexagonal	$P6_3$	19.548(2)	19.548(2)	14.816(1)	90	90	120	Cu/K_α

D_{calc}, g × cm^{-3}	Z	V, Å^3	R_1 [$I \geq 2\sigma(I)$]	T, K	$2\theta_{min}$, °	$2\theta_{max}$, °	GOF	$F(000)$	μ, mm^{-1}	CCDC
1.547	2	4902.89(9)	3.68%	100.00(11)	7.93	157.79	1.17	2322	12.78	2419472

Figure S19. Solid-state structure of [Dy(L³)] chelate. Structure of [**Dy**(**L³**)(H_2O)] unit found in the crystal structure of {[**Dy**(**L³**)(H_2O)]}$_3$·16.2H_2O. Single crystal was obtained by diffusion of H_2O vapors into the solution of [**Dy**(**L³**)]·5.7H_2O (0.5 mg, prepared according to procedure described in Figure S18) in DMSO (25 µL) over the course of several months. The coordination environment around the Dy^{3+} cation is irregular: chelator **L³** binds the metal ion by four cyclen nitrogen atoms and three acetate arms, while the napthalene arm is, as expected, pointing away from the coordination center. The coordination number of 9 for the Dy^{3+} cation is completed by one H_2O molecule (O7) and one carboxyl moiety from neighbouring chelate unit within the trimer motif (O4*). The polyhedral shape of the coordination environment around the Dy^{3+} cation is very close to spherical capped square antiprism or muffin.[8] Non-coordinated water molecules as well as carbon-bound hydrogen atoms were omitted for clarity. Thermal ellipsoids were set at 25% probability. Selected crystallographic parameters are summarized in the table. Crystallographic data for structural analysis have been deposited with the Cambridge Crystallographic Data Centre (CCDC no. 2419472). Copies of this information may be obtained free of charge from http://www.ccdc.cam.ac.uk.

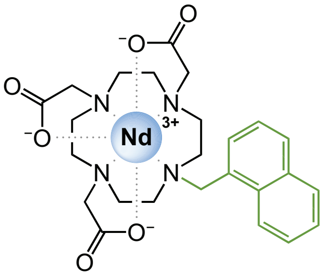

[Nd(L^3)]
(from precipitate)

[Nd(L^3)]

**system**	hexagonal
**space group**	$P6_3$
a, Å	20.064
b, Å	20.064
c, Å	14.315

isostructural

**system**	hexagonal
**space group**	$P6_3$
a, Å	19.548
b, Å	19.548
c, Å	14.816

[Dy(L^3)]

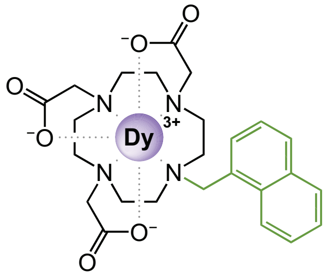

[Dy(L^3)]
(from single crystal)

Figure S20. Isostructurality of [Ln(L^3)] chelates. Comparison of unit cell parameters obtained from [**Nd**(**L^3**)] powder X-ray diffraction (Figure S14) and from [**Dy**(**L^3**)] single-crystal X-ray diffraction (Figure S19) demonstrates excellent agreement between the two structures, including the same hexagonal $P6_3$ space group.

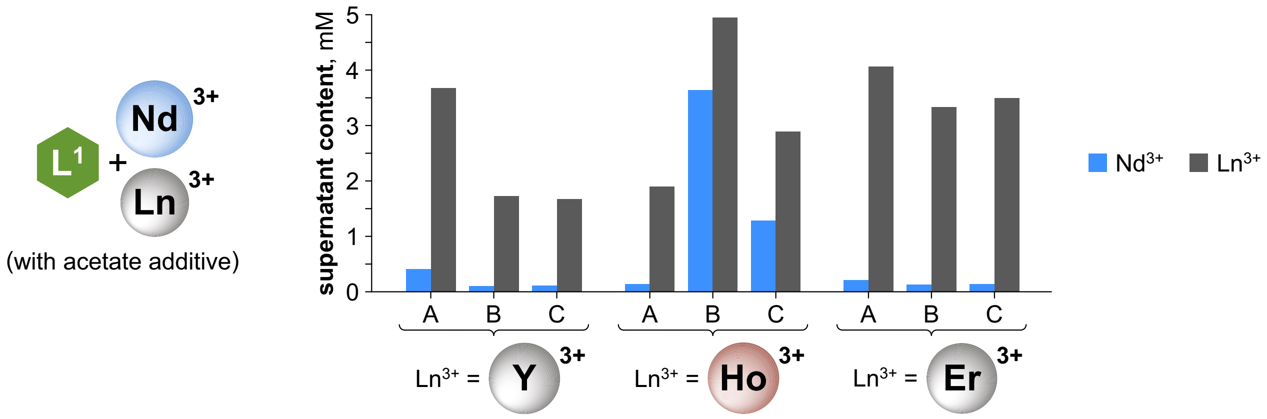

Figure S21. Reproducibility of precipitation at solubility profile break in [Ln(L³)] series. In the binary Nd^{3+}/Ln^{3+} screening with **L³** and acetate (main text Figure 5, bottom plot), the Nd^{3+}/Ho^{3+} pair behaved unexpectedly—the [**Ho**(**L³**)] chelate remained much less soluble than was to be expected from the behaviors of the adjacent Nd^{3+}/Y^{3+} and Nd^{3+}/Er^{3+} pairs from that same series (here, series A). The [**Ln**(**L³**)]-acetate separations for Nd^{3+}/Ho^{3+} (as well as Nd^{3+}/Y^{3+} and Nd^{3+}/Er^{3+} as control samples, to test the standard variation in behavior) were therefore repeated twice more for verification (here, series B and C). Rather than confirming the initial outlier results, the behavior of the Nd^{3+}/Ho^{3+} pair varied significantly across those triplicates—even as the Nd^{3+}/Y^{3+} and Nd^{3+}/Er^{3+} pairs yielded rather consistent results. The control pairs of Nd^{3+}/Y^{3+} and Nd^{3+}/Er^{3+} both gave the expected low supernatant content of Nd^{3+}, while the [**Y**(**L³**)] and [**Er**(**L³**)] chelates were more soluble; this consistency indicated that the results of these methods were generally reproducible, and that the inconsistent behavior of the Nd^{3+}/Ho^{3+} pair in these conditions must be specific to the presence of Ho^{3+} ions. This variability was therefore attributed to the position of Ho on the break point of the **L³** solubility profile (main text Figure 5). The [**Ho**(**L³**)] showed higher variability of precipitation even as a single Ln chelate, and is thus apparently sensitive to even very minor, unintentional changes to the reaction conditions (perhaps the precise concentration of **L³** and/or acetate). Interestingly, the behavior of [**Nd**(**L³**)] in the same mixed Nd^{3+}/Ho^{3+} samples was also irregular in a similar way, indicating a complex relationship between the two chelates, probably resulting from intermolecular interactions. ***Conditions:*** Reaction solutions were prepared in a 96-well plate by mixing aqueous stock solutions of each component for 100 µL of a solution containing 250 mM MOPS/NaOH pH 7 buffer, 5 mM $NdCl_3$, 5 mM $LnCl_3$ (Ln = Y, Ho, Er) and 11 mM chelator **L³**. Magnetic stir bars were used to mix the reactions; the wells were sealed with tape and stirred at ambient conditions overnight. After 16 h, the stir bars were removed and the plates were centrifuged. ***Analysis:*** The supernatants were analyzed by ICP-OES for Ln quantitation.

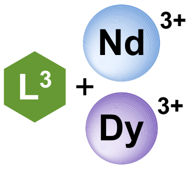

(with different additives in various quantities)

additive type	additive equiv.	supernatant, mM Nd^{3+}	supernatant, mM Dy^{3+}	precipitate, mM Nd^{3+}	precipitate, mM Dy^{3+}	SF	additive type	additive equiv.	supernatant, mM Nd^{3+}	supernatant, mM Dy^{3+}	precipitate, mM Nd^{3+}	precipitate, mM Dy^{3+}	SF
–	–	0.07	0.37	4.98	4.60	5.5	fluoride	0.5	0.13	1.21	6.21	4.87	12
acetate	5	0.33	3.05	4.97	2.43	19		1.0	0.18	2.34	6.15	3.82	20
	10	0.30	3.74	4.85	1.74	35		1.5	0.49	3.26	5.68	2.72	14
	25	1.17	4.37	4.04	1.05	14	*α*-HIBA	0.5	0.10	1.55	4.32	2.59	27
	50	4.62	4.69	0.52	0.49	1.1		1.0	0.19	3.03	4.03	1.50	43
benzoate	5	0.10	2.69	4.66	1.81	72		1.5	0.33	3.37	4.33	1.18	37
	10	0.49	4.03	4.60	0.97	39		2.0	0.60	3.69	3.78	0.81	28
	15	1.03	4.34	3.96	0.68	25	chloride	10	0.24	0.29	4.62	4.58	1.2
	20	2.89	4.20	1.65	0.53	4.6		20	0.12	0.22	4.57	4.48	1.8
citrate	0.1	0.14	0.38	4.30	4.03	2.9		50	0.11	0.24	4.39	4.29	2.2
	0.5	0.19	1.24	4.77	3.62	8.6		100	0.11	0.24	4.97	4.88	2.2
	1.0	0.25	2.27	4.49	2.38	17	lactate	0.5	0.51	1.59	4.33	3.15	4.3
	2.0	0.66	3.52	4.08	1.21	18		1.0	0.21	2.47	4.49	2.08	26
formate	5	0.07	0.51	5.17	4.33	8.5		1.5	0.49	3.21	4.38	1.60	18
	10	0.42	1.09	4.85	3.84	3.2		2.0	0.50	3.57	4.33	1.18	26
	25	0.11	1.68	5.38	3.33	25	nitrate	20	0.15	0.41	3.97	3.89	2.9
	50	0.22	2.51	4.93	2.09	27		50	0.14	0.82	4.46	4.16	6.2
glycine	5	0.09	1.01	4.90	3.60	15		100	0.15	1.72	4.61	3.23	17
	10	0.42	1.99	4.61	2.79	7.8	sulfate	25	0.08	0.47	4.96	4.09	7.4
	15	0.11	2.25	4.91	2.44	42		50	0.59	1.00	4.30	3.57	2.1
	20	0.28	2.85	4.69	1.86	26		100	0.34	1.20	4.61	3.35	4.8
glycolate	0.5	0.09	1.73	4.64	2.61	33	thiocyanate	10	0.14	0.31	4.66	4.46	2.3
	1.0	0.20	2.93	4.92	1.71	43		20	0.41	0.51	4.60	4.42	1.3
	1.5	0.35	3.13	4.54	1.11	37		50	0.36	0.72	4.65	4.16	2.2
	2.0	0.64	3.82	4.33	0.82	32		100	0.41	1.70	4.63	3.26	5.8

Table S1. Effect of various additives on Nd^{3+}/Dy^{3+} separation in the [Ln(L^3)] system. Various additives were screened over range of concentrations for their effect on the precipitation-based separations of the Nd^{3+}/Dy^{3+} pair using chelator $\mathbf{L}^3$. ***Conditions:*** For each additive, a group of (usually) four reaction solutions were prepared in plastic cuvettes, each with a total reaction volume of 2 mL. Each contained 5 mM each of $NdCl_3$ and $DyCl_3$, 11 mM of $\mathbf{L}^3$, 500 mM of MOPS/NaOH pH 7 buffer, and a variable quantity of the additive of interest. When possible, sodium salts of the additives were used; otherwise, the additive stock solutions were titrated to pH 7 by addition of NaOH prior to preparation of the reaction solutions. The order of stock solution additions to the reaction vessels was as follows: $LnCl_3$, $\mathbf{L}^3$ and H_2O (to make up the necessary volume), followed by simultaneous addition of the MOPS/NaOH buffer and the additive. These reactions were stirred overnight, at which point a 0.5 mL aliquot of the suspension was taken from each. This aliquot was centrifuged; the supernatant was separated and the precipitate dissolved in dilute HCl. ***Analysis:*** The Nd^{3+} and Dy^{3+} content of each supernatant and precipitate were quantified by ICP-OES, and separation factors (SF, as defined in Figure 6) values were calculated for each to help determine the optimal additives to improve the separations achieved by this process; SF values >10 were rounded to integers for clarity.

	A1		A2		A3		A4	
**magnet mass**, g	17.8		7.7		8.5		6.0	
**solution volume**, mL	88.3		63.8		71.2		41.5	
**solution mass**, g	132.6		86.9		96.1		58.0	
**elements**	**ppm**	**mass**	**ppm**	**mass**	**ppm**	**mass**	**ppm**	**mass**
B	1549	0.9%	1192	0.9%	980	0.9%	1143	0.9%
Al	296	0.2%	267	0.2%	225	0.2%	237	0.2%
Ca	127	0.1%	94	0.1%	57	0.1%	61	0.0%
Ti	305	0.2%	243	0.2%	199	0.2%	232	0.2%
Fe	116458	66.2%	87888	65.0%	71601	64.7%	85446	65.2%
Co	4194	2.4%	4250	3.1%	3629	3.3%	4113	3.1%
Cu	287	0.2%	251	0.2%	184	0.2%	214	0.2%
Pr	n.d.	–	n.d.	–	n.d.	–	n.d.	–
Nd	46864	26.6%	36398	26.9%	29941	27.0%	35154	26.8%
Tb	1096	0.6%	917	0.7%	709	0.6%	707	0.5%
Dy	911	0.5%	712	0.5%	n.d.	–	n.d.	–
Ho	3887	2.2%	3040	2.2%	3166	2.9%	3673	2.8%

	B1		B2		B3		B4	
**magnet mass**, g	3.65		3.2		4.2		3.2	
**solution volume**, mL	25.3		29.9		25		25.45	
**solution mass**, g	35.3		40.0		35.4		35.2	
**elements**	**ppm**	**mass**	**ppm**	**mass**	**ppm**	**mass**	**ppm**	**mass**
B	1254	0.9%	1420	0.9%	949	0.9%	1050	0.9%
Al	254	0.2%	291	0.2%	197	0.2%	224	0.2%
Ca	242	0.2%	275	0.2%	187	0.2%	207	0.2%
Ti	n.d.	–	n.d.	–	n.d.	–	n.d.	–
Fe	92048	65.4%	104246	65.5%	67502	64.8%	76534	65.0%
Co	2991	2.1%	3334	2.1%	2343	2.2%	2584	2.2%
Cu	246	0.2%	282	0.2%	185	0.2%	205	0.2%
Pr	9934	7.1%	12080	7.6%	7704	7.4%	8923	7.6%
Nd	30803	21.9%	33810	21.2%	22814	21.9%	25338	21.5%
Tb	627	0.4%	901	0.6%	520	0.5%	618	0.5%
Dy	n.d.	–	n.d.	–	n.d.	–	n.d.	–
Ho	2303	1.6%	2574	1.6%	1759	1.7%	1975	1.7%

Table S2. Elemental analyses of real magnets. Eight magnets from two different electric automotive motors (A and B) were processed to enable ICP-OES analysis and to determine the specific Ln content of each. ***Conditions:*** For each, the magnet (or a fragment thereof) was digested in 65% nitric acid, by addition of the concentrated nitric acid in ca. 5 mL aliquots at intervals of 0.5–2 h, until the digestion was complete. Any undissolved resin coating was removed by filtration through an S4 sintered glass filter, during which process the solution was further diluted by H_2O. ***Analysis:*** The resulting solutions were analyzed by ICP-OES to determine the lanthanides present, as well as any other major elements of interest (n.d. = not detected). The mass composition was calculated as % weight of the magnet.

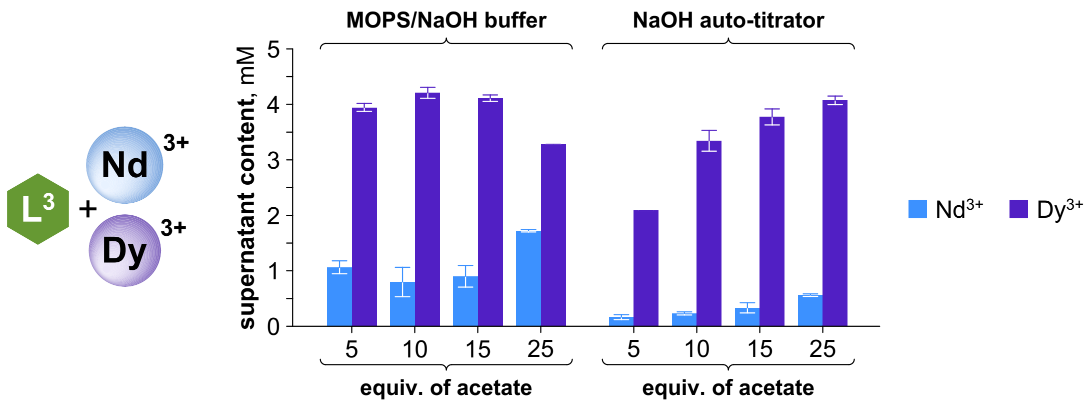

Figure S22. Buffer vs. titration pH control. For small-scale preliminary screenings of reaction conditions, the use of MOPS buffer (pH 7) was preferred as a means of pH control. When running larger-scale reactions, however, the use of such large quantities of buffer was undesirable, and so the reaction pH was adjusted by NaOH titration. The results indicate that the method of pH control does not negatively impact the separations achieved by chelator $\mathbf{L^3}$. The auto-titration device was configured by connecting a pH probe and a 12-port syringe pump to an Arduino microcontroller. A program was written to enable the pH of the reaction solution to be read in real time, and for 5 µL aliquots of either 2.0 M or 0.2 M NaOH to be added to the reaction solution at intervals of ca. 2–5 seconds until the desired pH of 6.0 was reached; this ensured that the NaOH titration of the reaction solutions would be more consistent across experiments than would be possible by manual addition of NaOH. The use of NaOH titration tended to provide heavier precipitation of [$\mathbf{Nd}$($\mathbf{L^3}$)] chelate from solution compared to the use of buffer. This was attributed to the slight solubilising effect of the MOPS buffer: though it was not such a strong additive as acetate or *α*-HIBA, the presence of 50 equivalents of this buffer in the reaction solution nevertheless interfered with the aggregation responsible for the precipitation of these chelates. ***Conditions:*** Two sets of experiments (either buffered with MOPS/NaOH pH 7 buffer or titrated with NaOH; each in duplicate) were conducted in 2 mL Eppendorf centrifugation vials (buffered set) or 4 mL glass vials (titrated set). Reaction solutions were prepared by joining aqueous stock solutions of $\mathbf{L^3}$, $NdCl_3$ and $DyCl_3$. For the buffered set, sodium acetate (5–25 equiv.) in aq. MOPS/NaOH pH 7 buffer was added to start the experiment (overall volume 250 µL with final concentration of 5 mM $NdCl_3$, 5 mM $DyCl_3$, 11 mM $\mathbf{L^3}$ and 50 mM MOPS/NaOH buffer). For the titrated set, AcOH (5–25 equiv.) was added first to each reaction solution, followed by titration to a final reaction pH of about 6.5 by the addition of aq. NaOH using the auto-titration device. The reaction solutions were prepared to have a post-titration reaction volume of 1000 µL; this was done by calculating the expected quantity of NaOH required for titration (as the equiv. of AcOH present plus 5 additional equiv. to account for the acidic stock solutions of the chelator and Lns). Actual reaction volumes ranged from 995 to 1030 µL. Each vial contained a PTFE-coated magnetic stir bar, and the reactions were stirred at RT overnight. ***Analysis:*** The stir bars were removed and the vials centrifuged, and the supernatants were analysed by ICP OES to quantify the Nd^{3+}/Dy^{3+} content of each sample.

1:1 initial ratio

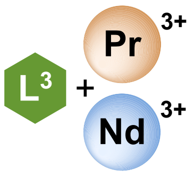

(with acetate additive)

(30 equiv. of acetate, 10 mM target Ln^{3+} reaction concentration, 1 h run times)

round	sample	supernatant, mM		precipitate, mM		precipitated fraction	SF Pr/Nd
		Pr^{3+}	Nd^{3+}	Pr^{3+}	Nd^{3+}		
0	initial	–	–	5.52	5.89	–	n.a.
1	A	0.77	1.20	5.12	4.31	83%	1.9
	B	0.91	1.34	3.85	3.24	76%	1.7
	C	0.81	1.26	4.21	3.59	79%	1.8
2	A	1.02	1.37	4.35	3.28	76%	n.a.
	B	1.04	1.34	4.03	2.96	75%	n.a.
	C	1.29	1.60	4.05	2.96	71%	n.a.
3	A	0.87	1.14	4.67	3.09	79%	n.a.
	B	0.83	1.05	4.39	2.85	79%	n.a.
	C	1.52	1.54	3.24	1.97	63%	n.a.

Table S3. Optimized, repeated separation of Pr^{3+}/Nd^{3+} pair (1:1 initial ratio). Efficient reprocessing of the precipitate phase is demonstrated for optimized separations for the **L^3**-Ln-acetate system with the Pr/Nd pair, on a small scale (ca. 2 mL reaction volume) for three rounds of precipitation (in triplicates, as A–C). Separation factors (SF) were calculated only for the first rounds of separations, where the initial ratio of Lns is 1:1 (n. a. = not applicable). ***Conditions:*** The initial reaction solution was prepared by joining appropriate volumes of aqueous stock solutions (**L^3**, $PrCl_3$, $NdCl_3$), HOAc and H_2O, and then the reaction was initiated by titrating the sample to ca. pH 6.0 by addition of aq. NaOH. Analytical aliquots (50 µL) were centrifuged so that the supernatant could be isolated from the precipitate; the precipitate was dissolved by addition of 100 µL 0.5 M aq. HCl, and the supernatant was diluted by addition of 50 µL 1.0 M aq. HCl. These samples were analyzed by HPLC to approximate the degree of precipitation (by the chelator content in each phase), in order to scale the next round of precipitation appropriately.* The bulk reaction precipitate was dissolved in 1.0 M aq. HCl with heating at 80 °C for about 1 h, and appropriate volumes of H_2O and 1.0 M aq. HOAc were added to the vial, followed by aq. NaOH titration to pH 6.0 to start the precipitation. This process was repeated for a total of three rounds of [**Ln**(**L^3**)]-acetate separations. Note that when preparing the reaction solutions, the quantity of NaOH which would be added during the titration process was predicted by calculation; this sometimes led to small discrepencies between the target reaction concentration and the actual reaction concentration. ***Analysis:*** The analytical aliquots from both precipitate and supernatant were analyzed by ICP-OES to quantify the Pr^{3+}/Nd^{3+} content of each sample.

* Note: These samples were analyzed by HPLC-DAD-UV to approximate the degree of precipitation and scale the next round of precipitation appropriately. For this HPLC analysis, a 10-µL aliquot of the sample was treated with 170 µL H_2O and 20 µL 0.5 M acetate/NaOH buffer (pH 5.8), then heated at 80 °C for 30 min to ensure chelation prior to analysis with an isocratic HPLC method of 24 % MeCN/H_2O on a LunaOmega C18 column. [**Ln**(**L^3**)] peak areas were integrated at 270 nm, and the degree of precipitation was determined by comparison of the peak areas for each supernatant and precipitate.

1:1 initial ratio

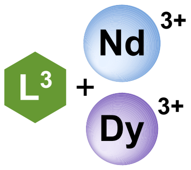

(with acetate additive)

(10 equiv. of acetate, 10 mM target Ln^{3+} reaction concentration, 3 h run times)

round	sample	supernatant, mM		precipitate, mM		precipitated	SF
		Nd^{3+}	Dy^{3+}	Nd^{3+}	Dy^{3+}	fraction	Nd/Dy
0	initial	–	–	8.23	9.95	–	n.a.
1	A	0.48	8.00	7.75	1.95	53%	66
	B	0.23	7.55	8.00	2.11	57%	125
	C	0.40	7.70	7.98	2.29	56%	66
2	A	0.27	1.27	7.10	0.71	84%	n.a.
	B	0.29	1.36	7.62	0.78	84%	n.a.
	C	0.30	1.35	7.14	0.76	83%	n.a.
3	A	0.31	0.52	8.48	0.39	91%	n.a.
	B	0.63	0.62	7.52	0.27	86%	n.a.
	C	0.28	0.55	7.81	0.39	91%	n.a.

Table S4. Optimized, repeated separation of Nd^{3+}/Dy^{3+} pair (1:1 initial ratio). Efficient reprocessing of the precipitate phase is demonstrated for optimized separations for the **L^3**-Ln-acetate system with the Nd/Dy pair, on a small scale (ca. 2 mL reaction volume) for three rounds of precipitation (in triplicates, as A–C). Separation factors (SF) were calculated only for the first rounds of separations, where the initial ratio of Lns is 1:1 (n. a. = not applicable). ***Conditions:*** Analogous to Pr/Nd repeated precipitation (Table S3) with $NdCl_3$ and $DyCl_3$ instead of the aforementioned pair of Lns. ***Analysis:*** The analytical aliquots from both precipitate and supernatant were analyzed by ICP-OES to quantify the Nd^{3+}/Dy^{3+} content of each sample.

1:1 initial ratio

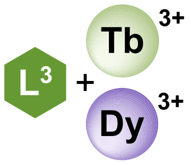

(with acetate additive)

(1.5 equiv. of acetate, 25 mM target Ln^{3+} reaction concentration, 2 h run times)

round	sample	supernatant, mM		precipitate, mM		precipitated fraction	SF Tb/Dy
		Tb^{3+}	Dy^{3+}	Tb^{3+}	Dy^{3+}		
0	initial	0.36	0.60	14.14	13.51	97%	n.a.
1	A	0.89	2.10	18.60	15.32	92%	2.9
	B	0.90	2.16	11.00	9.05	87%	2.9
	C	1.00	2.32	10.87	8.82	86%	2.9
2	A	2.81	4.11	8.19	5.11	66%	n.a.
	B	2.59	4.03	9.98	6.37	71%	n.a.
	C	3.26	4.46	8.84	5.42	65%	n.a.
3	A	3.85	3.52	6.41	2.86	56%	n.a.
	B	3.66	3.65	7.95	3.68	61%	n.a.
	C	4.74	3.88	5.32	2.29	47%	n.a.

Table S5. Optimized, repeated separation of Tb^{3+}/Dy^{3+} pair (1:1 initial ratio). Efficient reprocessing of the precipitate phase is demonstrated for optimized separations for the **L**^{3}-Ln-acetate system with the Tb/Dy pair, on a small scale (ca. 2 mL reaction volume) for three rounds of precipitation (in triplicates, as A–C). Separation factors (SF) were calculated only for the first rounds of separations, where the initial ratio of Lns is 1:1 (n. a. = not applicable). ***Conditions:*** Analogous to Pr/Nd repeated precipitation (Table S3) with $TbCl_3$ and $DyCl_3$ instead of aforementioned pair of cations. For this pair, a preliminary round of precipitation was run overnight in the absence of acetate additive, to precipitate the majority of the chelate and leave any excess chelator in solution, as these slight excesses of chelator were found to hinder precipitation for these more-sensitive [**Tb**(**L**^{3})]/[**Dy**(**L**^{3})] chelates. ***Analysis:*** The analytical aliquots from both precipitate and supernatant were analyzed by ICP-OES to quantify the Tb^{3+}/Dy^{3+} content of each sample.

1:1 initial ratio

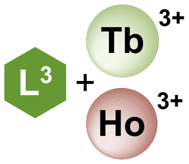

(with acetate additive)

(1.5 equiv. of acetate, 25 mM target Ln^{3+} reaction concentration, 2 h run times)

round	sample	supernatant, mM		precipitate, mM		precipitated	SF
		Tb^{3+}	Ho^{3+}	Tb^{3+}	Ho^{3+}	fraction	Tb/Ho
0	initial	0.14	0.39	6.27	6.11	96%	n.a.
1	A	4.46	8.00	7.75	3.98	48%	3.5
	B	4.90	8.07	6.93	3.48	45%	3.3
	C	5.77	8.61	6.06	2.95	39%	3.1
2	A	2.28	3.55	11.64	3.77	73%	n.a.
	B	2.67	3.52	10.44	3.11	69%	n.a.
	C	4.98	4.08	6.79	1.72	48%	n.a.
3	A	3.65	2.97	13.50	2.74	71%	n.a.
	B	2.98	2.44	11.18	2.15	71%	n.a.
	C	7.21	2.29	2.75	0.42	25%	n.a.

Table S6. Optimized, repeated separation of Tb^{3+}/Ho^{3+} pair (1:1 initial ratio). Efficient reprocessing of the precipitate phase is demonstrated for optimized separations for the $\mathbf{L^3}$-Ln-acetate system with the Tb/Ho pair, on a small scale (ca. 2 mL reaction volume) for three rounds of precipitation (in triplicates, as A–C). Separation factors (SF) were calculated only for the first rounds of separations, where the initial ratio of Lns is 1:1 (n. a. = not applicable). ***Conditions:*** Analogous to Pr/Nd repeated precipitation (Table S3) with $TbCl_3$ and $HoCl_3$ instead of aforementioned pair of cations. For this pair, a preliminary round of precipitation was run overnight in the absence of acetate additive, to precipitate the majority of the chelate and leave any excess chelator in solution, as these slight excesses of chelator were found to hinder precipitation for these more-sensitive [**Tb**($\mathbf{L^3}$)]/[**Ho**($\mathbf{L^3}$)] chelates. ***Analysis:*** The analytical aliquots from both precipitate and supernatant were analyzed by ICP-OES to quantify the Tb^{3+}/Ho^{3+} content of each sample.

1:5 initial ratio

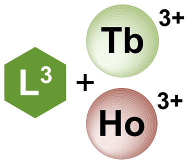

(with acetate additive)

(1.0 equiv. of acetate, 25 mM target Ln^{3+} reaction concentration, 5 h run times)

round	sample	supernatant, mM		precipitate, mM		precipitated fraction	SF Tb/Ho
		Tb^{3+}	Ho^{3+}	Tb^{3+}	Ho^{3+}		
0	initial	0.08	1.36	4.11	19.53	94%	n.a.
1	A	0.10	3.93	4.65	18.41	85%	n.a.
	B	0.19	6.02	3.96	14.39	75%	n.a.
	C	0.24	6.62	4.18	14.73	73%	n.a.
2	A	0.12	3.83	4.34	14.24	82%	n.a.
	B	0.15	3.84	5.30	16.01	84%	n.a.
	C	0.17	4.40	4.42	12.08	78%	n.a.
3	A	0.14	3.18	5.22	14.74	86%	n.a.
	B	0.17	3.55	5.75	14.63	85%	n.a.
	C	0.20	3.87	5.19	11.09	80%	n.a.

Table S7. Optimized, repeated separation of Tb^{3+}/Ho^{3+} pair (1:5 initial ratio). Efficient reprocessing of the precipitate phase is demonstrated for optimized separations for the **L^3**-Ln-acetate system with the Tb/Ho pair, on a small scale (ca. 2 mL reaction volume) for three rounds of precipitation (in triplicates, as A–C). Separation factors are not calculated here as the starting ratio is far from 1:1 (n.a. = not applicable). ***Conditions:*** Analogous to Pr/Nd repeated precipitation (Table S3) with $TbCl_3$ and $HoCl_3$ instead of aforementioned pair of cations and different initial ratio of Lns. For this pair, a preliminary round of precipitation was run overnight in the absence of acetate additive, to precipitate the majority of the chelate and leave any excess chelator in solution, as these slight excesses of chelator were found to hinder precipitation for these more-sensitive [**Tb**(**L^3**)]/[**Ho**(**L^3**)] chelates. ***Analysis:*** The analytical aliquots from both precipitate and supernatant were analyzed by ICP-OES to quantify the Tb^{3+}/Ho^{3+} content of each sample.

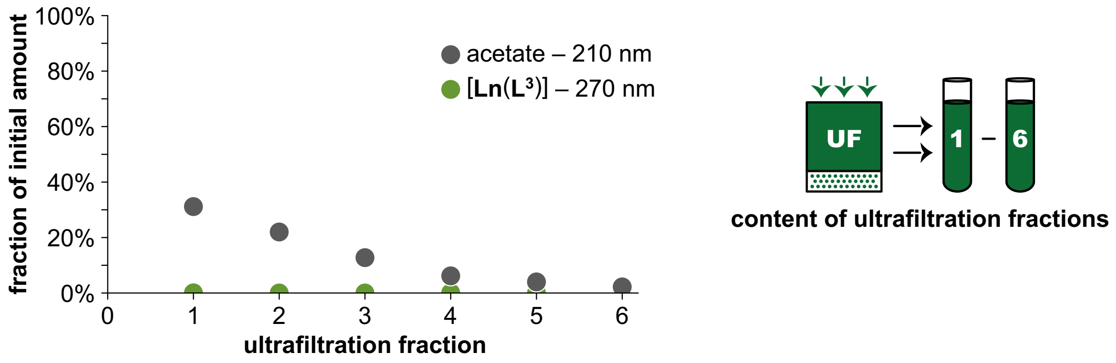

Figure S23. Removal of acetate additive by ultrafiltration. Demonstration of the efficacy of ultrafiltration for removal of acetate additive from solution after one round of precipitation. ***Conditions:*** A test separation was run by preparing a 10-mL reaction mixture, containing chelator $\mathbf{L^3}$ (1.1 equiv.), a quaternary mixture of Lns (Nd^{3+}:Pr^{3+}:Tb^{3+}:Dy^{3+}, in a 3.3:1:1:1 ratio to simulate a recycling Ln feed stock; 1.0 equiv. total Ln^{3+} content), and acetic acid (10 equiv.). This was titrated to pH 6.2 by addition of aq. NaOH (2.0 M and 0.2 M) to trigger precipitation. The reaction was stirred at RT for 2 hours, at which time the precipitate was removed by use of a 0.45 µm RC syringe microfilter. The pH of the filtered solution was brought down to 3.2 by addition of aq. HCl (1.0 M). This solution was filtered through an NFS nanofiltration membrane (*Synder filtration*) by applying N_2 gas (ca. 4 bar). A 5-mL fraction was collected, the filtration was halted, 5 mL of H_2O were added to the solution once more, and filtration was resumed for collection of another 5-mL fraction. This was repeated for collection of 6 fractions in total, each 5 mL in volume. ***Analysis:*** The filtrate fractions were analyzed by HPLC; details are given in Figure S25. Data are presented as the molar fraction of acetate or [$\mathbf{Ln(L^3)}$] present in the respective collected fraction relative to the quantity present in the solution before ultrafiltration.

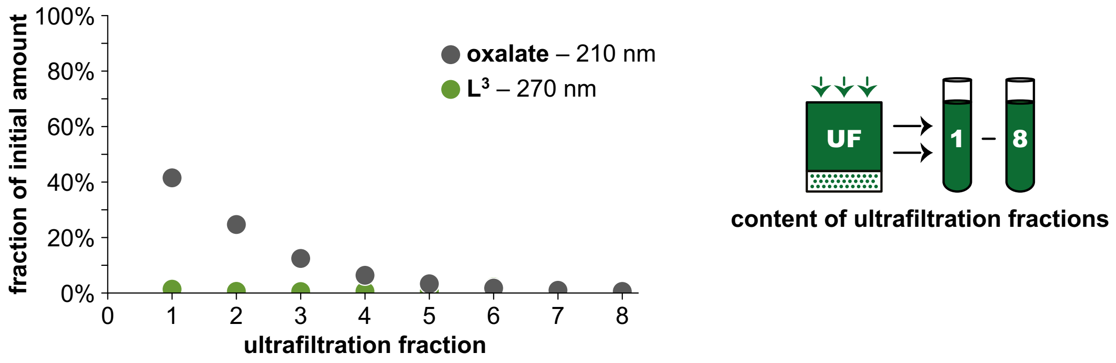

Figure S24. Removal of oxalic acid by ultrafiltration. Demonstration of the efficacy of ultrafiltration for removal of oxalate from solution after one round of precipitation. ***Conditions:*** A test solution of the chelator and a lanthanide was prepared, with $\mathbf{L^3}$ (5.5 mM) and $PrCl_3$ (5 mM), for a total volume of 5 mL, with a pH of 3.0. Oxalic acid (10 equiv., based on Pr^{3+} = 1.0 equiv.) was added, and the solution was stirred for 1 hour to precipitate the Pr^{3+} oxalate. This suspension had a pH of 1.4. The solid was removed by filtration through a 0.45 µm nylon syringe filter, and H_2O was added to bring the total solution volume to 10 mL. This solution was filtered through an NFS nanofiltration membrane (*Synder filtration*) by applying N_2 gas (ca. 4 bar). A 5-mL fraction was collected, the filtration was halted, 5 mL of H_2O were added to the solution once more, and filtration was resumed for collection of another 5-mL fraction. This was repeated for collection of 8 fractions in total, each 5 mL in volume. ***Analysis:*** The filtrate fractions were analyzed by HPLC; details are given in Figure S26. Data are presented as the molar fraction of oxalate or chelator $\mathbf{L^3}$ present in the respective collected fraction relative to the quantity present in the solution before ultrafiltration.

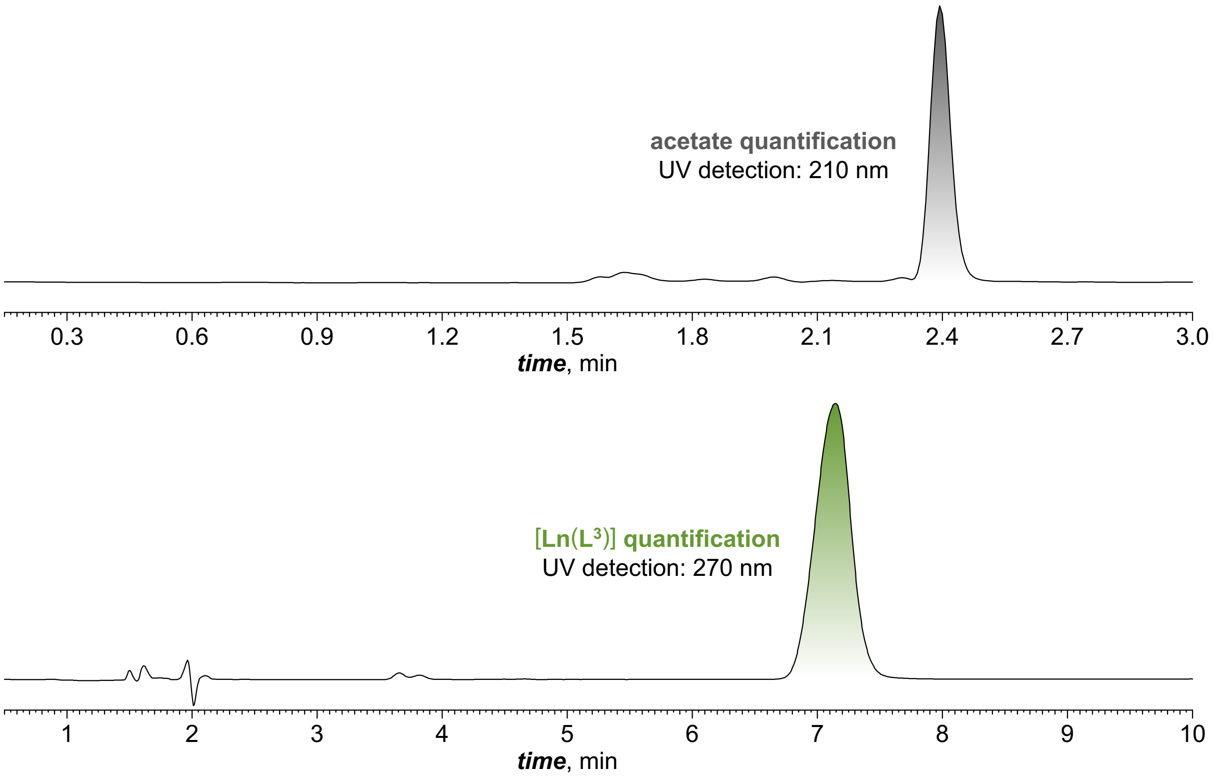

Figure S25. Quantification of acetate additive removal by ultrafiltration. Representative analytical HPLC chromatograms used to monitor progress of acetate additive removal (and [**Ln**(**L**^{3})] retention) during ultrafiltration. These chromatograms given as examples are from analysis of the retained solution after collection of the first fraction, for the experiment described in Figure S23. ***Conditions:*** Samples were run with isocratic methods on a LunaOmega C18 column. For acetate quantitation, a mobile phase of 5 % MeCN in 25 mM phosphate buffer (pH 2.5) was used*; for [**Ln**(**L**^{3})] quantitation, a mobile phase of 20 % MeCN/80 % H_2O was used#. ***Analysis:*** The peak areas were integrated to quantify the acetate (210 nm; black peak) and [**Ln**(**L**^{3})] (270 nm; green peak) present in each sample. The HPLC samples for quantitation of the [**Ln**(**L**^{3})] chelate were treated with aq. MOPS/NaOH pH 7 buffer and heated at 80 °C for 30 minutes to ensure full complexation (partial de-chelation occurred during the low-pH ultrafiltration) prior to analysis.

* Note: Phosphate buffer was chosen due to its negligible absorption at 210 nm (no interference with acetate quantification) and was prepared by dissolving the $Na_2HPO_4 \cdot 12H_2O$ salt in H_2O and titrating to the desired pH (2.5) by HCl/NaOH additions—stock solution of this buffer was then used to prepare the mobile phase.

# Note: Neutral conditions (without acidic additives) were chosen to avoid de-chelation of [**Ln**(**L**^{3})].

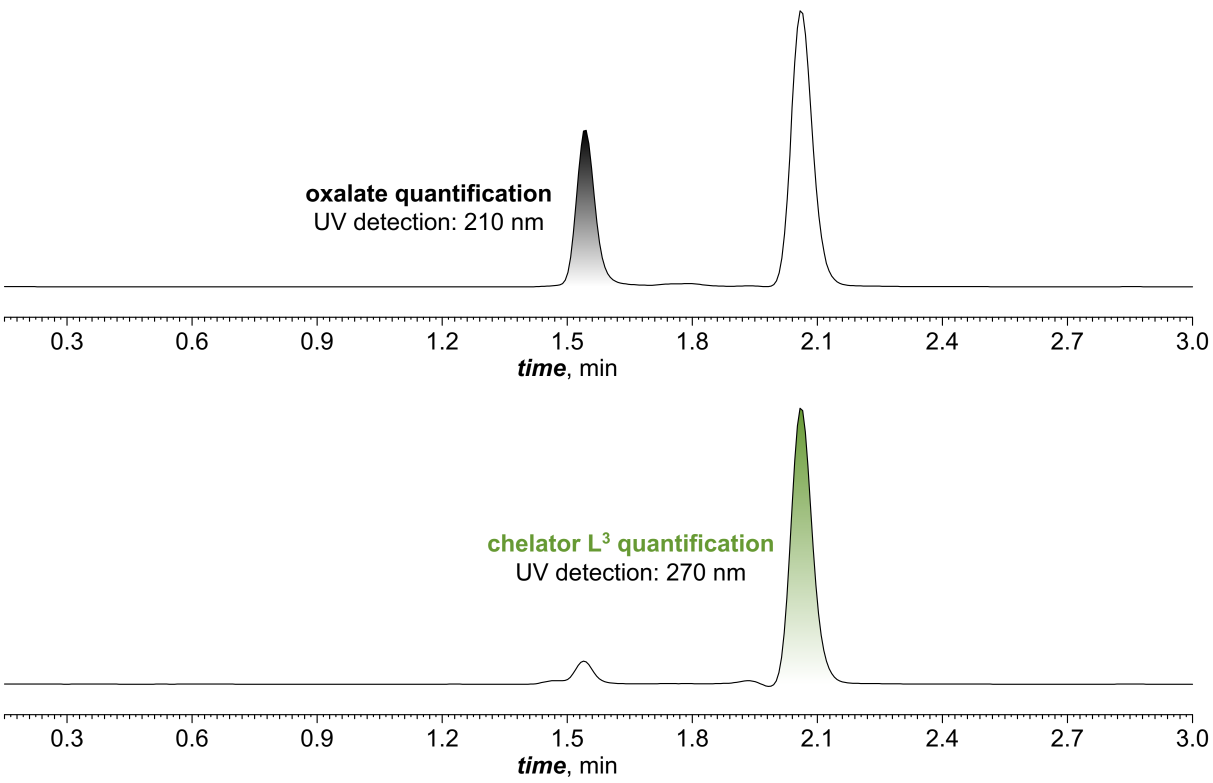

Figure S26. Quantification of oxalate removal by ultrafiltration. Representative chromatograms used to monitor progress of oxalate removal (and $\mathbf{L^3}$ retention) during ultrafiltration. These chromatograms given as examples are from analysis of the retained solution after collection of the first fraction, for the experiment described in Figure S24. ***Conditions:*** Samples were run with isocratic methods on a LunaOmega C18 column. For oxalate quantitation, a mobile phase of 5 % MeCN in 50 mM phosphate buffer (pH 2.5) was used*; for $\mathbf{L^3}$ quantitation, a mobile phase of 30 % MeCN in 50 mM phosphate buffer (pH 2.5) was used#. ***Analysis:*** The peak areas were integrated to quantify the oxalate (210 nm; black peak) and $\mathbf{L^3}$ chelate (270 nm; green peak) present in each sample.

* Note: Phosphate buffer was chosen due to its negligible absorption at 210 nm (no interference with oxalate quantification) and its preparation is described in Figure S25.

# Note: Acidic conditions were suitable for analysis of free $\mathbf{L^3}$ in the sample.

References

1. Jagadish, B.; Brickert-Albrecht, G.L.; Nichol, G.S.; Mash, E.A.; Raghunand, N. On the synthesis of 1,4,7-tris(tert-butoxycarbonylmethyl)-1,4,7,10-tetraazacyclododecane. *Tetrahedron Lett.* **2011**, *141*(17), 2058–2061.
2. Pell, A.J.; Pintacuda, G.; Grey, C.P. Paramagnetic NMR in solution and the solid state. P*rog. Nucl. Magn. Reson. Spectrosc.* **2019**, *111*, 1–271.
3. Sheldrick, G.M. A short history of SHELX. *Acta Cryst. A* **2014**, *64*, 112–122.
4. Sheldrick, G.M. Crystal structure refinment with SHELXL. *Acta Cryst. C* **2015**, *71*, 3–8.
5. Dolomanov, O.V.; Bourhis, L.J.; Gildea, R.J.; Howard, J.a.K.; Puschmann, H. OLEX2: a complete structure solution, refinement and analysis program. *J. Appl. Cryst.* **2009**, *42*, 339–341.
6. David N. Mastronarde Automated electron microscope tomography using robust prediction of specimen movements. *J. Struct. Biol.* **2005**, *152*(1), 36–51.
7. Petříček, V.; Palatinus, L.; Plášil, J.; Dušek, M. Jana2020 – a new version of the crystallographic computing system Jana. *Z. für Krist. - Cryst. Mater.* **2023**, *238*, 7–8, 271–282.
8. Ruiz-Martínez, A.; Casanova, D.; Alvarez, S. Polyhedral structures with an odd number of vertices: nine-coordinate metal compounds. *Chem. Eur. J.* **2008**, *14*, 1291–1303.
